# Expanding the Circuitry of Pluripotency by Selective Isolation of Chromatin-Associated Proteins

**DOI:** 10.1016/j.molcel.2016.09.019

**Published:** 2016-11-03

**Authors:** Mahmoud-Reza Rafiee, Charles Girardot, Gianluca Sigismondo, Jeroen Krijgsveld

**Affiliations:** 1 German Cancer Research Center (DKFZ), Im Neuenheimer Feld 581, 69120 Heidelberg, Germany; 2 Excellence Cluster CellNetworks, Heidelberg University, 69120 Heidelberg, Germany; 3 European Molecular Biology Laboratory (EMBL), Genome Biology Unit, Meyerhofstrasse 1, 69117 Heidelberg, Germany

**Keywords:** pluripotency, chromatin, protein interactions, proteomics, biotinylation, embryonic stem cells, reprogramming

## Abstract

Maintenance of pluripotency is regulated by a network of transcription factors coordinated by Oct4, Sox2, and Nanog (OSN), yet a systematic investigation of the composition and dynamics of the OSN protein network specifically on chromatin is still missing. Here we have developed a method combining ChIP with selective isolation of chromatin-associated proteins (SICAP) followed by mass spectrometry to identify chromatin-bound partners of a protein of interest. ChIP-SICAP in mouse embryonic stem cells (ESCs) identified over 400 proteins associating with OSN, including several whose interaction depends on the pluripotent state. Trim24, a previously unrecognized protein in the network, converges with OSN on multiple enhancers and suppresses the expression of developmental genes while activating cell cycle genes. Consistently, Trim24 significantly improved efficiency of cellular reprogramming, demonstrating its direct functionality in establishing pluripotency. Collectively, ChIP-SICAP provides a powerful tool to decode chromatin protein composition, further enhanced by its integrative capacity to perform ChIP-seq.

## Highlights

• ChIP-SICAP isolates and identifies proteins that colocalize on chromatin
• Chromatin composition around Oct4, Sox2, and Nanog depends on the pluripotent state
• Trim24 is part of the pluripotency network and promotes reprogramming
• ChIP-SICAP allows recovery of DNA for sequencing with ChIP-seq quality

## Graphical Abstract

**Figure:**
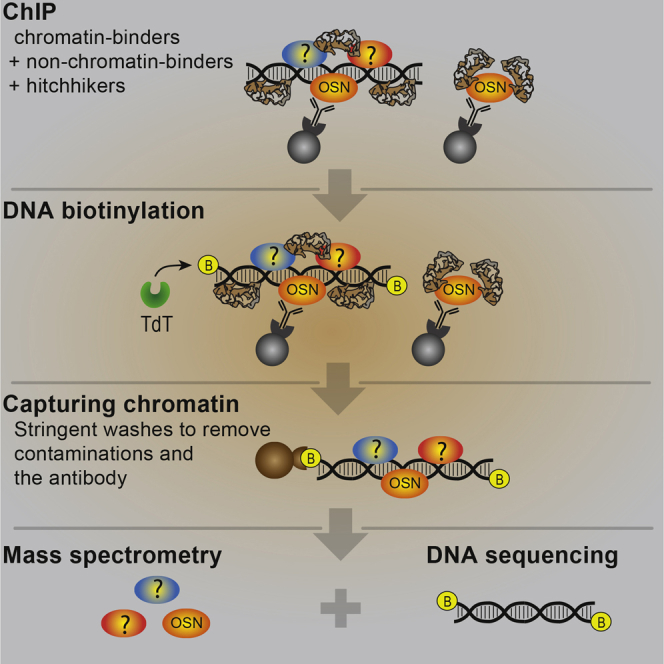

## Teaser

Rafiee et al. introduce ChIP-SICAP as a novel method to identify chromatin-bound proteins that colocalize with a bait protein on DNA. Applied to Oct4, Sox2, and Nanog in ESCs, they identify Trim24 as a novel protein that is functionally involved in the pluripotency network by promoting cellular reprogramming.

## Introduction

In ESCs, the three master transcription factors Oct4, Sox2, and Nanog constitute the core transcriptional circuitry (Boyer et al., 2005, Loh et al., 2006), which on the one hand promotes the expression of pluripotency genes, while on the other hand suppresses lineage commitment and differentiation (Boyer et al., 2006, Laugesen and Helin, 2014, Lee et al., 2006). In mouse ESCs, pluripotency can be further reinforced by replacing serum in conventional culture medium with two kinase inhibitors (2i), PD0325901 (inhibiting mitogen-activated protein kinase, Mek) and CHIR99021 (inhibiting glycogen synthase kinase-3, Gsk3), driving the ESCs into a condition resembling the preimplantation epiblast (Nichols and Smith, 2009, Ying et al., 2008). Hence, cells grown in 2i medium are considered as an in vitro representation of the ground state of pluripotency.

Transcriptome analysis indicated that most of the pluripotency-associated transcription factors did not change significantly in expression level between serum and 2i conditions (Marks et al., 2012), suggesting that additional proteins may sustain the functionality of core pluripotency factors in 2i. Since transcription factors, including pluripotency TFs, execute their function in chromatin, we aimed to identify proteins that associate with OSN in their DNA-bound state as opposed to interactions that may occur in soluble form. Despite the large diversity of available methods to identify protein interactions (reviewed by Dunham et al., 2012), very few of them differentiate between interactions that depend on the subcellular location. This is a critical shortcoming, especially for proteins that dynamically change location, either between or within organelles (e.g., nucleosol or chromatin bound). Indeed, transcription factors have been shown to form different complexes on and off chromatin, as demonstrated for several FOX proteins (Li et al., 2015). To specifically identify proteins in their DNA-bound state, we therefore developed a method for the *s*elective *i*solation of *c*hromatin-*a*ssociated *p*roteins (SICAP). SICAP captures an endogenous protein under ChIP conditions and then biotinylates DNA, allowing the specific isolation of DNA-bound proteins on streptavidin beads, followed by mass spectrometric protein identification. Thus, by design, ChIP-SICAP identifies chromatin-bound proteins in the direct vicinity of the bait protein on a short stretch of DNA (between 200 and 500 bp). Here we introduce and evaluate ChIP-SICAP and apply it characterize the chromatin-bound network around Oct4, Sox2, and Nanog in mouse ESCs. We demonstrate the power of ChIP-SICAP by the discovery of Trim24 as a component of the pluripotency network.

## Design

Many studies have been devoted to defining interactomes of pluripotency factors (Huang and Wang, 2014), most of which are based on coimmunoprecipitation (coIP) of Flag- or HA-tagged TFs, such as for Oct4 (Pardo et al., 2010, van den Berg et al., 2010), Sox2 (Lai et al., 2012, Mallanna et al., 2010), and Nanog (Gagliardi et al., 2013). The general limitation of these approaches is their need to introduce an affinity tag, often using an exogenous expression system. Studying protein interaction in the context of chromatin adds a number of other challenges, especially since chromatin is highly insoluble. To promote solubilization of chromatin, DNA can be fragmented, e.g., as carried out by sonication in ChIP protocols, combined with crosslinking to maintain protein-DNA interactions. Hence, different variations of ChIP protocols have been developed to study protein interactions on chromatin, including modified ChIP (mChIP; Lambert et al., 2009), ChIP-MS (Engelen et al., 2015, Ji et al., 2015), and rapid immunoprecipitation mass spectrometry of endogenous proteins (RIME) (Mohammed et al., 2016). ChIP-MS and RIME both apply mass spectrometric analysis on proteins immunoprecipitated from formaldehyde-crosslinked cells, but they differ in the fact that they digest proteins directly on the protein A beads (RIME) or after elution (ChIP-MS). Yet, a number of issues limit the practical utility of these methods to specifically enrich for chromatin-bound proteins. First, they often suffer from the copurification of contaminating proteins that have been referred to as “hitchhikers” (Ohta et al., 2010) to indicate their avid binding to the highly charged backbone of DNA, and other contaminants that are commonly observed in affinity-purification experiments (e.g., ribosomal proteins, hnRNPs), as documented in the CRAPome (Mellacheruvu et al., 2013). Another often-marginalized problem is that the antibody used for affinity purification represents a huge contamination in subsequent mass spectrometry, thereby masking lower abundance proteins. Finally, and maybe most importantly, none of the presented methods discriminate protein interactions occurring on and off chromatin.

BioTAP-XL (Zee et al., 2016) and a method coined as “chromatin-interaction protein MS,” confusingly also abbreviated as ChIP-MS (Wang et al., 2013), tag a given protein with protein A or a His tag along with a biotin-acceptor sequence. Although this allows for stringent washing after capture on streptavidin beads, introduction of the tag may alter the functionality or expression level of the protein, while requiring a cloning step that may not be suitable or desirable for all cell types.

**Figure 1:**
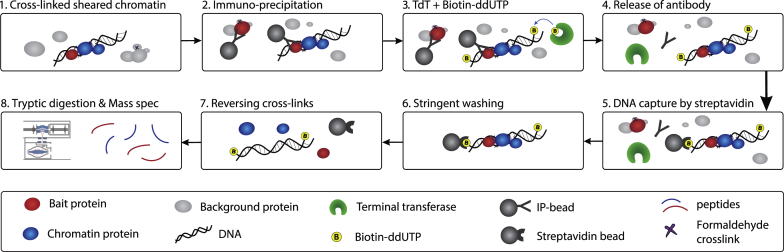
Principles of ChIP-SICAP Similar to a ChIP experiment, DNA proteins are crosslinked by formaldehyde, and fixed chromatin is sheared to small fragments by sonication (1). Following immunoprecipitation with a suitable antibody (2), DNA is biotinylated by TdT and biotin-ddUTP (3). The antibody is denatured by SDS (4), and chromatin is retrieved along with interacting proteins on streptavidin beads (5). Following extensive washing (6), isolated chromatin fragments are heated to reverse the crosslinks (7). Finally, proteins are digested and identified by mass spectrometry (8).

Because of these limitations in available approaches, we here introduce a method termed “selective isolation of chromatin associated proteins” (SICAP), which we combine with ChIP (ChIP-SICAP) to specifically purify, identify, and quantify the protein network around a chromatin-bound protein of interest (Figure 1). ChIP-SICAP combines the advantages of the aforementioned methods while bypassing their limitations, in that it targets endogenous proteins, does not require protein tagging or overexpression, uses formaldehyde for chromatin crosslinking, and allows very stringent washing, including removal of the antibody. Furthermore, ChIP-SICAP uniquely benefits from the double purification of protein-DNA complexes, accomplished by subsequent ChIP of the protein of interest, and an innovative step to biotinylate DNA allowing capture and stringent washing of the protein-DNA complex.

ChIP-SICAP (Figure 1) starts from crosslinked and sheared chromatin using established ChIP procedures (Nelson et al., 2006), followed by addition of a suitable antibody and capture of the protein-DNA complex on protein A beads. The key step of ChIP-SICAP is then the end labeling of DNA fragments with biotin by terminal deoxynucleotidyl transferase (TdT) in the presence of biotinylated nucleotides. TdT is a template-independent DNA-polymerase-extending DNA 3′ end regardless of the complementary strand, which is also used in the so-called TUNEL assay to detect double-stranded DNA breaks in apoptotic cells (Jones and Dive, 1999). Next, addition of ionic detergents (7.5% SDS) and a reducing agent disassembles all protein interactions (except those crosslinked to DNA), denatures the antibody, and releases chromatin fragments. Biotinylated DNA-protein complexes are then captured on streptavidin beads, followed by a number of stringent washes (subsequently with 1% SDS, 2M NaCl, 20% isopropanol, and 40% acetonitrile) to effectively remove contaminating proteins and the IP antibody. Finally, protein-DNA crosslinks are reversed by heating, and proteins are proteolytically digested for MS-based identification (Figure 1). As a result, ChIP-SICAP identifies the proteins that colocalize with the bait on a short fragment of chromatin.

## Results

### End Labeling of DNA Significantly Improves Purification of Chromatin-Associated Proteins

**Figure 2:**
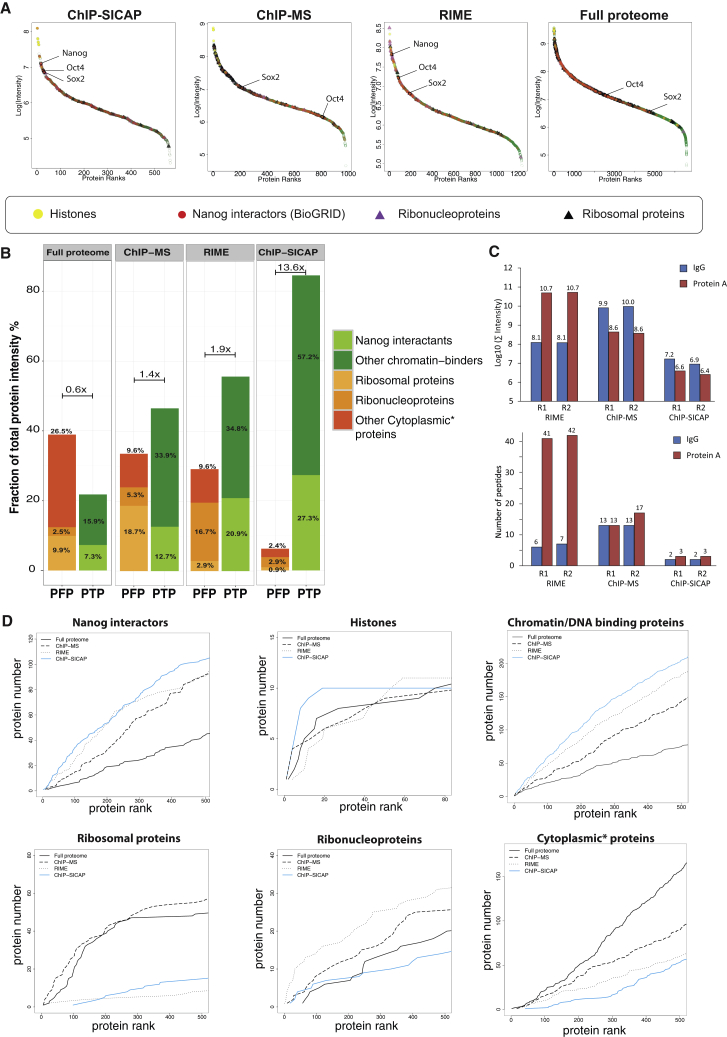
Comparing Performance of ChIP-SICAP to ChIP-MS and RIME Using Nanog as the Bait (A) Enriched proteins ranked by abundance. (B) Relative abundance of various protein classes. Stacked bars show the abundance of Nanog interactors (light green), other chromatin/DNA binding proteins (dark green), ribosomal proteins (amber), ribonucleoproteins (orange), and other cytoplasmic proteins (burnt orange) relative to the total protein abundance within each method. PTP, potential true positive. PFP, potential false positive. (C) Total MS intensity (top panel) and number of peptides (bottom panel) produced from antibody and protein A contamination. (D) Cumulative distribution of abundance-ranked proteins within the four datasets for various protein classes. Asterisk indicates cytoplasmic proteins that are neither Nanog interactor nor chromatin/DNA binder nor ribosomal nor ribonucleoprotein. See also Figure S2 and Table S1.

To evaluate the performance of ChIP-SICAP, we targeted Nanog as the bait protein in mouse ESCs and performed a comparative analysis with a no-antibody control (noAB) using differential SILAC labeling. In two independent ChIP-SICAP assays, we reproducibly identified 634 proteins, of which 567 were enriched in comparison to the negative control (Nanog/noAB >2-fold in both replicates; Figure S2 and Table S1). Reassuringly, ranking the enriched proteins by their estimated abundance (based on MS peak area) revealed histones and Nanog itself as the most abundant proteins (Figure 2A). This indicates the clear enrichment of chromatin and confirms the specificity of the used antibody. In addition, Oct4 and Sox2, two well-known Nanog interactants, were also among the top-enriched proteins. Proteins of lower intensity include many other known interaction partners of Nanog, as well as potential novel candidates (further discussed below). We then evaluated the benefit of DNA-biotinylation by repeating the same experiment, but omitting the TdT-mediated end labeling of DNA, in two slightly different procedures using protocols as described for RIME (Mohammed et al., 2016) and ChIP-MS (Engelen et al., 2015, Ji et al., 2015). Under ChIP-MS conditions, we identified 981 enriched proteins (out of 1,044 detected with both replicates), i.e., twice the number obtained from ChIP-SICAP (Figures 2A and S2). Using RIME (Figure S1), i.e., digesting proteins on-bead rather than after reversal of crosslinking, we identified 1,232 enriched proteins (out of 1,609 detected by both replicates). Apart from this even further increased number of proteins, ribonucleoproteins (RNPs) now outcompeted histones as the most abundant proteins (Figure 2A). In ChIP-MS, Nanog was identified only in one replicate, while both in ChIP-MS and RIME Oct4 and Sox2 ranked much lower compared to ChIP-SICAP (Figure 2A), possibly as the result of copurification of contaminant proteins.

We next performed a rigorous analysis on these datasets to assess the performance and specificity of the three methods to enrich for chromatin-bound proteins. First, a Gene Ontology (GO) analysis revealed RNA processing and translation as the top-enriched biological processes (BPs) in the ChIP-MS and RIME (Figure S2C), reflecting the presence of many ribosomal proteins, hnRNPs, and splicing factors (Tables S1F and S1G). These proteins are often observed to copurify nonspecifically in affinity-purification procedures, and indeed they feature prominently in the CRAPome database (Mellacheruvu et al., 2013). This suggests that these are contaminant proteins not likely to be related to the Nanog network, although we cannot exclude that some individual RBPs can associate with chromatin. In contrast, processes related to chromatin and transcription are enriched in the ChIP-SICAP dataset (Figure S2C,), while RNA processing ranked only 17th (Table S1E). This indicates that ChIP-SICAP more specifically enriches for proteins that reflect the known function of Nanog in transcriptional regulation.

We next evaluated the presence of 278 proteins previously reported to interact with Nanog in multiple coIP studies, collected in BioGrid. Of these, 109 were identified by ChIP-SICAP (out of 567 detected proteins, 19%) (Figure S2F; Table S1H), compared to 132 proteins (13.5% of the 981 detected proteins) and 156 proteins (12.5% of the 1,232 detected proteins) in ChIP-MS and RIME, respectively. Although ChIP-SICAP recovers fewer known Nanog interactants, their proportion among the detected proteins is much higher, suggesting a higher precision of ChIP-SICAP over ChIP-MS and RIME. Although ideally both the absolute and relative number of returned true positives should be maximized in interactome analyses, specificity seems of greater practical utility. An extreme example is our total ESC proteome dataset containing, among 6,500 proteins, 232 Nanog interactants, i.e., with a specificity of 3.5%.

To further compare the performance of each method, we included protein abundance (estimated from MS intensity) as an additional parameter, allowing us to weigh proteins by relative enrichment within each dataset rather than treating all of them equally. Specifically, we summed the MS intensities of Nanog interactome (as defined in BioGRID) and other chromatin/DNA-binding proteins as potential true positives (PTPs). This was normalized for the total protein intensity of the same sample to estimate the relative abundance of PTPs for each method. Similarly, we calculated the ratios for ribosomal proteins and other components of RNA processing (Figure 2B; Tables S1A–S1C) as well as cytoplasmic proteins as representatives of potential false positives (PFPs). In doing so, 27% of protein intensity in ChIP-SICAP is represented by Nanog-interacting proteins, more than in any of the other datasets (Figure 2B). In addition, other chromatin-binding proteins add another 57% of intensity, collectively accounting for 85% of the total amount of protein recovered by ChIP-SICAP, compared to 47% and 55% in ChIP-MS and RIME, respectively (Figure 2B). Conversely, ChIP-SICAP better removes common contaminants and other cytoplasmic proteins, accounting for 7% of the protein intensity, compared to 29% and 33% in RIME and ChIP-MS, respectively. Taking the intensity ratio of PTPs and PFPs as a proxy for the specificity of each method, ChIP-SICAP (ratio 13.6) scored significantly better than RIME (1.9) and ChIP-MS (1.4) or the total proteome (0.6) as an example of a nonselective method (Figure 2B). Furthermore, stringent washing procedures in ChIP-SICAP resulted in the detection of far fewer peptides originating from IgG (used for IP of Nanog) and protein A (used for capture of the immunoprecipitated complex) (Figure 2C), resulting in an overall reduction of these contaminating proteins between 10- and 10,000-fold in ChIP-SICAP compared to RIME and ChIP-MS (Figure 2C).

We next tested to what extent the various protein classes were enriched or depleted not only as a group (Figure 2B) but also as individual proteins. We therefore ranked all proteins in each of the four datasets by abundance, showing that, in ChIP-SICAP, known Nanog interactors, histones, and other chromatin binding proteins accumulate faster among the top-ranked proteins compared to all three other datasets (Figure 2D). Conversely, common contaminants are largely depleted from the top 100 proteins and only appear among the less abundant proteins. This is in contrast to ChIP-MS, where copurifying ribosomal proteins rank as high as in a total proteome analysis, and to RIME, which seems particularly sensitive to contamination by ribonucleoproteins (Figure 2D).

Collectively, our data show that ChIP-SICAP surpasses ChIP-MS and RIME to more specifically enrich for chromatin-bound partners of a bait protein while more effectively removing common contaminants (Figure S1).

### ChIP-SICAP Reveals Chromatin Proteins that Differentially Interact with the Core Circuitry of Pluripotency

**Figure 3:**
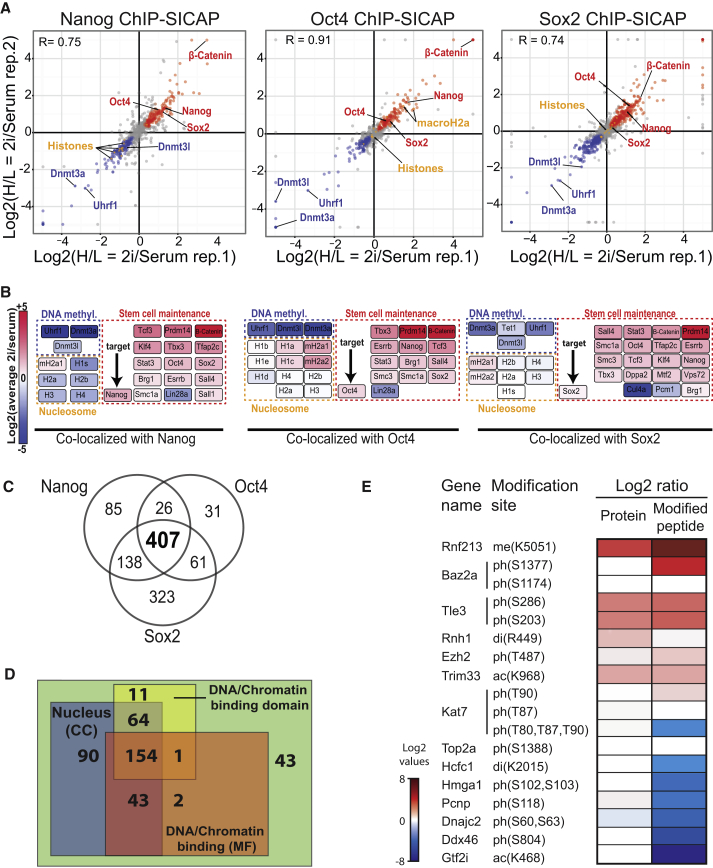
Comparative ChIP-SICAP between 2iL and Serum Conditions (A) Scatterplots indicating the distribution and reproducibility of protein ratios in the three ChIP-SICAP experiments using Nanog, Oct4, and Sox2 as bait proteins. Proteins identified by the no-antibody control are not shown. (B) Differential chromatin interaction of proteins involved in stem cell maintenance, nucleosomes, and de novo DNA methylation among the three ChIP-SICAP experiments. (C) Overlap among proteins identified to colocalize with the three pluripotency master regulators. Proteins identified with no antibody control were subtracted. (D) GO annotation of the proteins identified by ChIP-SICAP with Oct4, Sox2, and Nanog. (E) Fold change of proteins and their modifications, comparing ChIP-SICAP data between 2iL and serum growth conditions. See also Figure S3 and Table S2.

To more systematically study the composition and dynamics of proteins associated with OSN, we separately carried out ChIP-SICAP for Oct4, Sox2, and Nanog in ESCs grown in serum (light SILAC) and 2i plus LIF (2iL) medium (heavy SILAC). In ChIP-SICAP against Nanog, we detected 666 proteins, of which 296 were significantly different between the 2iL and serum conditions (t test adjusted p value ≤ 0.1) (Figure 3A; Tables S2A–S2C). β-catenin was detected among the most enriched proteins in 2iL condition (>20-fold increase), which is expected because of the inhibition of Gsk3β by CHIR99021 resulting in activation of Wnt signaling and translocation of β-catenin to the nucleus. Other stem cell maintenance factors that preferentially associate with Nanog in 2iL-medium included Esrrb, Klf4, Prdm14, Rex1 (Zfp42), Sall4, Tcf3 (Tcf7l1), Tbx3, Stat3, Smarca4 (Brg1), Tfap2c, and Tfcp2l (Figure 3B). Interestingly, all core-nucleosomal histones interacted less with Nanog in 2iL condition (Figure 3A), suggesting that DNA is more accessible for Nanog in the ground state and suggesting that ChIP-SICAP may also inform on global chromatin structure. This is in line with a recent study (Novo et al., 2016) showing that Nanog can remodel heterochromatin to an open architecture in a manner that is decoupled from its role in regulating the pluripotent state.

Finally, Nanog-bound loci are co-occupied with proteins maintaining DNA methylation (Dnmt3a, Dnmt3l, and Uhrf1) preferentially under serum conditions (Figure 3B), fitting with the model of higher CpG methylation rate in this cellular state.

Performing ChIP-SICAP for Oct4 and Sox2 produced results similar to that of Nanog, but with subtle yet important differences (Figures 3A and 3B; Tables S2A–S2C). In each experiment, all three master TFs—Oct4, Sox2, and Nanog—were identified, thus confirming their tight interconnection. Additionally, many stem cell maintenance factors such as β-catenin, Esrrb, Klf5, Mybl2, Prdm14, Rex1, Sall4, Tcf3, Tbx3, Stat3, and Smarca4 (Brg1), were similarly enriched in 2iL conditions in all three ChIP-SICAP assays, or in serum condition such as Uhrf1 and Dnmt3a (Figures 3A and 3B). In contrast to Nanog ChIP-SICAP, most of the nucleosome components did not show significant changes in Oct4 and Sox2 ChIP-SICAP, with the exception of macroH2A1 and macroH2A2, which preferentially associate with Oct4 (Figures 3A and 3B). The different pattern for these transcriptionally suppressive H2A variants (Buschbeck et al., 2009, Gamble et al., 2010) suggests that in 2iL condition some of the Oct4 targets may be transcriptionally repressed by recruiting macroH2A.

### ChIP-SICAP Reveals Bait-Specific Interactions

We identified 407 proteins in the overlap among the three OSN ChIP-SICAP experiments (Figure 3C), 365 of which (90%) are known to have a chromatin-related function (Figure 3D), indicating that indeed we retrieved the desired class of proteins. To assess the specificity of ChIP-SICAP, and to rule out that the observed proteins were enriched irrespective of the used antibody, we used E-cadherin (Cdh1) as an unrelated bait protein to perform ChIP-SICAP. Although Cdh1 is classically known as plasma membrane protein, its cleavage by α-secretase, γ-secretase, or caspase-3 releases specific C-terminal fragments (CTFs) that translocate to the nucleus and bind to chromatin (Ferber et al., 2008). Following expectations, histones and Cdh1 were the most prominent proteins identified in Cdh1 ChIP-SICAP (Figure S3). In addition, and according to expectation, Cdh1 was identified exclusively by peptides originating from the most C-terminal CTF, along with known nuclear Cdh1 interaction partners β-catenin and δ-catenin (p120) (Ferber et al., 2008). In contrast, the stem cell maintenance factors found in OSN ChIP-SICAP were not identified (Table S2E). Collectively, this demonstrates that ChIP-SICAP reveals target-specific protein-DNA interactions.

### ChIP-SICAP Reveals Changes in Chromatin Proteins and PTMs

To investigate whether changes observed in chromatin interactions around OSN were dependent on global protein expression level, we performed a total proteome comparison of ESCs grown in 2iL and serum conditions. Interestingly, protein ratios did not always correlate between in ChIP-SICAP and total proteome. For instance, β-catenin preferentially binds to OSN sites in 2iL versus serum (32-fold higher based on Oct4 and Nanog, and ∼3.3-fold based on Sox2), without a change in overall expression (Tables S2A–S2D; Figure S3C). We observed a similar trend for Esrrb, Kdm3a, Mybl2, Tcf7l1 (Tcf3), Tle3, Sall4, Scml2, Smarcd2, Smarce1, Stat3, Trim24, and Zfp42 (Tables S2A–S2D; Figure S3C). This suggests that alternative mechanisms are in place to induce interaction with chromatin in general, and with the OSN network in particular. Intrigued by the differential chromatin-binding proteins, we analyzed the OSN ChIP-SICAP data for the presence of proteins modified by phosphorylation, acetylation, methylation, and ubiquitination. Indeed, we identified 95 ChIP-SICAP proteins carrying one or more of these modifications (Table S2E). Phosphorylation was the most frequent modification, observed on 84 sites (Figure S3B; Table S2E). Several PTMs differ in abundance between 2iL/serum, mostly following the trend of their cognate protein, with distinct exceptions (Figure 3E) suggesting a change in the stoichiometry of the modification in proteins associating with OSN in 2iL versus serum conditions. Although additional experiments will be required to confirm if these modifications are causally involved in modulating protein interactions in chromatin, ChIP-SICAP may provide a starting point to investigate how PTMs shape chromatin-bound protein networks.

### Trim24 Participates in the Pluripotency Network

**Figure 4:**
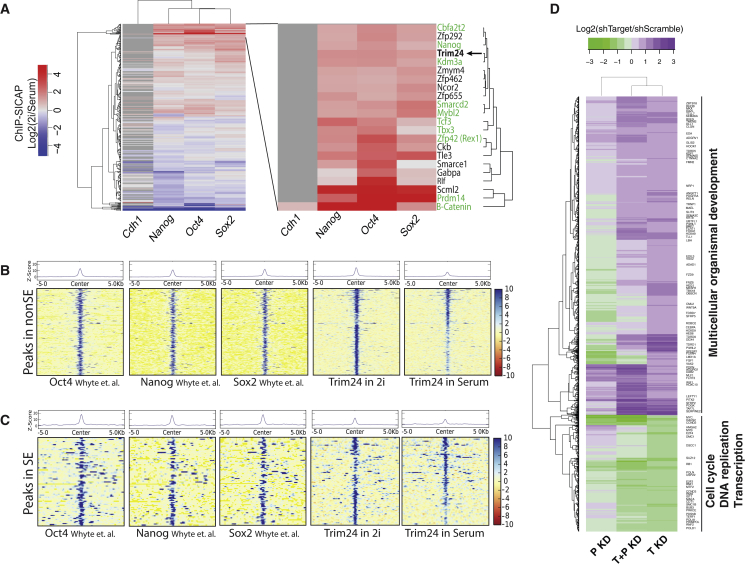
Integration of OSN ChIP-SICAP and Association of TRIM24 to the Pluripotency Network (A) Hierarchical clustering of proteins identified by ChIP-SICAP using Oct4, Sox2, Nanog, and Cdh1 as bait proteins. Coloring was done according to ChIP-SICAP 2iL/serum protein ratio (log2). Zoom-in of the top cluster shows the enrichment of known (green) and so-far-unknown OSN-associated proteins (black). (B) Trim24 ChIP-seq signal in 2iL and serum conditions compared to OSN signal in non-superenhancers (nonSE). (C) Same as in (B), but for superenhancers (SE), as defined in (Whyte et al., 2013). (D) Heatmap showing differentially expressed genes after Trim24 knockdown (fold change > 1.5, FDR < 0.01). T KD, Trim24 knockdown; P KD, p53 knock down; T+P KD, double knockdown of Trim24 and p53. See also Figure S4, Table S3, and Table S4.

The 407 proteins that were consistently enriched with OSN (Figure 3C; Tables S2A–S2D) were subjected to hierarchical clustering based on their ChIP-SICAP protein ratios between 2iL and serum conditions, showing high similarity between Oct4, Sox2, and Nanog experiments while Cdh1 remained as a separate group (Figure 4A). Interestingly, many established stem cell regulators were enriched in 2iL conditions by each of the three TFs (Figure 4A), indicating strong association with the OSN network in the naive pluripotent state. These include Nanog, β-catenin, Prdm14, Zfp42 (Rex1), Tcf7l1(Tcf3), Tbx3, and Kdm3a (Jmjd1a). Interestingly, Cbfa2t2, a transcriptional corepressor not previously known to interact with OSN, was identified very recently as a protein that regulates pluripotency and germline specification in mice by providing a scaffold to stabilize PRDM14 and OCT4 on chromatin (Tu et al., 2016). This is not only fully consistent with our observation of Cbfa2t2 in the OSN network but also provides an independent functional validation of our data.

Another candidate that we identified is Trim24, an E3-ubiquitin ligase that binds to combinatorially modified histones (Tsai et al., 2010). We performed ChIP-seq for Trim24 to identify its genome-wide occupancy in ESCs grown both in 2iL and serum media and compared this to genome occupancy of OSN (Table S3). Overall, Trim24 colocalized with OSN in 813 enhancers (Figures 4B and 4C; Tables S3B and S3C), including 88 of the 142 (62%) previously reported superenhancers (Whyte et al., 2013). Additionally, Trim24 preferentially binds to 237 enhancers in 2iL-condition compared to only 27 in serum condition (FDR <0.05 and fold change >1.5; Tables S3D and S3E), which is in line with the high ChIP-SICAP ratio of Trim24 in 2iL/serum (Figure 4A). Interestingly, some of these enhancers are in close proximity to genes involved in either negative regulation of cell differentiation or positive regulation of cell proliferation (Table S3F), thus suggesting a regulatory role for Trim24 in processes that are fundamental to pluripotency.

### Trim24 Regulates Cell Proliferation and Differentiation Gene Expression in Mouse ESCs

To better understand how Trim24 functions mechanistically in mouse ESCs, we performed knockdown of Trim24 using short hairpin RNA (shRNA) for 24 hr, followed by mRNA sequencing. We observed dysregulation of 1,562 genes (adjusted p value < 0.01 and fold change >1.5) (Figure 4D; Table S4A). Interestingly, developmental genes were upregulated, including genes involved in neural differentiation (e.g., Bdnf, Nrcam, Tgfb2, and Reln), immune system (Fcgr3 and Cd34), muscle differentiation (Myh6 and Myh7), and spermatogenesis (Dazl, Tdrd1, and Piwil2). On the other hand, numerous genes with central roles in cell cycle and proliferation were downregulated, (e.g., Myc, Myb, RB1, CyclinD2, and CyclinD3) (Figure S4F; Table S4B). Remarkably Bmi1, Rnf2, Suz12, and Mtf2 were downregulated, which are well-known members of the PRC1 and PRC2 complexes (Table S4C). Altogether, this result indicates that Trim24 is required to suppress developmental gene, and to maintain expression of genes involved in proliferation, cell cycle, and DNA replication.

**Figure 5:**
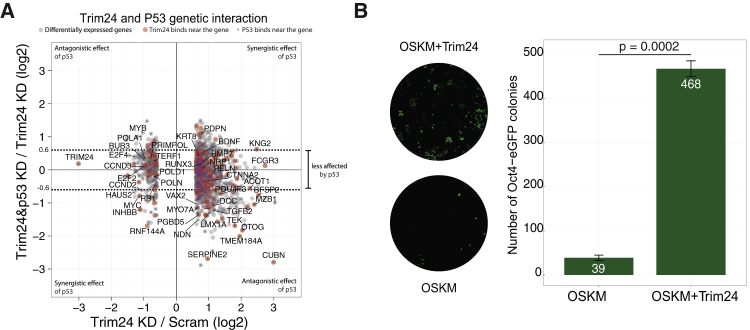
Mechanism and Function of Trim24 (A) Genetic interaction between Trim24 and p53. The scatterplot shows the genes differentially expressed after Trim24 knockdown. The effect of p53 knockdown on Trim24-target genes (y axis) was calculated by dividing expression change after p53-Trim24 double knockdown by expression change after Trim24 knockdown. Red dots indicate genes with a Trim24-binding site (<10 kb from the gene). Blue dots indicate genes with a p53-binding site (<10 kb from the gene). (B) Trim24 increases the efficiency of reprogramming. (Left) iPS colonies were generated with Oct4, Sox2, Klf4, and c-Myc (OSKM) (bottom) or OSKM plus Trim24 (top) to compare the efficiency of reprogramming based on the number of Oct4-EGFP-positive colonies. (Right) Bar chart indicates the mean of three wells transduced by the vectors separately (error bars indicate SD; p value, Student’s t test). See also Figure S5, Table S4, and Table S5.

Previously, Allton et al. have shown that Trim24 knockdown in mouse ESCs leads to p53-mediated apoptosis (Allton et al., 2009). To test coregulation of genes by Trim24 and p53, we carried out p53 knockdown as well as double knockdown of Trim24 and p53. As a result of p53 knockdown, 1,801 genes were deregulated, of which 353 genes were overlapping with Trim24 knockdown (Figure S4D; Table S4A). We compared these data to a Trim24-p53 double knockdown to distinguish synergistic and antagonistic effects (Figure 5A), revealing that 73.4% of the Trim24 target genes are regulated independent of p53. However, the effect of p53 on 18.1% and 8.4% of the Trim24 targets is antagonistic and synergistic, respectively. For instance, p53 has an antagonistic effect on Myb expression, rescuing Trim24 knockdown-mediated downregulation of Myb (Figure 5A; Table S4A). Conversely, p53 and Trim24 have synergistic positive effects on Myc expression.

Among the 1,562 genes that are differentially expressed after Trim24 knockdown, 198 genes (11%) are located near (<10 kb) the Trim24 binding sites on the genome (Figure 5A; Table S4D). Moreover, 68 ESC enhancers with Trim24 occupancies are located near the differentially expressed genes (Figure S4E). The comparison of the genome-wide occupancy of p53 in mouse ESCs (Li et al., 2012b) with our Trim24 ChIP-seq data revealed that 17 ES superenhancers are cobound by p53 and Trim24 (Figure S4B). Remarkably, this includes the superenhancers of pluripotency genes such as Nanog, Prdm14, Sox2, and Tbx3. Although Trim24 binds preferentially to these loci in 2iL media (Figure S4C), knockdown of Trim24 had no significant effect on the expression of these genes, at least under the used conditions (knockdown for 24 hr).

Altogether, these data indicate that Trim24 functions to activate expression of cell cycle, DNA replication, and polycomb components and to suppress expression of developmental genes largely independently of p53.

### Trim24 Significantly Improves the Efficiency of Somatic Cell Reprogramming

Since our observations position Trim24 in the OSN network, regulating the expression of cell cycle and developmental genes, we tested if Trim24 can promote the generation of iPS cells. We coexpressed Trim24 with OSKM in a doxycycline (Dox)-inducible reprogramming system (Stadtfeld et al., 2010) to induce formation of iPS cells from secondary MEFs. As a result, we observed that expression of Trim24 together with OSKM increased the number of Oct4-EGFP-positive colonies from 39 to 468 per plate compared to OSKM alone, i.e., an increase of 12-fold (Figures 5B and S5). This suggests that Trim24 stabilizes the transcriptional program imposed by OSKM to more efficiently establish and maintain pluripotency.

### Recovery of DNA after ChIP-SICAP Permits ChIP-Seq from the Same Sample

**Figure 6:**
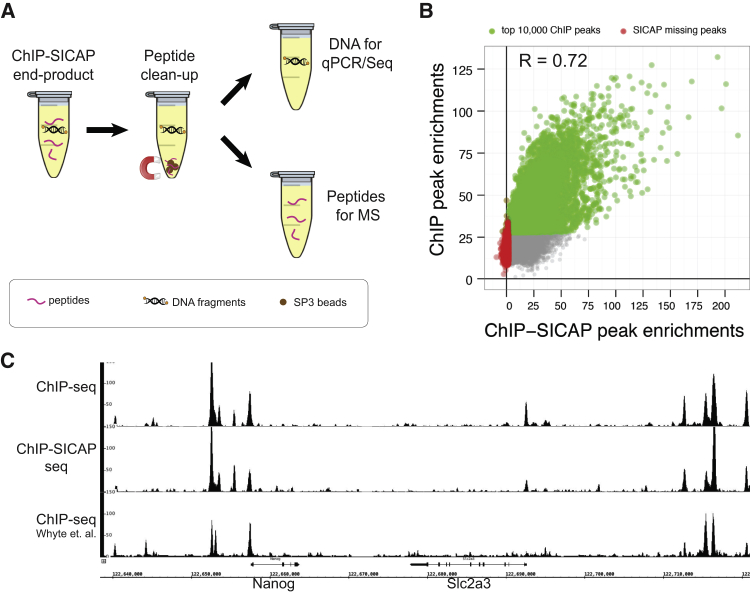
Retrieving DNA and Peptides from the Same ChIP-SICAP Assay (A) After tryptic digestion, peptides are cleaned up by SP3 protocol (Hughes et al., 2014) using magnetic beads. DNA remains in solution while peptides are trapped on the beads and can be retrieved separately for sequencing and mass spectrometry. (B) Enrichment of peaks called in a normal Nanog ChIP-seq in comparison to their enrichment after isolation of DNA via ChIP-SICAP. Green dots indicate top 10,000 enriched peaks. Red dots indicate peaks <2-fold enriched by ChIP-SICAP. (C) Aligned ChIP-seq profiles for Nanog near the Nanog locus. Traces indicate profiles after DNA retrieval via classical ChIP-seq (top) and ChIP-SICAP (middle) compared to ChIP-seq data from Whyte et al. (2013) (bottom). See also Figure S6 and Table S7.

We next investigated the feasibility of retrieving both proteins and DNA after ChIP-SICAP, aiming to identify the proteins that colocalize with the bait (by MS) as well as its genomic binding site (by NGS) from the same sample. We therefore verified the presence of DNA in the supernatant of samples treated with SP3 (Hughes et al., 2014), the last step in the ChIP-SICAP procedure used for peptide cleanup and removal of detergents (Figure 6A). Indeed, qPCR on DNA purified after Nanog ChIP-SICAP recovered the Nanog promoter, but not flanking regions (Figure S6), consistent with the notion that Nanog binds to its own promoter. Next, although the recovered DNA was end-biotinylated, we successfully prepared the library for NGS without any change in Illumina sample prep protocol. Strikingly, when comparing the result of regular ChIP-seq and ChIP-SICAP-seq using the same Nanog antibody, we identified a very similar number of peaks with very large overlap (94%, Figure S6B and Table S6) and similar enrichment (Figure S6C). Among the top 10,000 enriched ChIP-seq peaks, only 33 peaks were not enriched by ChIP-SICAP, indicating that recovery of DNA by biotin labeling and streptavidin purification is very efficient in SICAP. Moreover, the recovery of the major ChIP-seq peaks without the introduction of artifactual peaks suggests that TdT biotinylates chromatin fragments in an unbiased manner. As a result, ChIP-SICAP can be used for the simultaneous analysis of proteins and DNA in an integrative workflow, to obtain highly complementary information on the identity of colocalized proteins as well as genomic binding sites of the bait protein.

## Discussion

We have designed ChIP-SICAP to characterize the proteins that converge on chromatin with a protein of interest in its DNA-bound state, aimed to gain insight in the composition and function of the protein network around transcription factors and transcriptional regulators. We applied ChIP-SICAP to Oct4, Sox2, and Nanog in mouse ESCs to better characterize the protein network operating in the core of pluripotency in a quantitative and context-dependent manner and demonstrated the power of this approach by identifying and validating Trim24 as a protein that physically colocalizes and functionally interacts with core pluripotency factors.

Compared to other methods, ChIP-SICAP benefits from the sequential enrichment of the bait protein and the DNA it is crosslinked to. In particular, TdT-mediated biotinylation of DNA and subsequent capture by streptavidin critically contribute to the specificity of the approach by allowing stringent washing to efficiently remove common contaminants, including the IP antibody (Figure S1), while providing evidence that the bait and colocalizing proteins bind to chromatin. A distinct advantage of ChIP-SICAP over conventional coIP is its ability to identify proteins that colocalize within a short distance on DNA, revealing functional connections between proteins that are not necessarily mediated by direct physical interactions. This is highly relevant in the light of recent data showing that interactions between many cooperative TFs are mediated by DNA (Jolma et al., 2015) rather than direct protein-protein interactions.

Abundance ranking of proteins identified by ChIP-SICAP provides a characteristic signature (Figures 2A and S3A) allowing for quality control of the obtained results. Following histones as the most abundant proteins, the bait protein itself typically ranks among the top candidates, thereby validating the specificity of the antibody and thus satisfying the recommendations that were recently proposed for the quality control of antibodies in affinity-purification strategies (Marcon et al., 2015). This is followed by dozens to hundreds of proteins with lower abundance, which we interpret as proteins that colocalize with the bait at decreasing frequency along the genome. This overall pattern, in combination with the identification of bait-specific protein profiles (Figure 4A) and the underrepresentation of common contaminants (Figure 2B), argues against the possibility of systematic calling of false interactions due to overcrosslinking. Yet we cannot exclude the possibility that some of the interactions reported here may be indirect.

We combined ChIP-SICAP with SILAC labeling, demonstrating both tight interconnectivity between 400 proteins that colocalize around the core pluripotency factors Oct4, Sox2, and Nanog and that the composition of this network depends on the pluripotent state (Figure 3A).

We focused our attention to Trim24 as a protein not known to partake in the pluripotency network but that tightly clustered with well-established pluripotency factors, especially in 2iL conditions (Figure 4A). Trim24, also known as transcriptional intermediary factor 1a (Tif1a), has been identified as a E3-ubiquitin ligase but also as a reader of histone modifications (Tsai et al., 2010). Functionally, Trim24 has been shown to modulate transcription in mouse zygotes, by moving from the cytoplasm to the nucleus and to activate transcription of the embryonic genome (Torres-Padilla and Zernicka-Goetz, 2006). Although Trim24 has never been directly linked to pluripotency, large-scale studies suggest that its expression closely follows the trend of bona fide pluripotency factors showing increased expression during reprogramming both at the transcript (Polo et al., 2012) and the protein level (Benevento et al., 2014, Hansson et al., 2012). Our data demonstrate not only that Trim24 colocalizes to many OSN binding sites in the genome (Figures 4B and 4C) but also that it activates transcription of cell cycle and DNA replication genes while suppressing differentiation genes. These characteristics likely contribute to its role in promoting OSKM-mediated generation of iPS cells (Figure 5B).

Intriguingly, recent studies have correlated elevated expression of Trim24 with poor patient prognosis in various tumor entities (Cui et al., 2013, Li et al., 2012a, Liu et al., 2014, Zhang et al., 2015). Furthermore, ectopic expression of Trim24 induced malignant transformation in epithelial cells (Pathiraja et al., 2015), while its knockdown in colon cancer cells induced apoptosis (Wang et al., 2014). Collectively, this suggests that the main function of Trim24 resides in enhancing cell proliferation, thereby contributing to critical hallmarks both of pluripotency and cancer.

Altogether, we have demonstrated that ChIP-SICAP is a powerful tool to gain a better understanding of transcriptional networks in general, and in pluripotency in particular. Considering that this method can be generically applied to any other cell type or chromatin protein, ChIP-SICAP should prove a useful and versatile tool to identify proteins that associate with a variety of TFs, transcriptional regulators, and posttranslationally modified histones. We anticipate that future use of ChIP-SICAP will extend to the analysis of protein translocation to chromatin as a mechanism to determine cell fate, to investigate the correlation between chromatin-association of TFs and their local histone-PTM landscape, and to examine the role of PTMs in protein association to chromatin. Its utility is further enhanced by the ability to simultaneously obtain DNA for high-quality ChIP-seq, to obtain highly complementary data types (protein colocalization and genome occupancy) in an integrated workflow.

### Limitations

One of the limitations of ChIP-SICAP is the need for a ChIP-grade antibody. Thereby it suffers from the same restriction as ChIP-seq, but with the distinction that the verification of the antibody specificity is an inherent part of ChIP-SICAP data analysis. Therefore, even antibodies against nonclassical chromatin proteins may be tested and validated by ChIP-SICAP. The need for protein-specific antibodies may be bypassed by employing CRISPR/Cas9 technologies to insert an affinity tag (e.g., HA or FLAG) in the coding sequence of the gene of interest. As yet another approach, computational methods such as DeepBind (Alipanahi et al., 2015) may predict the score of binding (here colocalization) for a given protein on the binding sites of the bait, although this is limited to proteins for which a motif is known.

The sensitivity of ChIP-SICAP may be limited by the low efficiency of IP (usually ∼1%) and by limitations in mass spectrometry to detect very low-abundance peptides. Consequently, proteins that colocalize with the bait protein at many genomic locations will be preferentially identified. The power of ChIP-SICAP resides in its unbiased protein identification to thereby suggest novel chromatin factors; however, their frequency and the exact sites of colocalization need to be validated by ChIP-qPCR for individual sites, or by ChIP-seq for global profiling across the genome (as performed in this study for Trim24).

## Experimental Procedures

### Cell Culture and Cell Fixation

Mouse ESCs (46c) were grown feeder free on 0.2% gelatinized cell culture plates in either traditional ES media with serum or 2iL-media (2i+LIF). Chromatin was crosslinked by suspending cells in 1.5% formaldehyde (Pierce) for 15 min, quenched in 125 mM Glycine (Merck), and stored at –80°C until use.

### ChIP-SICAP and Mass Spectrometry

Chromatin from 24 million fixed ESCs sheared by sonication, followed by immunoprecipitation with a suitable antibody. After capture on protein A beads, DNA was biotinylated by TdT in the presence of biotin-11-ddUTP and eluted, and protein-DNA complexes were bound to streptavidin beads. Proteins were digested with trypsin, and resulting peptides were fractionated by high pH reverse-phase chromatography and analyzed using LC-MS on a Orbitrap Velos Pro or Q-Exactive mass spectrometer (Thermo Fisher Scientific). A detailed protocol and details for data analysis can be found in the Supplemental Information.

### ChIP-Seq and Data Analysis

After ChIP on crosslinked and sheared chromatin, protein was digested with Proteinase K, and DNA was purified using phenol/chloroform isoamyl alcohol and then precipitated. The libraries were prepared for Illumina sequencing, and sequencing was carried out by Illumina HiSeq 2000 according to the manufacturer’s protocols.

### Trim24 and P53 Knockdown and RNA-Seq Analysis

Knockdown (KD) of Trim24 and p53 was carried out by the lentiviral vectors shTrim24 (TRCN0000088518) and shTrp53 (TRCN0000310844), respectively (Sigma), in three independent transductions. Forty-eight hours after infection, ESCs were lyzed and RNA was extracted for mRNA-seq (following the standard Illumina TruSeq protocol for library generation) and RT-qPCR.

## Author Contributions

M.-R.R. and J.K. designed the studies and analyzed the data. M.-R.R. performed all experiments. G.S. analyzed mass spectrometry data. C.G. analyzed sequencing data. M.-R.R. and J.K. wrote the manuscript with input from all authors.

## Accession Numbers

Mass spectrometry data are available via ProteomeXchange with identifier PXD003798. ChIP-seq and RNA-seq data are available via ArrayExpress with identifiers E-MTAB-3802 and E-MTAB-4893, respectively.

## Supplemental Information

Document S1. Figures S1–S6 and Supplemental Experimental Procedures

Table S1. Comparing SICAP, ChIP-MS, RIME, and Full Proteome Mass Spectrometry, Related to Figures 2 and S2

Table S2. Comparative ChIP-SICAP between 2i and Serum, Related to Figures 3 and 4A

Table S3. Trim24 ChIP-seq, Related to Figures 4B and 4C

Table S4. Gene Expression after Trim24 Knockdown, Combined with ChIP-seq Peaks, Related to Figures 4D and 5A

Table S5. Trim24 and iPS Generation-Colony Counting, Related to Figure 5B

Table S6. Comparing Nanog ChIP-Seq and ChIP-SICAP-Seq, Related to Figure 6

Document S2. Article plus Supplemental Information
