# Supplementary material for: Expanding the Circuitry of Pluripotency by Selective Isolation of Chromatin-Associated Proteins
Source: Mol Cell. 2016 Nov 3;64(3):624–35. doi: 10.1016/j.molcel.2016.09.019 (PMC5101186; doi:10.1016/j.molcel.2016.09.019)
Supplement: Document S1. Figures S1–S6 and Supplemental Experimental Procedures [file mmc1.pdf]

Molecular Cell, Volume *64*

Supplemental Information

Expanding the Circuitry of Pluripotency by Selective Isolation of Chromatin-Associated Proteins

Mahmoud-Reza Rafiee, Charles Girardot, Gianluca Sigismondo, and Jeroen Krijgsveld

Figure S1

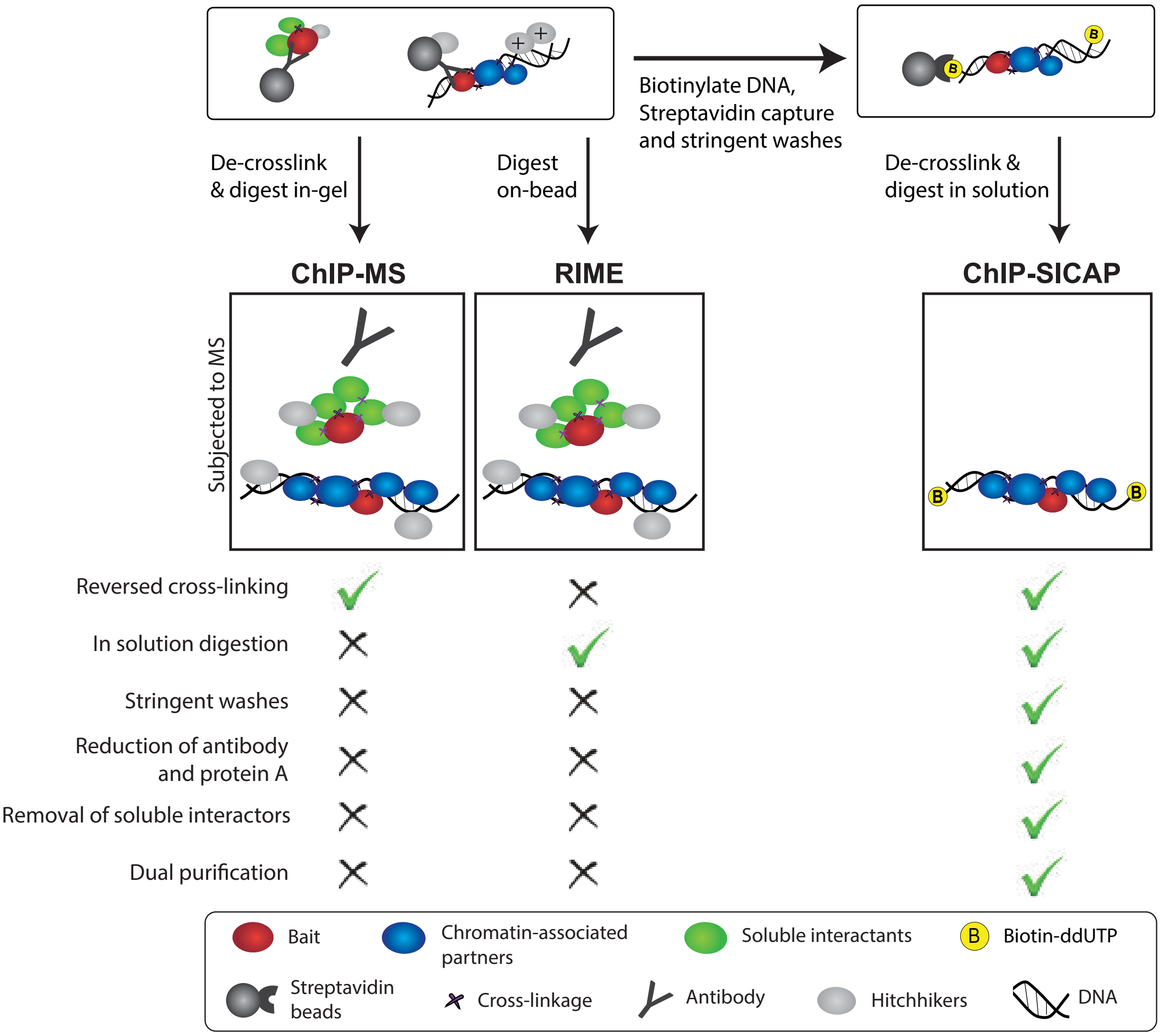
Biotinylate DNA, Streptavidin capture and stringent washes
De-crosslink & digest in-gel
Digest on-bead
De-crosslink & digest in solution
ChIP-MS
RIME
ChIP-SICAP
Subjected to MS
B
Reversed cross-linking
In solution digestion
Stringent washes
Reduction of antibody and protein A
Removal of soluble interactors
Dual purification
Bait
Chromatin-associated partners
Soluble interactants
Biotin-ddUTP
Streptavidin beads
Cross-linkage
Antibody
Hitchhikers
DNA

Figure S2

A

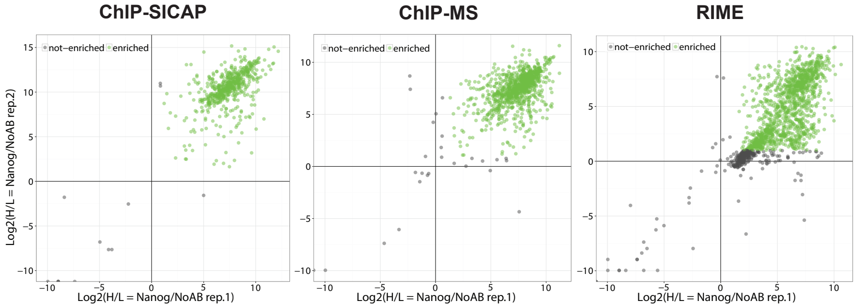

B

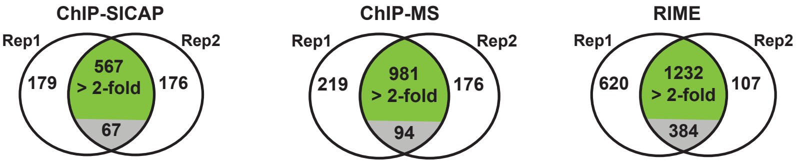

C

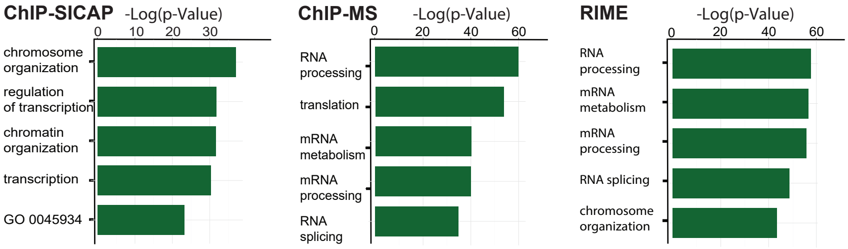

D

Sequence of the IgG peptides	RIME		ChIP-MS		ChIP-SICAP	
	R1	R2	R1	R2	R1	R2
ALPAPIEK	+	+	+	+	+	+
ALPAPIEKTISK	-	-	+	+	-	-
APSVFPLAPCCGDTPSSTVTLGCLVK	-	-	+	+	-	-
DTLMISR	+	+	+	+	+	+
EQQFNSTIR	+	+	+	+	-	-
GDVFTCSVMHEALHNHYTQK	-	-	+	+	-	-
GYLPEPVTVTWNSGTLTNGVR	-	+	-	-	-	-
LSVPTSEWQR	+	+	+	+	-	-
QSSGLYSLSSVVSVTSSSQPVTCNVAHPATNTK	-	-	+	+	-	-
TARPPLREQQFNSTIR	-	-	+	+	-	-
TTPAVLDSDGSYFLYNK	-	-	+	+	-	-
VHNKALPAPIEK	-	-	+	+	-	-
VYTMGPPR	+	+	+	+	-	-
VYTMGPPREELSSR	+	+	+	+	-	-

E

Sequence of the Protein A peptides	RIME		ChIP-MS		ChIP-SICAP	
	R1	R2	R1	R2	R1	R2
ADNNFNK	+	-	-	-	-	-
ADNNFNKEQQNAFYEILNMPNLNEEQR	+	+	-	-	-	-
DDPSQSANLLAEAK	+	+	+	+	+	+
DDPSQSANLLAEAKK	+	+	-	-	-	-
DDPSQSANLLAEAKKLNDAQAPK	+	+	-	-	-	-
DDPSQSANLLSEAK	+	+	-	-	-	-
DDPSQSANVLGEAQK	+	+	+	+	+	+
DDPSQSANVLGEAQKLNDSQAPK	+	+	-	-	-	-
DDPSQSTNVLGEAK	+	+	+	+	+	+
DDPSQSTNVLGEAKK	+	+	-	-	-	-
DDPSQSTNVLGEAKKLNESQAPK	+	+	-	-	-	-
DDPSVSKEILAEAK	+	+	+	+	-	-
DDPSVSKEILAEAKK	+	+	-	-	-	-
DQQSAFYEILNMPNLNEAQR	+	+	-	-	-	-
EILAEAK	+	+	+	+	-	-
EILAEAKKLNDAQAPK	+	+	-	-	-	-
EQQNAFYEILHLPNLNEEQR	+	+	+	+	-	-
EQQNAFYEILHLPNLTEEQR	+	+	-	-	-	-
EQQNAFYEILNMPNLNEEQR	+	+	+	+	-	-
FNKEQQNAFYEILHLPNLNEEQR	+	+	-	-	-	-
KLNESQAPK	+	+	-	-	-	-
KLNESQAPKADNKFNK	+	+	-	+	-	-
KLNESQAPKADNNFNK	+	+	-	+	-	-
LNDAQAPKADNKFNK	+	+	-	-	-	-
LNDAQAPKEEDNNKPGK	+	+	-	-	-	-
LNDSQAPK	+	+	-	-	-	-
LNESQAPKADNKFNK	+	+	-	-	-	-
LNESQAPKADNNFNK	+	+	+	+	-	-
NGFIQSLK	+	+	-	+	-	-
NGFIQSLKDDPSQSANLLAEAK	+	+	+	+	-	-
NGFIQSLKDDPSQSANLLAEAKK	+	+	+	+	-	-
NGFIQSLKDDPSQSANLLAEAKKLNDAQAPK	+	+	-	-	-	-
NGFIQSLKDDPSQSANLLSEAK	+	+	-	-	-	-
NGFIQSLKDDPSQSANVLGEAQK	+	+	+	+	-	-
NGFIQSLKDDPSQSANVLGEAQKLNDSQAPK	+	+	-	-	-	-
NGFIQSLKDDPSQSTNVLGEAK	+	+	+	+	-	-
NGFIQSLKDDPSQSTNVLGEAKK	+	+	-	-	-	-
NGFIQSLKDDPSQSTNVLGEAKKLNESQAPK	+	+	-	-	-	-
NGFIQSLKDDPSVSK	+	+	+	+	-	-
NGFIQSLKDDPSVSKEILAEAK	+	+	-	-	-	-
NGFIQSLKDDPSVSKEILAEAKK	+	+	-	-	-	-
KLNDAQAPK	-	+	-	-	-	-
KLNDAQAPKADNKFNK	-	-	-	+	-	-
NGFIQSLKDDPSQSANLLSEAKK	-	+	-	-	-	-

F

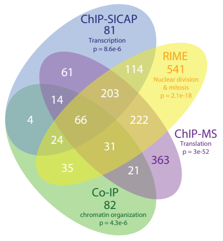

Nanog interactors relative to total IDs

ChIP-SICAP: 19.0%

ChIP-MS: 13.4%

RIME: 12.6%

Figure S3

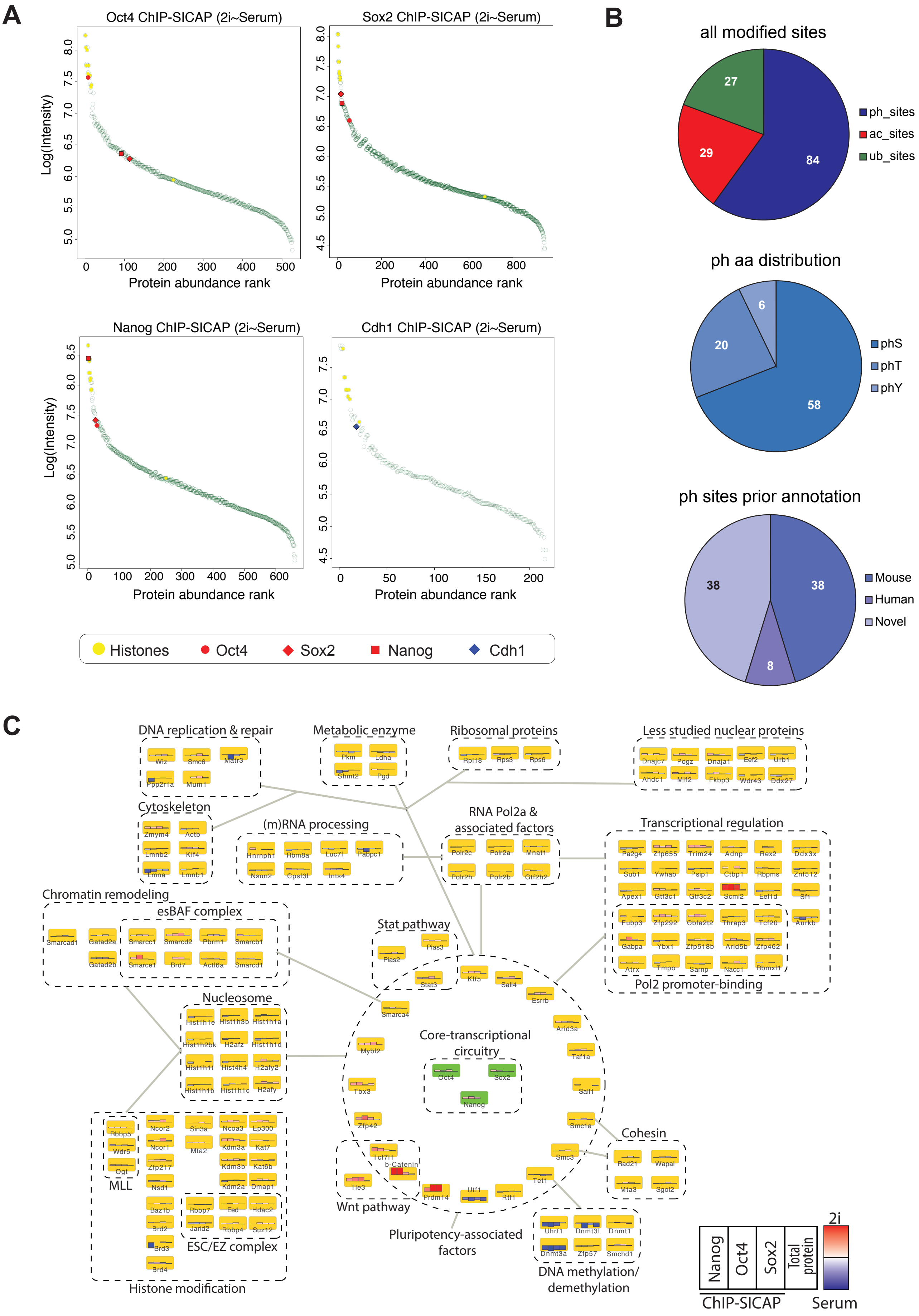
A
Oct4 ChIP-SICAP (2i~Serum)
Sox2 ChIP-SICAP (2i~Serum)
Nanog ChIP-SICAP (2i~Serum)
Cdh1 ChIP-SICAP (2i~Serum)
Log(Intensity)
Protein abundance rank
Histones
Oct4
Sox2
Nanog
Cdh1
B
all modified sites
27
29
84
ph_sites
ac_sites
ub_sites
ph aa distribution
6
20
58
phS
phT
phY
ph sites prior annotation
38
38
8
Mouse
Human
Novel
C
DNA replication & repair
Metabolic enzyme
Ribosomal proteins
Less studied nuclear proteins
Cytoskeleton
(m)RNA processing
RNA Pol2a & associated factors
Transcriptional regulation
Chromatin remodeling
esBAF complex
Stat pathway
Nucleosome
Core-transcriptional circuitry
Oct4
Sox2
Nanog
Pol2 promoter-binding
Cohesin
MLL
Wnt pathway
ESC/EZ complex
Histone modification
Pluripotency-associated factors
DNA methylation/ demethylation
Nanog
Oct4
Sox2
Total protein
ChIP-SICAP
2i
Serum

Figure S4

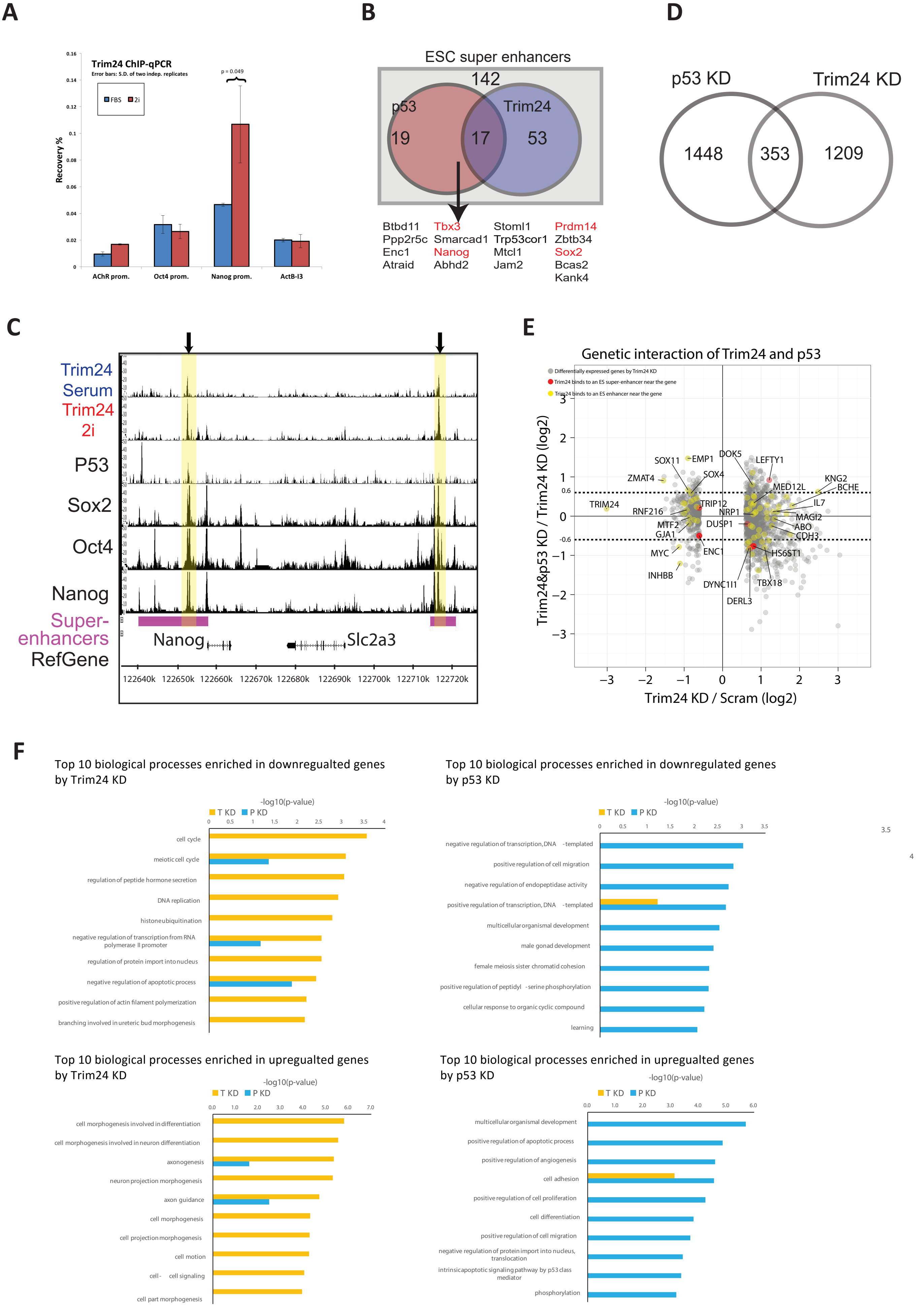
A
Trim24 ChIP-qPCR
Error bars: S.D. of two indep. replicates
FBS
2i
p = 0.049
Recovery %
AChR prom.
Oct4 prom.
Nanog prom.
ActB-I3
B
ESC super enhancers
142
p53
Trim24
19
17
53
Btbd11
Tbx3
Stoml1
Prdm14
Ppp2r5c
Smarcad1
Trp53cor1
Zbtb34
Enc1
Nanog
Mtcl1
Sox2
Atraid
Abhd2
Jam2
Bcas2
Kank4
D
p53 KD
Trim24 KD
1448
353
1209
C
Trim24 Serum
Trim24 2i
P53
Sox2
Oct4
Nanog
Super-enhancers
RefGene
Nanog
Slc2a3
122640k
122650k
122660k
122670k
122680k
122690k
122700k
122710k
122720k
E
Genetic interaction of Trim24 and p53
Differentially expressed genes by Trim24 KD
Trim24 binds to an ES super-enhancer near the gene
Trim24 binds to an ES enhancer near the gene
Trim24&p53 KD / Trim24 KD (log2)
Trim24 KD / Scram (log2)
SOX11
EMP1
DOK5
LEFTY1
ZMAT4
SOX4
KNG2
MED12L
BCHE
TRIM24
TRIP12
IL7
RNF216
NRP1
MAGI2
MTF2
DUSP1
ABO
GJA1
CDH3
MYC
ENC1
HS6ST1
INHBB
DYNC1I1
TBX18
DERL3
F
Top 10 biological processes enriched in downregualted genes by Trim24 KD
-log10(p-value)
T KD
P KD
cell cycle
meiotic cell cycle
regulation of peptide hormone secretion
DNA replication
histone ubiquitination
negative regulation of transcription from RNA polymerase II promoter
regulation of protein import into nucleus
negative regulation of apoptotic process
positive regulation of actin filament polymerization
branching involved in ureteric bud morphogenesis
Top 10 biological processes enriched in downregulated genes by p53 KD
negative regulation of transcription, DNA -templated
positive regulation of cell migration
negative regulation of endopeptidase activity
positive regulation of transcription, DNA -templated
multicellular organismal development
male gonad development
female meiosis sister chromatid cohesion
positive regulation of peptidyl -serine phosphorylation
cellular response to organic cyclic compound
learning
Top 10 biological processes enriched in upregualted genes by Trim24 KD
cell morphogenesis involved in differentiation
cell morphogenesis involved in neuron differentiation
axonogenesis
neuron projection morphogenesis
axon guidance
cell morphogenesis
cell projection morphogenesis
cell motion
cell- cell signaling
cell part morphogenesis
Top 10 biological processes enriched in upregualted genes by p53 KD
multicellular organismal development
positive regulation of apoptotic process
positive regulation of angiogenesis
cell adhesion
positive regulation of cell proliferation
cell differentiation
positive regulation of cell migration
negative regulation of protein import into nucleus, translocation
intrinsic apoptotic signaling pathway by p53 class mediator
phosphorylation

Figure S5

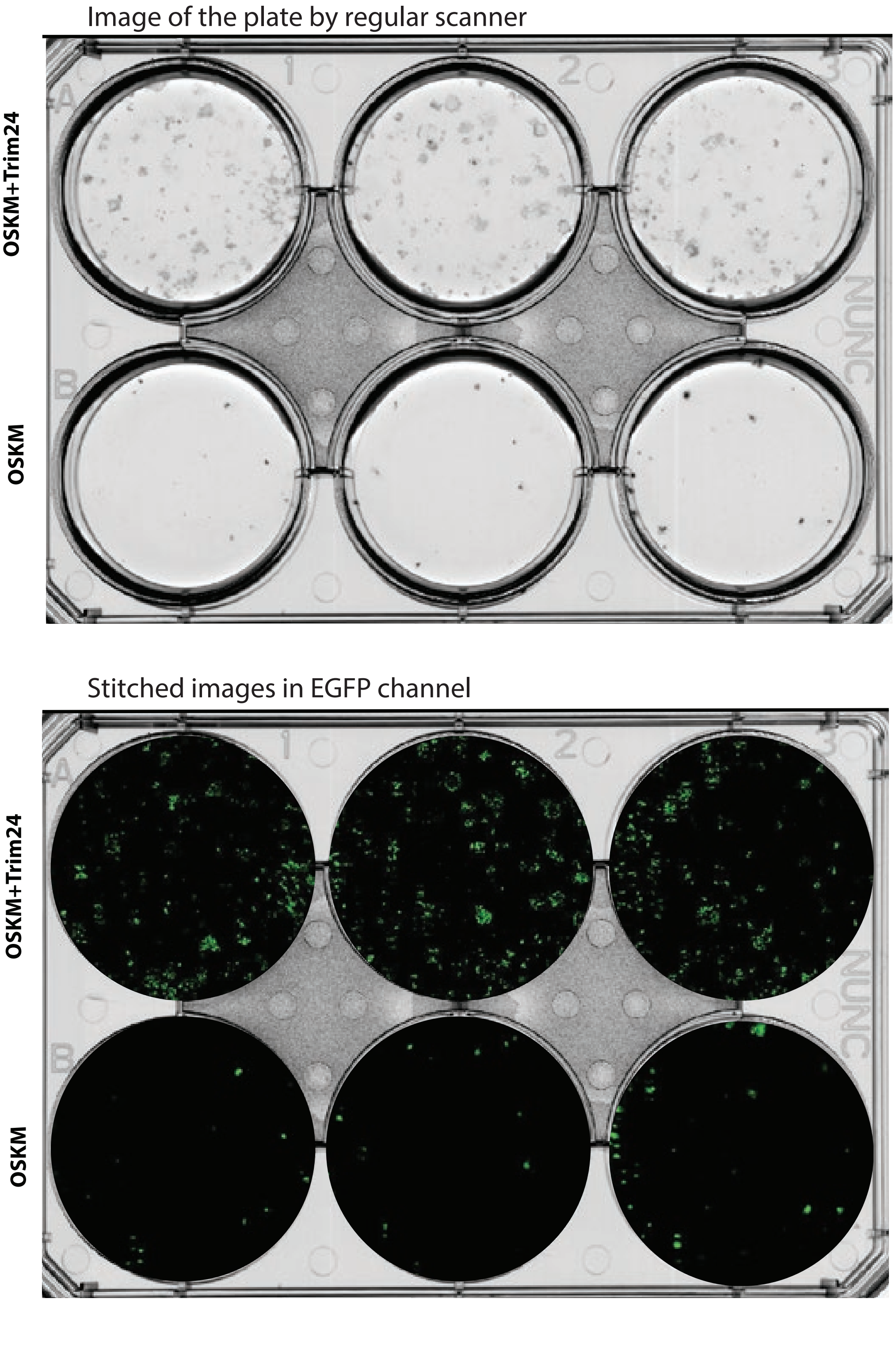
Image of the plate by regular scanner
OSKM+Trim24
OSKM
Stitched images in EGFP channel
OSKM+Trim24
OSKM

Figure S6

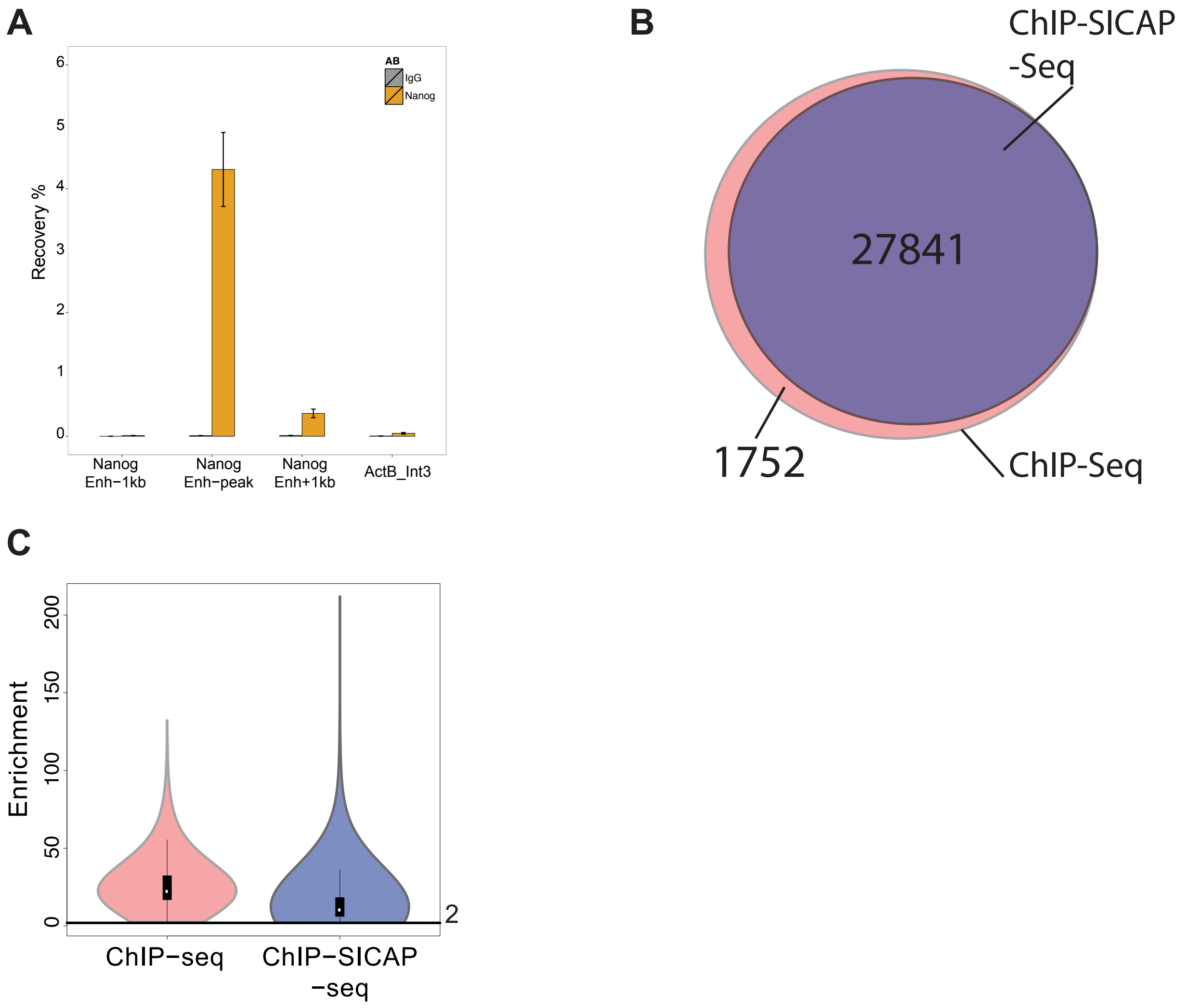
A
B
C
AB
IgG
Nanog
Recovery %
6
5
4
3
2
1
0
Nanog Enh−1kb
Nanog Enh−peak
Nanog Enh+1kb
ActB_Int3
ChIP-SICAP -Seq
27841
1752
ChIP-Seq
Enrichment
200
150
100
50
0
2
ChIP−seq
ChIP−SICAP −seq

Supplemental Figure Legends

Figure S1. Experimental characteristics of ChIP-SICAP, ChIP-MS and RIME

Related to Figure 1

Highlighting the advantage of ChIP-SICAP to enrich for proteins co-localizing with a bait-protein on DNA while depleting for contaminating proteins.

Figure S2. Applying ChIP-SICAP, ChIP-MS and RIME using Nanog and no-antibody control in mouse ES cells

Related to Figure 2

(A) The scatterplots show the fold-enrichment of proteins using a Nanog antibody over a no-antibody control. (B) Venn diagrams showing the overlap between the replicates (numbers in green and grey zones), and the number of the enriched proteins using a 2-fold cut-off (numbers in green colored zone) using ChIP-SICAP, ChIP-MS and RIME. (C) Top-5 enriched GO biological processes for each method. (D) Identified peptides originating from antibody contaminating used for immuno-precipitation. + and – indicate whether or not the peptide was detected in the respective methods. (E) As in D, for peptides originating from proteinA contamination. (F) Venn diagram showing the absolute number of Nanog-interactors (as previously determined by CoIP) identified by each method. The number of Nanog interactors relative to the total number of identified proteins is shown below the Venn diagram. The top-enriched GO biological processes for the non-overlapping proteins are shown in the Venn diagram.

Figure S3. Comparative ChIP-SICAP and full proteome between 2iL and serum

Related to Figure 3.

(A) Abundance-ranked proteins identified by ChIP-SICAP using Oct4 (top-left), Sox2 (top-right), Nanog (bottom-left) and Cdh1 (bottom-right) as bait proteins. (B) Distribution of PTMs identified (ph, ac and ub correspond to phosphorylation, acetylation and ubiquitination, respectively) (top panel), distribution of phosphorylated sites at serine, threonine and tyrosine (middle panel); and grouping of the identified phosphorylated sites indicating whether they had been previously described in mouse, human proteins (bottom panel). (C) Network visualization of the proteins identified by OSN SICAP with significant changes (adj. p-value <0.1 in at least one of the OSN ChIP-SICAP assays). For each protein, the first three bars indicate the protein ratio in 2iL/serum obtained by SICAP using antibodies for Oct4, Sox2 and Nanog, respectively. The fourth bar shows the protein ratio in the total proteome between 2iL and serum conditions.

Figure S4. Validation of Trim24 by ChIP-seq and the effect of Trim24 knock down

Related to Figure 4.

(A) The bar chart indicates the binding of Trim24 to Nanog promoter preferentially in 2iL media. Prom.: promoter <1kb from the TSS. I3: Intorn 3. Error bars indicate standard deviations of two independent replicates. (B) The Venn diagram shows the super-enhancers encompassing Trim24 (this study) and p53-binding sites (Li et al., 2012). The genes in the vicinity of the overlapping super-enhancers are shown, including the most critical pluripotency genes shown in red. (C) The traces show the ChIP-seq profiles of Trim24 (in 2iL and serum), p53, Oct4, Sox2 and Nanog near the Nanog locus. Aforementioned proteins co-localize on the super-enhancer upstream of the

Nanog locus (yellow highlights). (D) Overlap of differentially expressed gene sets found after Trim24 knockdown and p53 knockdown. (E) Scatterplot showing the genes differentially expressed after Trim24 knockdown, and their co-regulation by p53 (as in Fig 5a). Red and yellow dots indicate situations where Trim24 binds to a super-enhancer or to a non-super-enhancer near the gene, respectively. (F) Top-10 enriched GO terms of differentially expressed genes after Trim24 and p53 knockdown (left and right column, respectively) among the down-regulated (top row) and the up-regulated (bottom row) genes.

Figure S5. The effect of Trim24 on the efficiency of reprogramming

Related to Figure 5

Top, a surface scan of the plate was taken. Bottom, the stitched images in EGFP channel are shown for each well. OSKM: induction of Oct4, Sox2, Klf4 and c-Myc. To see the details of the colonies please refer to Table S6.

Figure S6. **Recovering DNA after ChIP-SICAP for Nanog**

Related to Figure 6

(A) qPCR after ChIP-SICAP, showing the enrichment of Nanog at the Nanog enhancer compared to regions ± 1 kb away. ActB_Int3: Intron 3 of ActB. Error bars indicate standard deviations of 2 independent replicates. (B) Venn diagram comparing the overlap in peaks detected by ChIP-seq after recovery of DNA via regular ChIP-seq (red) and ChIP-SICAP-seq procedures (blue). (C)

Violinplot indicating the density of the enriched peaks after recovery of DNA via regular ChIP-seq (red) and ChIP-SICAP-seq procedures (blue).

Supplemental Tables

Table S1_Comparing SICAP, ChIP-MS, RIME and full proteome mass spec, related to Figure 2 and Figure S2

Table S2_Comparative ChIP-SICAP between 2i and serum, related to Figures 3 and 4A

Table S3_Trim24 ChIP-seq, related to Figure 4B and 4C

Table S4_gene expression after Trim24 knockdown, combined with ChIP-seq peaks, related to Figures 4D and 5A

Table S5_Trim24 and iPS generation-colony counting, related to figure 5B

Table S6. Comparing Nanog ChIP-seq and ChIP-SICAP-Seq, related to Figure 6

Supplemental Experimental Procedures

Detailed ChIP-SICAP protocol

Required materials

- Formaldehyde (Methanol-free, Pierce 28906, or 28908)
- IP buffer: Triton 1%, NP40 0.5%, Tris.Cl pH= 7.5-8 50mM, EDTA 5mM, NaCl 150mM
- BW2x buffer : Tris.Cl pH = 8.0, 10mM, EDTA = 1mM., 0.1% TritoneX100, NaCl 2000mM
- Elution buffer: SDS 7.5% + DTT 200mM in H2O
- SDS wash buffer: Tris.Cl 10mM, EDTA 1mM, SDS 1%, NaCl 200mM
- 2-propanol wash buffer: 2-propanol 20% in water
- Acetonitril wash buffer: Acetonitril 40% in water
- Complete protease inhibitor (PI) 50x (Roche app.): dissolve 1 tablet in 1ml ddH2O
- TE buffer: Tris-Cl(10mM, pH~ 7.5), EDTA (1mM)
- Tris buffer: Tris-Cl(10mM, pH~ 7.5)
- TdT (Fermentas, Thermo Scientific, EP0162)
- Biotin-ddUTP (Jenabioscience, NU-1619-BIOX-S)
- Streptavidin Magnetic Beads (NEB, S1420S)
- StemPro Accutase (A1110501, Thermofisher, Life tech)
- Dynabead protein-A or protein-G (Thermofisher, Life tech)
- Sera-Mag magnetic beads (65152105050250 and 45152105050250), GE Healthcare
- Agencourt AmpureXP beads (Beckmancoulter)
- Digestion buffer: Ammonium bicarbonate 50mM, SDS 0.1%

Experimental procedure:

1- Detach the cells by Accutase or any other method for splitting the cells. Then count the cells.
2- Spin the cells 1000g, 5min. Then discard the media.

3- Cross-link DNA-protein complexes by resuspending the cells in PBS + formaldehyde (1.5% v/v final conc.). For every 10 million cells, add 10ml of the formaldehyde solution.
    - The cells should be completely resuspended.
4- Invert the tubes several times within 14min.
5- Add Glycine (fincal conc. 120mM) to stop the reaction. Again Invert the tubes several times within 5min.
6- Spin the cells at 2000g, 2min. Then discard the liquid.
7- Resuspend the cells with PBS in max. vol. of the tube
8- Spin the cells at 2000g, 2min. Then discard the liquid.
9- Resuspend the cells with PBS, and count the cells again.
10- Pour 24 million cells in a 15-ml tube, and spin the cells at 2000g, 2 min
11- For each replicate you may need 24 million cells (or at least 8 million).
    - The negative control for this assay is a No-antibody or normal IgG. The best negative control is knockout control, which is not always available. No-biotin control is not needed, as it is always super clean.
    - After cell pellets could be frozen at stored -80 for months.

12- Mix TE buffer (10mM, pH~ 7.5) + Complete protease inhib. (final conc. is 2x).
13- Resuspend the cells in 1ml of TE vortexing, and transfer them into a 2-ml micro-tube
    - If you are going to compare 2 cellular states that are labeled by SILAC (e.g. 2iL and serum state of mouse ES cells) you may mix the cells from the beginning.
    - If you have a target of interest, and you wish to compare it with the negative control, you may mix the samples after the end-labeling of DNA, as it is mentioned subsequently.
14- Spin 1000g, 2min
15- Discard the supernatant.
16- Resuspend again by vortexing in 0.9ml of TE buffer
17- Put the micro-tubes for 10 min on ice
18- Add 0.1ml TritoneX100 10% (the final conc. is 1%), vortex, and keep 5min on ice
19- Spin 1000g, 2min
20- Discard supernatant

21- Resuspend again by vortexing in 1ml TE+ Complete PI (2x),
22- Spin 1000g, 2min
23- Discard supernatant
24- Resuspend again by vortexing in 1ml TE+ Complete PI (2x),
25- Spin 500g, 2min
26- Resuspend each 24million cells in ~700ul TE+ Complete PI (2x)
   - the final vol. should be ~780ul, so then each 24million cells is divided into 6x130ul to be sonicated.
27- Pour 130ul of the cell suspension in a Covaris micro-TUBE
28- Sonication for mouse ES cells using Covaris S220 was carried out as follows:
   - Time: 430s, Duty cycle: 10%, Intensity: 5, Cycle/Burst: 200
   - Depending on the cells, the sonication should be optimized to achieve the sheared chromatin fragments with the desired distribution (usually 200-500bp).
29- After the sonication, collect the liquid, and pour it into a 2-ml microtube
30- Repeat the sonication for the next 130ul.
   - Don't use a Covaris microTUBE more than 2 times.
   - Don't forget to resuspend the cells for the next run
   - Don't generate too much bubble in the sonication tube, specially in the middle of the solution (having a bubble on top is okay)
31- Pool 6x130ul in one 2-ml microtube. Now you have sheared chromatin from the original 24million cells.
32- After the sonication spin the tubes 10min at 12000g
33- Collect the supernatant, (it should be ~600-700ul)
34- You may keep 2% as the input for ChIP-Seq, also 20ul for checking the distribution of the sheared chromatin on gel.
35- Optional: you may add 10ul of RNase A to be sure the proteins are not cross-linked via RNA to DNA.
36- Add the following reagents to the sheared chromatin in each microtube:
   - Add 24 ul NaCl 5M (final conc. 150mM)
   - Add 80 ul Tritone X100 10% (final conc. 1%)
   - Add 40 ul NP40 10% (final conc. 0.5%)
   - Vortex gently to homogenize the reagents completely

- Add a proper amount of the antibody. (Normally between 1-5 ug depending on the antibody)
    i. You may include a no-antibody control, or normal IgG control
- Fill it with TE up to ~800ul
- Vortex slightly, and gently

37- Shake overnight in a Thermomixer (Eppendorf) at 4°C with 800 RPM agitation.
38- The day after that, spin the tubes at 12000g, 10min
39- Collect the supernatant (~790ul), pour it in a 2-ml microtube
40- Add 700ul IP buffer into each of the microtubes
41- Wash the magnetic Dynabeads (protein G or protein A depending on the antibody) with IP buffer
42- Add 30ul magnetic Dynabeads
43- Rotate the samples 2-4 hours at 4°C head to tail, at 20 RPM,
44- Put the tubes on the magnet, after 2min remove the liquid, and take the tubes off the magnet
45- Wash the beads by 1000ul Tris-HCl 100mM pH= 7.5 <u>(No EDTA)</u>
- Each washing consists of resuspending the beads in the washing solution by inverting/rotating the tubes (<u>Don't vortex</u>), briefly spinning, and putting on the magnet for 2 min to remove the solution

46- Resuspend the beads in 100ul TdT buffer 1x, and keep 5min at RT. <u>Don't pipet the</u> beads, just gently swirl the tubes.
47- Put the tubes on the magnet, after 1min remove the liquid, and take the tubes off the magnet
- be careful not to dry the beads

48- Resuspend the beads in 93ul TdT buffer 1x
- +5ul ddUTP-Biotin (1mM stock)
- +2ul TdT

49- Incubate 30min in a thermomixer at 37°C, with 500 RPM agitation
- The no-antibody (or IgG control) should also be treated with TdT and biotinylated nucleotide to estimate background chromatin contamination.

50- Wash the beads 6 times with 1ml ice-cold IP buffer at room temp.
51- Resuspend the beads in 100ul of the elution buffer by vortexing

52- Incubate 15min at 37, with 750 RPM agitation
53- Collect the supernatant, and discard the beads
54- If you are comparing one target with the negative control, and you have SILAC labeled proteins, then mix the content of the two tubes (2x100ul). Therefore, at this step you have 1 tube per replicate
55- Add 1300ul IP buffer
56- Add 50ul Streptavidin magnetic beads to each tube
57- Rotate 1 hour at RT
58- Put the tubes on the magnet after 2 min discard the solution.
59- Wash the beads 3 times by SDS wash buffer
    - Each washing consists of resuspending the beads in the washing solution by vortexing, briefly spinning, and putting on the magnet for 2 min to remove the solution
60- Wash once with BW2x buffer
61- Wash the beads with Iso-propanol wash buffer, 2 times
62- Wash the beads with acetonitrile wash buffer, 4 times
63- Resuspend the beads in 80ul acetonitrile wash buffer, and transfer it into a 0.2-ml PCR tubes.
64- To be sure that all the beads have been transferred, pour 80ul acetonitrile wash buffer into the 2-ml tube and transfer it to the PCR tube.
65- Put the tubes on the magnet, and remove the wash buffer.
66- Resuspend in 14ul digestion buffer
67- Add 1ul DTT 100mM,
68- Incubate 95 °C, 20min
69- Once the tube is cool enough, add 1ul IAA 200mM, 60min at RT in a dark place
70- Add 1ul DTT 100mM,
71- Remove the beads, and transfer the liquid into a new tube.
72- Add 200ng Trypsin and 50ng LysC
73- Incubate at 37°C for 14 hours

Clean up the peptides by SP3 protocol (Hughes et al., 2014)This protocol removes SDS and other detergents:

1- Prepare the SP3 beads by mixing 2x20ul of Sera-mag beads (65152105050250 and 45152105050250) in a 0.2-ml PCR tube

2- Add 160ul of deionized H2O, vortex and put the tube on the magnet
3- Discard the liquid, and resuspend the beads in 200ul of dH2O
4- vortex and put the tube on the magnet
5- repeat step3 and 4 two times more.
6- Resuspend the beads in 100ul of dH2O. The beads could stored 2 weeks in a fridge.
7- Add 2ul of the SP3 beads to the peptides to be cleaned up, and vortex
8- Add 200ul ACN 100% and vortex
9- Spin briefly
10- Add 20ul more ACN 100% to each PCR tube, and close the caps.
11- Wait 10min
12- Put the tubes on the magnet for 1-2min
13- Take the liquid but don’t discard it! It contains DNA, which can be used for qPCR or sequencing after DNA-purification.
14- Pour it into a 1.5ml microtube.
15- Without vortexing and disturbing the SP3 beads, pour 200ul of ACN 100% and wait a few seconds
16- Discard the liquid.
17- Take the tubes off, and spin briefly
18- Discard the residues of the ACN
19- Add 10ul of DMSO 2% on wall of the tube, don’t pipette
20- Vortex the tube vigorously, and spin it for a few seconds
21- Sonicate the tubes in water bath for 5min
22- Put the tubes on the magnet
23- In new PCR tubes, add 1ul of formic acid 1%
24- Take the liquid and pour it into the new PCR tubes
25- Take 10ul and pour it into the glass insert or another PCR tube to be injected to the mass spec.

Recovering DNA after digesting the proteins:

1- Dry out the ACN fraction, obtained in the step 13, using a speedvac
2- Reconstitute the DNA in 50ul of TE buffer.
3- Add 100ul of Ampure XP beads, vortex, spin, and wait 10min
4- Put the samples on a magnet, and wait a few min
5- Discard the liquid, and add 200ul of freshly prepared 70% Ethanol

6- Repeat the last step
7- Spin briefly
8- Put the samples on a magnet, and discard the residues of Ethanol
9- Resuspend the beads in 20ul of Tris-Cl 10mM without EDTA.
10- The DNA could be directly used for library preparation, or qPCR.
11- The input control could be prepared with the same protocol, however, cross-linking should be reversed and proteins should be digested by Trypsin or proteinase K, ideally in parallel to the other samples.

ChIP-MS and RIME

ChIP-MS procedure was as same as SICAP, however omitting TdT-mediated end-labeling of DNA with biotin. Therefore, following 6 washing steps by IP buffer, the proteins were reverse cross-linked by SDS-PAGE loading buffer. After running the SDS-PAGE for 2-3cm, each lane was divided into 3 pieces and the proteins were digested in-gel by trypsin (Shevchenko et al., 2006).

RIME was essentially carried out as described in the original paper (Mohammed et al., 2013), and is similar to ChIP-MS (above) with the difference that proteins are digested on-bead. The amount of the antibody and the number of cells were the same as ChIP-SICAP. After digestion and clean-up RIME samples were subjected to High pH peptide fractionation.

High pH fractionation and mass spectrometry

Following digestion of the proteins and acidification to remove RapiGest, the peptides were cleaned using stage-tipping procedure (Rappsilber et al., 2007). Then the samples were subjected to fractionation using high pH reverse-phase chromatography. Peptides were fractionated on an Agilent 1200 Infinity HPLC system with a Gemini C18 column (3 μm, 110

Å, 100 × 1.0 mm, Phenomenex) using a linear 60 min gradient from 0% to 35% (v/v) acetonitrile in 20 mM ammonium formate (pH 10) at a flow rate of 0.1 ml/min. Elution of peptides was detected with a variable wavelength UV detector set to 254 nm. Thirty-two 1-min fractions were collected that were subsequently pooled into ten fractions. Each fraction was then analyzed using LC-MS on a Orbitrap Velos Pro mass spectrometer (Thermo Fisher Scientific) connected to a nanoAcquity UPLC (Waters) via a nanoelectrospray ion source (Thermo). Peptides were separated with a BEH300C18 (75 µmx 250 mm, 1.7 µm) UPLC column (Waters) using a stepwise 60-min, from 3% to 85% (v/v) acetonitrile in 0.1% (v/v) formic acid at a flow rate of 300 nl/min. The LTQ-Orbitrap Velos Pro mass spectrometer was operated in data-dependent mode, acquiring one survey MS scan in the orbitrap followed by up to 15 fragmentation scans (TOP15) of the most abundant ions analyzed in the LTQ by CID fragmentation. Only charge states of two and higher were allowed for fragmentation. Essential MS settings were: full MS: AGC = 10E6, maximum ion time = 500 ms, m/z range = 375–1600, resolution = 30 000 FWHM; MS2: AGC = 30 000, maximum ion time = 50 ms, minimum signal threshold = 1500, dynamic exclusion time = 30 s, isolation width = 2 Da, normalized collision energy = 40, activation Q = 0.25.

Processing mass spectrometry data

The MS spectra were analyzed using Proteome Discoverer 1.4 (Thermo Fisher Scientific), and the proteins were identified using MASCOT search engine (Matrix Science) against the Mus musculus proteome of the Uniprot database. Searches were carried out based on tryptic specificity. The precursor and ms/ms tolerance were set on 20 ppm and 0.5 Da, respectively. Carbamidomethylation of Cytosine was selected as a fixed modification, and as dynamic modifications, oxidation of methionine and N-terminal acetylation were selected in addition

to the SILAC labels (Arg 10, and Lys 8). The Perculator algorithm (Kall et al., 2007) was used to limit FDR rates to a q-value<0.01. Proteins were identified and quantified by at least 1 unique peptide. For subsequent analysis, protein grouping was enabled to consider only the master proteins.

In comparison between Nanog and No-antibody control, the option for "replace the missing values with minimum quantity" in proteome discoverer was used. Because most of the proteins were over-represented in the Nanog samples. No normalization was applied in this analysis. Proteins identified with both replicates, and with Nanog over no-antibody ratios > 2-fold were considered as enriched proteins for subsequent analyses.

In comparison between 2iL and serum, the distributions of the ratios were normalized by the median. Then, the analysis was carried out without "replace the missing values with minimum quantity". For a few protein groups no ratio was reported due to the signals only from one of the channels. In these cases, the analysis was repeated using "replace the missing values with minimum quantity". Then the maximum ratio was limited to 32-fold, which was reported for these protein groups. To identify differentially expressed/bound proteins, we applied t-test using Limma package (Ritchie et al., 2015), then the p-values were adjusted using Benjamini-Hochberg method (adj. p-value). We considered adj. p-values <0.10 as significant changes.

For the analysis of post-translational modification, the acquired Raw data were analyzed by the integrated MaxQuant software v.1.5.2.8, using the Andromeda search engine (Cox, J., and Mann, M. 2008, Cox, J., et al 2009). The MOUSE 1401 database (51195 entries) was used for peptide identification. Enzyme specificity was set to trypsin, estimated peptide false discovery rate (FDR) identification to 1%, a maximum of 3 missed cleavages were permitted and the minimum peptide length was fixed to 5 amino acids.

Different MaxQuant jobs were carried out in order to include as variable modifications: mono- (+14.016 Da) and di-methylation (+28.031 Da) of lysine and arginine, acetylation of lysine (+42.010 Da), ubiquitination of lysine (+114.043 Da), phosphorylation of serine, threonine and tyrosine (+79.966 Da), and oxidation of methionine (+15.995 Da). Only peptides with evidence score higher than 60 (Cox, J., et al 2011) and localization probability score greater than 0.75 (Olsen, J. V., et al 2006, Monetti, M., et al 2011, Pines, A., et al 2011) were accepted. For each experiment, evidence SILAC ratios were normalized on the corresponding protein SILAC ratio, to define peptide trends.

ChIP-seq

Shearing chromatin and IP were performed as described above for ChIP-SICAP, however omitting the DNA-labeling. In addition, an aliquot was taken from the clear sheared chromatin to be used as an input control. After the IP and six rounds of washing steps with the ice-cold IP buffer, the beads were resuspended in TE buffer plus 1% of SDS. Then the samples heated at 95°C for 20min, and 40µg proteinase K was added for protein digestion at 55°C for 30min. Next DNA was purified using phenol/chloroform isoamyl alcohol and precipitated using glycogen and ethanol. Finally, DNA was resuspended in 30µl of Tris-HCl 10mM.

To prepare the library for Illumina sequencing, purified ChIP DNA was end-repaired by Klenow, T4 DNA polymerase and T4 polynucleotide kinase. Then DNA fragments were subjected to A-tailing, and NEBNext adapter ligation (NEB Index Primers Set 1, E7335S). Following PCR for 12 cycles, the amplicons were size-selected by mixing 50µl PCR products with 30µl of Ampure XP beads. The supernatant was collected, and again 45 µl of AmpureXP beads was added. After 2 rounds of washing with 70% ethanol, the DNA was

eluted in 50µl of Tris-HCl 10mM. Once again the eluted DNA was mixed with 48µl of AmpureXP beads, and after the washing, they were eluted by 15 µl of Tris-HCl 10mM. Sequencing was carried out by Illumina HiSeq 2000 according to the manufacturer's protocols.

ChIP-seq data analysis

Unless stated otherwise, analysis was performed in a local installation of Galaxy (Blankenberg et al., 2010; Giardine et al., 2005; Goecks et al., 2010) maintained by the EMBL Genome Biology Computational Support. The 50-bp single-end reads were aligned to build version NCBI37/MM9 of the mouse genome using Bowtie version 2(Langmead et al., 2009) using standard options (Galaxy Tool version 0.2, sensitive preset). Reads failing to be mapped or mapping at several locations (as identified by the XS tag set by bowtie2) were removed using the "Filter SAM" tool and the "Select" tool, respectively. Read duplicates where identified and removed using Picard's Mark Duplicates (http://broadinstitute.github.io/picard). Sequencing data quality was assessed using FastQC and the Deeptools package (Ramirez et al., 2014). ChIP quality was estimated by cross-correlation using the "SPP" tool as suggested by ENCODE ChIP-seq guidelines (Landt et al., 2012). Finally, reproducibility of ChIP replicates and final peak selection was achieved using the IDR pipeline depicted in Fig 7D of Landt et. al. (Landt et al., 2012), and implemented following instructions from https://sites.google.com/site/anshulkundaje/projects/idr. We used MACS version 2 (Zhang et al, 2008) as the underlying peak caller with recommended options (i.e. setting p-value cutoff to 1e-3). The final list of peaks used in the study corresponds to the "optimal" list (i.e. using peak called on merged replicates) at a 2% IDR cutoff. Heatmaps were produced using the Deeptools "compute-Matrix" and "heatmapper" tools on input-

subtracted coverage files (see below). Coverage files (bigwig format) were generated using the Deeptools “bam-Coverage” tool using the “Normalize coverage to 1x” option. Input subtraction (from ChIP signal) was performed using the Deeptools “bigwig-Compare” tool. An average fragment size of 200 bp and bin sizes of 50 bp was systematically used.

RNA-seq data analysis

The 84-bp single-end reads were aligned to build version NCBI37/MM9 of the mouse genome using STAR (Galaxy Tool RNA STAR Version 2.4.0d-2) and standard options. Count tables were generated at the gene level using htseq-count (Galaxy Tool Version 0.6.1galaxy1, with --mode union --minaqual 10 --stranded reverse and other defaults) using ENSEMBL genome annotations version 37.67 (Mus_musculus.NCBIM37.67.gtf). Finally, differentially expressed genes for the different contrasts were called with DESeq2 (Galaxy Tool DESeq2 Version 2.1.8.3).

List of the antibodies used for the IP

Nanog (D2A3) XP Rabbit mAb (Cell Signaling Tech, 8822 S) was applied 1:100 (v/v) to the sheared chromatin obtained from 24 million cells for the ChIP-SICAP assay.

Oct-4A (C30A3C1) Rabbit mAb (Cell Signaling Tech, 5677 S) was applied 1:50 (v/v) to the sheared chromatin obtained from 24 million cells for the ChIP-SICAP assay.

Human SOX2 Affinity Purified Polyclonal Ab (R&D Systems, AF2018) 25µg was applied to the sheared chromatin obtained from 24 million cells the ChIP-SICAP assay.

E-Cadherin (24E10) Rabbit mAb (Cell Signaling Tech, 3195s) was applied 1:50 (v/v) to the sheared chromatin obtained from 24 million cells for the ChIP-SICAP assay.

Trim24/TIF1a antibody (Bethyl lab, A300-815A) 2.5 μg was applied to the sheared chromatin obtained from 24 million cells for the ChIP-Seq assay.

GO analysis and Cytoscape plugins

Annotations of the genes were determined either using Perseus software (Cox and Mann, 2008), or were downloaded directly from Uniprot database. GO biological processes and their enrichments were determined using DAVID bioinformatics resources (Huang da et al., 2009). Cytoscape (Shannon et al., 2003) version 3.2.1 was used to apply enhancedGraphics (Morris et al., 2014).

Transduction of MEF cells with Trim24, iPS generation and microscopy

Trim24 cDNA was amplified by PCR using the Forward primer: 5'-AATGGAGGTGGCTGTGGAGAA-3' and the reverse primer: 5'-tttggatCCAGTGCGGCGTTACTTAA-3'. The reverse primer contained the BamHI site. The PCR was carried out by Phusion polymerase to obtain blunt ends. Then the products were digested by BamHI, and the 5'-ends were phosphorylated by polynucleotide kinase (PNK). FU-tetO-hcmyc (Addgene plasmid # 19775) was digested by XbaI, and fill in by Klenow to make one end blunt. Then the vector was digested by BamHI, followed by dephosphorylation of the 5'-end by CIP and ligation to the vector. The correct coding-sequencing was confirmed by sanger sequencing. Subsequently HEK293T cells were transfected by pFU-tetO-Trim24, psPAX2, and VSV-G as described previously (Hansson et al., 2012). Briefly 8 million cells

were seeded in a T75 flask, after an overnight incubation the cells were transfected using FuGENE HD. After 8 hours the media of the cells was changed with fresh media. About 2 days after the transfection, the virus-containing media was collected, filtered, and concentrated using Amicon100kD ultrafiltration tubes. The concentrated media was used to infect 3 wells in a 6-well plate containing ~30000 reprogrammable MEFs (Stadtfeld et al., 2010) in each well. One day after the infection, the viral media was discarded and fresh media containing ~100000 feeder MEF cells were added to the wells. One day later, the cells were fed with ES media plus doxycycline (1 µg/ml) for the next two weeks. Then the cells were fixed by formaldehyde 4%, and ~1300 images were automatically taken from each well in EGFP channel to cover almost all surface of the wells (Figure S5). The images were stitched by grid collection stitching package (Preibisch et al., 2009) incorporated in Fiji. Then the number of Oct4-EGFP positive colonies were counted by “Analyze particle” in Fiji with minimum area of the colonies was set to be 250 (Table S6).

Transduction of the ES cells with shTrim24 and shP53

The knock down (KD) was carried out by validated Sigma-Alderich lentiviral vectors: non-targeting shRNA, shTrim24 (TRCN0000088518), shTrp53 (TRCN0000310844), and the mixture of the last two shRNAs. The lentiviruses were produced and concentrated as mentioned above. For each knockdown, 3 independent transductions were performed. The ES cells adapted in 2iL medium were infected with the viruses, and after 24 hours the viral media was replaced with the regular 2iL medium. Then 24 hours later, the cells were lysed, and RNA was extracted to be analyzed by qPCR and mRNA-seq. The RNA extraction and library preparation from poly(A)-RNA were carried out according to the standard Illumina TruSeq protocol.

Cell culture

46c mouse ES cells were grown feeder-free on 0.2% gelatinized cell culture plates in either traditional ES media with serum or 2iL-media. The serum media contained DMEM high glucose (Life technologies, 11965-092) supplemented with 15% fetal bovine serum (Life technologies, 10270-106), 100µM MEM non-essential aminoacids (Life technologies, 11140-050), 1x Glutamax (Life technologies, 35050-061), 1x penicillin and streptomycin (Life technologies, 15140-122), 100µM of 2-mercaptoethanol (Sigma, M7522), and 200ng/ml of LIF (EMBL, protein expression core facility). The 2i+LIF (2iL)-media contained DMEM/F12 media for SILAC (Pierce, 88215), 100µM MEM non-essential aminoacids (Life technologies, 11140-050), 1x Glutamax (Life technologies, 35050-061), 1x penicillin and streptomycin (Life technologies, 15140-122), 100µM of 2-mercaptoethanol (Sigma, M7522), 0.5mg/ml of BSA (Sigma, A3059), 200ng/ml of LIF (EMBL, protein expression core facility), 1µM of PD0325901 (Reagents Direct, 39-C68), 3µM of CHIR99021 (Reagents Direct, 27-H76). In addition, for light SILAC, 100 mg/ml of Lysine (L8662), 100 mg/ml of Arginine (Sigma, A6969) and 100 mg/ml of Proline (Sigma, P5607) were added to the 2i-media. For Heavy SILAC, 100 mg/ml of $^{13}C_6$,$^{15}N_2$-L-Lysine HCl (Silantes, 211604102), 100 mg/ml of $^{13}C_6$,$^{15}N_4$-L-Arginine HCl (Silantes, 201604102) and 100 mg/ml of Proline (Sigma, P5607) were added to the 2iL-media.

References

Blankenberg, D., Von Kuster, G., Coraor, N., Ananda, G., Lazarus, R., Mangan, M., Nekrutenko, A., and Taylor, J. (2010). Galaxy: a web-based genome analysis tool for experimentalists. Current protocols in molecular biology / edited by Frederick M Ausubel [et al] *Chapter 19*, Unit 19 10 11-21.

Cox, J., and Mann, M. (2008). MaxQuant enables high peptide identification rates, individualized p.p.b.-range mass accuracies and proteome-wide protein quantification. Nature biotechnology *26*, 1367-1372.

Giardine, B., Riemer, C., Hardison, R.C., Burhans, R., Elnitski, L., Shah, P., Zhang, Y., Blankenberg, D., Albert, I., Taylor, J*., et al.* (2005). Galaxy: a platform for interactive large-scale genome analysis. Genome research *15*, 1451-1455.

Goecks, J., Nekrutenko, A., Taylor, J., and Galaxy, T. (2010). Galaxy: a comprehensive approach for supporting accessible, reproducible, and transparent computational research in the life sciences. Genome biology *11*, R86.

Hansson, J., Rafiee, M.R., Reiland, S., Polo, J.M., Gehring, J., Okawa, S., Huber, W., Hochedlinger, K., and Krijgsveld, J. (2012). Highly coordinated proteome dynamics during reprogramming of somatic cells to pluripotency. Cell Rep *2*, 1579-1592.

Huang da, W., Sherman, B.T., and Lempicki, R.A. (2009). Systematic and integrative analysis of large gene lists using DAVID bioinformatics resources. Nature protocols *4*, 44-57.

Hughes, C.S., Foehr, S., Garfield, D.A., Furlong, E.E., Steinmetz, L.M., and Krijgsveld, J. (2014). Ultrasensitive proteome analysis using paramagnetic bead technology. Mol Syst Biol *10*, 757.

Kall, L., Canterbury, J.D., Weston, J., Noble, W.S., and MacCoss, M.J. (2007). Semi-supervised learning for peptide identification from shotgun proteomics datasets. Nature methods *4*, 923-925.

Landt, S.G., Marinov, G.K., Kundaje, A., Kheradpour, P., Pauli, F., Batzoglou, S., Bernstein, B.E., Bickel, P., Brown, J.B., Cayting, P*., et al.* (2012). ChIP-seq guidelines and practices of the ENCODE and modENCODE consortia. Genome research *22*, 1813-1831.

Langmead, B., Trapnell, C., Pop, M., and Salzberg, S.L. (2009). Ultrafast and memory-efficient alignment of short DNA sequences to the human genome. Genome biology *10*, R25.

Li, M., He, Y., Dubois, W., Wu, X., Shi, J., and Huang, J. (2012). Distinct regulatory mechanisms and functions for p53-activated and p53-repressed DNA damage response genes in embryonic stem cells. Mol Cell *46*, 30-42.

Mohammed, H., D'Santos, C., Serandour, A.A., Ali, H.R., Brown, G.D., Atkins, A., Rueda, O.M., Holmes, K.A., Theodorou, V., Robinson, J.L*., et al.* (2013). Endogenous purification reveals GREB1 as a key estrogen receptor regulatory factor. Cell reports *3*, 342-349. Montojo, J., Zuberi, K., Rodriguez, H., Kazi, F., Wright, G., Donaldson, S.L., Morris, Q., and Bader, G.D. (2010). GeneMANIA Cytoscape plugin: fast gene function predictions on the desktop. Bioinformatics *26*, 2927-2928.

Morris, J.H., Kuchinsky, A., Ferrin, T.E., and Pico, A.R. (2014). enhancedGraphics: a Cytoscape app for enhanced node graphics. F1000Res *3*, 147.

Preibisch, S., Saalfeld, S., and Tomancak, P. (2009). Globally optimal stitching of tiled 3D microscopic image acquisitions. Bioinformatics *25*, 1463-1465.

Ramirez, F., Dundar, F., Diehl, S., Gruning, B.A., and Manke, T. (2014). deepTools: a flexible platform for exploring deep-sequencing data. Nucleic acids research *42*, W187-191.

Rappsilber, J., Mann, M., and Ishihama, Y. (2007). Protocol for micro-purification, enrichment, pre-fractionation and storage of peptides for proteomics using StageTips. Nature protocols *2*, 1896-1906.

Ritchie, M.E., Phipson, B., Wu, D., Hu, Y., Law, C.W., Shi, W., and Smyth, G.K. (2015). limma powers differential expression analyses for RNA-sequencing and microarray studies. Nucleic Acids Res *43*, e47.
Shannon, P., Markiel, A., Ozier, O., Baliga, N.S., Wang, J.T., Ramage, D., Amin, N., Schwikowski, B., and Ideker, T. (2003). Cytoscape: a software environment for integrated models of biomolecular interaction networks. Genome research *13*, 2498-2504.
Shevchenko, A., Tomas, H., Havlis, J., Olsen, J.V., and Mann, M. (2006). In-gel digestion for mass spectrometric characterization of proteins and proteomes. Nature protocols *1*, 2856-2860.
Stadtfeld, M., Maherali, N., Borkent, M., and Hochedlinger, K. (2010). A reprogrammable mouse strain from gene-targeted embryonic stem cells. Nature methods *7*, 53-55.
