# Supplementary material for: Expanding the Circuitry of Pluripotency by Selective Isolation of Chromatin-Associated Proteins
Source: Mol Cell. 2016 Nov 3;64(3):624–35. doi: 10.1016/j.molcel.2016.09.019 (PMC5101186; doi:10.1016/j.molcel.2016.09.019)
Supplement: Document S2. Article plus Supplemental Information [file mmc8.pdf]

Technology

Molecular Cell

Expanding the Circuitry of Pluripotency by Selective Isolation of Chromatin-Associated Proteins

Graphical Abstract

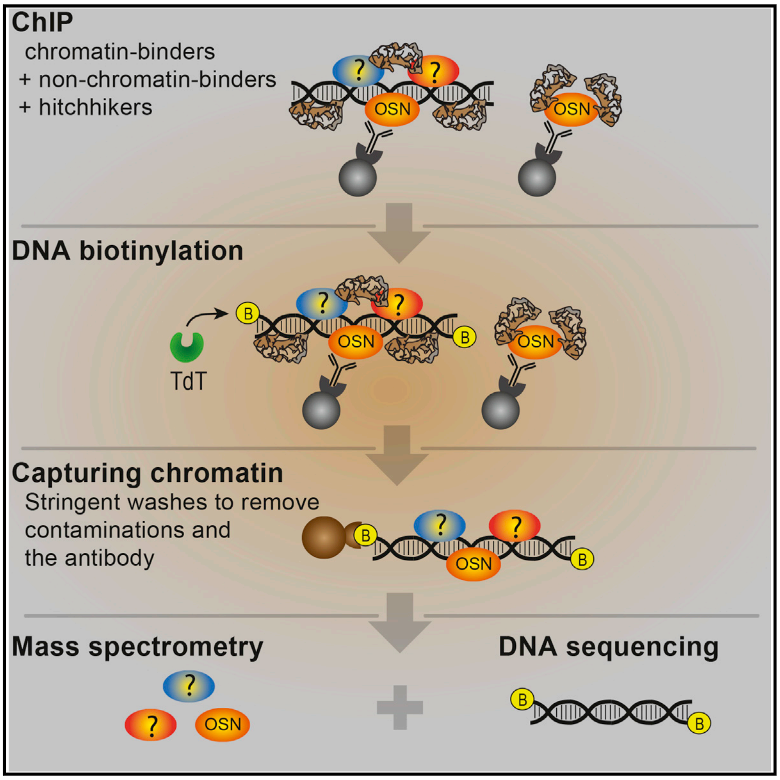

Authors

Mahmoud-Reza Rafiee,
Charles Girardot,
Gianluca Sigismondo,
Jeroen Krijgsveld

Correspondence

j.krijgsveld@dkfz.de

In Brief

Rafiee et al. introduce ChIP-SICAP as a novel method to identify chromatin-bound proteins that colocalize with a bait protein on DNA. Applied to Oct4, Sox2, and Nanog in ESCs, they identify Trim24 as a novel protein that is functionally involved in the pluripotency network by promoting cellular reprogramming.

Highlights

- ChIP-SICAP isolates and identifies proteins that colocalize on chromatin
- Chromatin composition around Oct4, Sox2, and Nanog depends on the pluripotent state
- Trim24 is part of the pluripotency network and promotes reprogramming
- ChIP-SICAP allows recovery of DNA for sequencing with ChIP-seq quality

Accession Numbers

PXD003798
E-MTAB-3802
E-MTAB-4893

Rafiee et al., 2016, Molecular Cell *64*, 624–635
November 3, 2016 © 2016 The Authors. Published by Elsevier Inc.
http://dx.doi.org/10.1016/j.molcel.2016.09.019

Molecular Cell
Technology

Expanding the Circuitry of Pluripotency by Selective Isolation of Chromatin-Associated Proteins

Mahmoud-Reza Rafiee,[1,2,3] Charles Girardot,[3] Gianluca Sigismondo,[1,2] and Jeroen Krijgsveld[1,2,3,4,*]
[1]German Cancer Research Center (DKFZ), Im Neuenheimer Feld 581, 69120 Heidelberg, Germany
[2]Excellence Cluster CellNetworks, Heidelberg University, 69120 Heidelberg, Germany
[3]European Molecular Biology Laboratory (EMBL), Genome Biology Unit, Meyerhofstrasse 1, 69117 Heidelberg, Germany
[4]Lead Contact
*Correspondence: j.krijgsveld@dkfz.de
http://dx.doi.org/10.1016/j.molcel.2016.09.019

SUMMARY

Maintenance of pluripotency is regulated by a network of transcription factors coordinated by Oct4, Sox2, and Nanog (OSN), yet a systematic investigation of the composition and dynamics of the OSN protein network specifically on chromatin is still missing. Here we have developed a method combining ChIP with selective isolation of chromatin-associated proteins (SICAP) followed by mass spectrometry to identify chromatin-bound partners of a protein of interest. ChIP-SICAP in mouse embryonic stem cells (ESCs) identified over 400 proteins associating with OSN, including several whose interaction depends on the pluripotent state. Trim24, a previously unrecognized protein in the network, converges with OSN on multiple enhancers and suppresses the expression of developmental genes while activating cell cycle genes. Consistently, Trim24 significantly improved efficiency of cellular reprogramming, demonstrating its direct functionality in establishing pluripotency. Collectively, ChIP-SICAP provides a powerful tool to decode chromatin protein composition, further enhanced by its integrative capacity to perform ChIP-seq.

INTRODUCTION

In ESCs, the three master transcription factors Oct4, Sox2, and Nanog constitute the core transcriptional circuitry (Boyer et al., 2005; Loh et al., 2006), which on the one hand promotes the expression of pluripotency genes, while on the other hand suppresses lineage commitment and differentiation (Boyer et al., 2006; Laugesen and Helin, 2014; Lee et al., 2006). In mouse ESCs, pluripotency can be further reinforced by replacing serum in conventional culture medium with two kinase inhibitors (2i), PD0325901 (inhibiting mitogen-activated protein kinase, Mek) and CHIR99021 (inhibiting glycogen synthase kinase-3, Gsk3), driving the ESCs into a condition resembling the preimplantation epiblast (Nichols and Smith, 2009; Ying et al., 2008). Hence, cells grown in 2i medium are considered as an in vitro representation of the ground state of pluripotency.

Transcriptome analysis indicated that most of the pluripotency-associated transcription factors did not change significantly in expression level between serum and 2i conditions (Marks et al., 2012), suggesting that additional proteins may sustain the functionality of core pluripotency factors in 2i. Since transcription factors, including pluripotency TFs, execute their function in chromatin, we aimed to identify proteins that associate with OSN in their DNA-bound state as opposed to interactions that may occur in soluble form. Despite the large diversity of available methods to identify protein interactions (reviewed by Dunham et al., 2012), very few of them differentiate between interactions that depend on the subcellular location. This is a critical shortcoming, especially for proteins that dynamically change location, either between or within organelles (e.g., nucleosol or chromatin bound). Indeed, transcription factors have been shown to form different complexes on and off chromatin, as demonstrated for several FOX proteins (Li et al., 2015). To specifically identify proteins in their DNA-bound state, we therefore developed a method for the *s*elective *i*solation of *c*hromatin-*a*ssociated *p*roteins (SICAP). SICAP captures an endogenous protein under ChIP conditions and then biotinylates DNA, allowing the specific isolation of DNA-bound proteins on streptavidin beads, followed by mass spectrometric protein identification. Thus, by design, ChIP-SICAP identifies chromatin-bound proteins in the direct vicinity of the bait protein on a short stretch of DNA (between 200 and 500 bp). Here we introduce and evaluate ChIP-SICAP and apply it characterize the chromatin-bound network around Oct4, Sox2, and Nanog in mouse ESCs. We demonstrate the power of ChIP-SICAP by the discovery of Trim24 as a component of the pluripotency network.

DESIGN

Many studies have been devoted to defining interactomes of pluripotency factors (Huang and Wang, 2014), most of which are based on coimmunoprecipitation (coIP) of Flag- or HA-tagged TFs, such as for Oct4 (Pardo et al., 2010; van den Berg et al., 2010), Sox2 (Lai et al., 2012; Mallanna et al., 2010), and Nanog

This is an open access article under the CC BY license (http://creativecommons.org/licenses/by/4.0/).

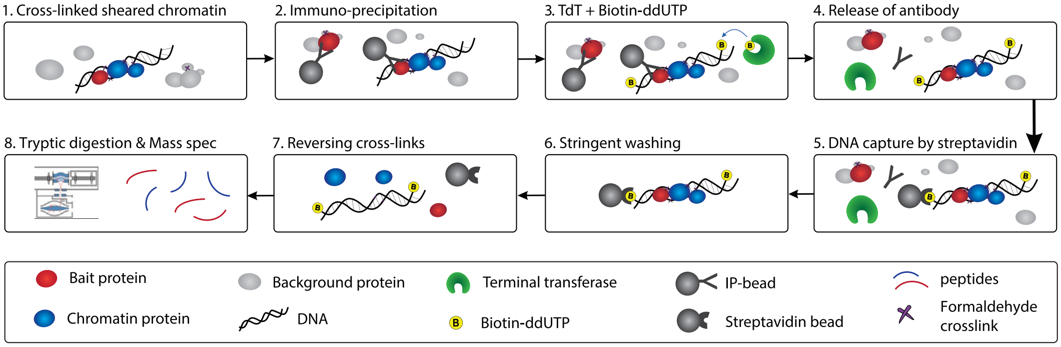

Figure 1. Principles of ChIP-SICAP

Similar to a ChIP experiment, DNA proteins are crosslinked by formaldehyde, and fixed chromatin is sheared to small fragments by sonication (1). Following immunoprecipitation with a suitable antibody (2), DNA is biotinylated by TdT and biotin-ddUTP (3). The antibody is denatured by SDS (4), and chromatin is retrieved along with interacting proteins on streptavidin beads (5). Following extensive washing (6), isolated chromatin fragments are heated to reverse the crosslinks (7). Finally, proteins are digested and identified by mass spectrometry (8).

(Gagliardi et al., 2013). The general limitation of these approaches is their need to introduce an affinity tag, often using an exogenous expression system. Studying protein interaction in the context of chromatin adds a number of other challenges, especially since chromatin is highly insoluble. To promote solubilization of chromatin, DNA can be fragmented, e.g., as carried out by sonication in ChIP protocols, combined with crosslinking to maintain protein-DNA interactions. Hence, different variations of ChIP protocols have been developed to study protein interactions on chromatin, including modified ChIP (mChIP; Lambert et al., 2009), ChIP-MS (Engelen et al., 2015; Ji et al., 2015), and rapid immunoprecipitation mass spectrometry of endogenous proteins (RIME) (Mohammed et al., 2016). ChIP-MS and RIME both apply mass spectrometric analysis on proteins immunoprecipitated from formaldehyde-crosslinked cells, but they differ in the fact that they digest proteins directly on the protein A beads (RIME) or after elution (ChIP-MS). Yet, a number of issues limit the practical utility of these methods to specifically enrich for chromatin-bound proteins. First, they often suffer from the copurification of contaminating proteins that have been referred to as "hitchhikers" (Ohta et al., 2010) to indicate their avid binding to the highly charged backbone of DNA, and other contaminants that are commonly observed in affinity-purification experiments (e.g., ribosomal proteins, hnRNPs), as documented in the CRAPome (Mellacheruvu et al., 2013). Another often-marginalized problem is that the antibody used for affinity purification represents a huge contamination in subsequent mass spectrometry, thereby masking lower abundance proteins. Finally, and maybe most importantly, none of the presented methods discriminate protein interactions occurring on and off chromatin.

BioTAP-XL (Zee et al., 2016) and a method coined as "chromatin-interaction protein MS," confusingly also abbreviated as ChIP-MS (Wang et al., 2013), tag a given protein with protein A or a His tag along with a biotin-acceptor sequence. Although this allows for stringent washing after capture on streptavidin beads, introduction of the tag may alter the functionality or expression level of the protein, while requiring a cloning step that may not be suitable or desirable for all cell types.

Because of these limitations in available approaches, we here introduce a method termed "selective isolation of chromatin associated proteins" (SICAP), which we combine with ChIP (ChIP-SICAP) to specifically purify, identify, and quantify the protein network around a chromatin-bound protein of interest (Figure 1). ChIP-SICAP combines the advantages of the aforementioned methods while bypassing their limitations, in that it targets endogenous proteins, does not require protein tagging or overexpression, uses formaldehyde for chromatin crosslinking, and allows very stringent washing, including removal of the antibody. Furthermore, ChIP-SICAP uniquely benefits from the double purification of protein-DNA complexes, accomplished by subsequent ChIP of the protein of interest, and an innovative step to biotinylate DNA allowing capture and stringent washing of the protein-DNA complex.

ChIP-SICAP (Figure 1) starts from crosslinked and sheared chromatin using established ChIP procedures (Nelson et al., 2006), followed by addition of a suitable antibody and capture of the protein-DNA complex on protein A beads. The key step of ChIP-SICAP is then the end labeling of DNA fragments with biotin by terminal deoxynucleotidyl transferase (TdT) in the presence of biotinylated nucleotides. TdT is a template-independent DNA-polymerase-extending DNA 3′ end regardless of the complementary strand, which is also used in the so-called TUNEL assay to detect double-stranded DNA breaks in apoptotic cells (Jones and Dive, 1999). Next, addition of ionic detergents (7.5% SDS) and a reducing agent disassembles all protein interactions (except those crosslinked to DNA), denatures the antibody, and releases chromatin fragments. Biotinylated DNA-protein complexes are then captured on streptavidin beads, followed by a number of stringent washes (subsequently with 1% SDS, 2M NaCl, 20% isopropanol, and 40% acetonitrile) to effectively remove contaminating proteins and the IP antibody. Finally, protein-DNA crosslinks are reversed by heating, and proteins are proteolytically digested for MS-based identification (Figure 1). As a result, ChIP-SICAP identifies the proteins that colocalize with the bait on a short fragment of chromatin.

RESULTS

End Labeling of DNA Significantly Improves Purification of Chromatin-Associated Proteins

To evaluate the performance of ChIP-SICAP, we targeted Nanog as the bait protein in mouse ESCs and performed a comparative analysis with a no-antibody control (noAB) using differential SILAC labeling. In two independent ChIP-SICAP assays, we reproducibly identified 634 proteins, of which 567 were enriched in comparison to the negative control (Nanog/noAB >2-fold in both replicates; Figure S2 and Table S1). Reassuringly, ranking the enriched proteins by their estimated abundance (based on MS peak area) revealed histones and Nanog itself as the most abundant proteins (Figure 2A). This indicates the clear enrichment of chromatin and confirms the specificity of the used antibody. In addition, Oct4 and Sox2, two well-known Nanog interactants, were also among the top-enriched proteins. Proteins of lower intensity include many other known interaction partners of Nanog, as well as potential novel candidates (further discussed below). We then evaluated the benefit of DNA-biotinylation by repeating the same experiment, but omitting the TdT-mediated end labeling of DNA, in two slightly different procedures using protocols as described for RIME (Mohammed et al., 2016) and ChIP-MS (Engelen et al., 2015; Ji et al., 2015). Under ChIP-MS conditions, we identified 981 enriched proteins (out of 1,044 detected with both replicates), i.e., twice the number obtained from ChIP-SICAP (Figures 2A and S2). Using RIME (Figure S1), i.e., digesting proteins on-bead rather than after reversal of crosslinking, we identified 1,232 enriched proteins (out of 1,609 detected by both replicates). Apart from this even further increased number of proteins, ribonucleoproteins (RNPs) now outcompeted histones as the most abundant proteins (Figure 2A). In ChIP-MS, Nanog was identified only in one replicate, while both in ChIP-MS and RIME Oct4 and Sox2 ranked much lower compared to ChIP-SICAP (Figure 2A), possibly as the result of copurification of contaminant proteins.

We next performed a rigorous analysis on these datasets to assess the performance and specificity of the three methods to enrich for chromatin-bound proteins. First, a Gene Ontology (GO) analysis revealed RNA processing and translation as the top-enriched biological processes (BPs) in the ChIP-MS and RIME (Figure S2C), reflecting the presence of many ribosomal proteins, hnRNPs, and splicing factors (Tables S1F and S1G). These proteins are often observed to copurify nonspecifically in affinity-purification procedures, and indeed they feature prominently in the CRAPome database (Mellacheruvu et al., 2013). This suggests that these are contaminant proteins not likely to be related to the Nanog network, although we cannot exclude that some individual RBPs can associate with chromatin. In contrast, processes related to chromatin and transcription are enriched in the ChIP-SICAP dataset (Figure S2C,), while RNA processing ranked only 17th (Table S1E). This indicates that ChIP-SICAP more specifically enriches for proteins that reflect the known function of Nanog in transcriptional regulation.

We next evaluated the presence of 278 proteins previously reported to interact with Nanog in multiple coIP studies, collected in BioGrid. Of these, 109 were identified by ChIP-SICAP (out of 567 detected proteins, 19%) (Figure S2F; Table S1H), compared to 132 proteins (13.5% of the 981 detected proteins) and 156 proteins (12.5% of the 1,232 detected proteins) in ChIP-MS and RIME, respectively. Although ChIP-SICAP recovers fewer known Nanog interactants, their proportion among the detected proteins is much higher, suggesting a higher precision of ChIP-SICAP over ChIP-MS and RIME. Although ideally both the absolute and relative number of returned true positives should be maximized in interactome analyses, specificity seems of greater practical utility. An extreme example is our total ESC proteome dataset containing, among 6,500 proteins, 232 Nanog interactants, i.e., with a specificity of 3.5%.

To further compare the performance of each method, we included protein abundance (estimated from MS intensity) as an additional parameter, allowing us to weigh proteins by relative enrichment within each dataset rather than treating all of them equally. Specifically, we summed the MS intensities of Nanog interactome (as defined in BioGRID) and other chromatin/DNA-binding proteins as potential true positives (PTPs). This was normalized for the total protein intensity of the same sample to estimate the relative abundance of PTPs for each method. Similarly, we calculated the ratios for ribosomal proteins and other components of RNA processing (Figure 2B; Tables S1A–S1C) as well as cytoplasmic proteins as representatives of potential false positives (PFPs). In doing so, 27% of protein intensity in ChIP-SICAP is represented by Nanog-interacting proteins, more than in any of the other datasets (Figure 2B). In addition, other chromatin-binding proteins add another 57% of intensity, collectively accounting for 85% of the total amount of protein recovered by ChIP-SICAP, compared to 47% and 55% in ChIP-MS and RIME, respectively (Figure 2B). Conversely, ChIP-SICAP better removes common contaminants and other cytoplasmic proteins, accounting for 7% of the protein intensity, compared to 29% and 33% in RIME and ChIP-MS, respectively. Taking the intensity ratio of PTPs and PFPs as a proxy for the specificity of each method, ChIP-SICAP (ratio 13.6) scored significantly better than RIME (1.9) and ChIP-MS (1.4) or the total proteome (0.6) as an example of a nonselective method (Figure 2B). Furthermore, stringent washing procedures in ChIP-SICAP resulted in the detection of far fewer peptides originating from IgG (used for IP of Nanog) and protein A (used for capture of the immunoprecipitated complex) (Figure 2C), resulting in an overall reduction of these contaminating proteins between 10- and 10,000-fold in ChIP-SICAP compared to RIME and ChIP-MS (Figure 2C).

We next tested to what extent the various protein classes were enriched or depleted not only as a group (Figure 2B) but also as individual proteins. We therefore ranked all proteins in each of the four datasets by abundance, showing that, in ChIP-SICAP, known Nanog interactors, histones, and other chromatin binding proteins accumulate faster among the top-ranked proteins compared to all three other datasets (Figure 2D). Conversely, common contaminants are largely depleted from the top 100 proteins and only appear among the less abundant proteins. This is in contrast to ChIP-MS, where copurifying ribosomal proteins rank as high as in a total proteome analysis, and to RIME, which seems particularly sensitive to contamination by ribonucleoproteins (Figure 2D).

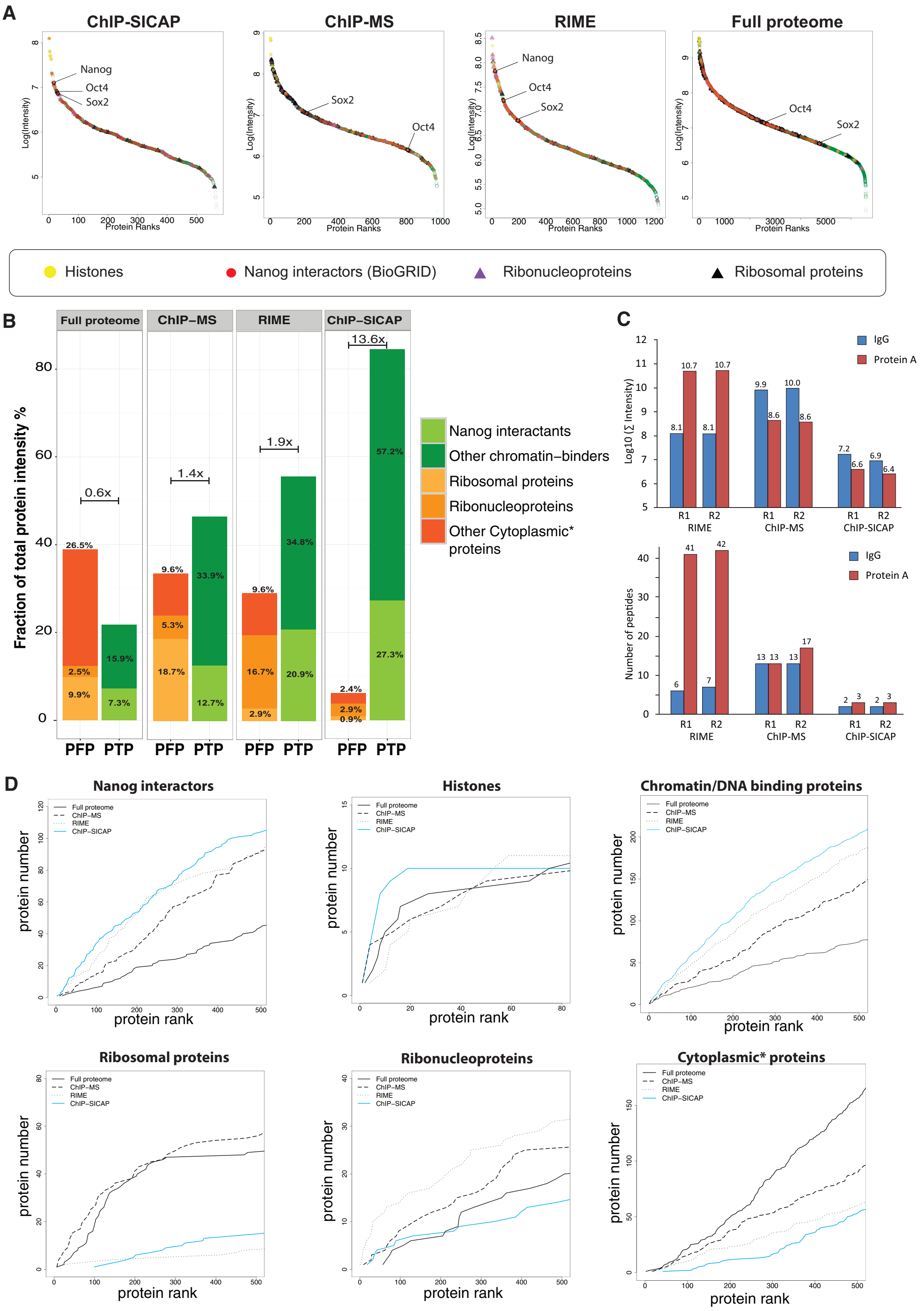

(legend on next page)

Collectively, our data show that ChIP-SICAP surpasses ChIP-MS and RIME to more specifically enrich for chromatin-bound partners of a bait protein while more effectively removing common contaminants (Figure S1).

ChIP-SICAP Reveals Chromatin Proteins that Differentially Interact with the Core Circuitry of Pluripotency

To more systematically study the composition and dynamics of proteins associated with OSN, we separately carried out ChIP-SICAP for Oct4, Sox2, and Nanog in ESCs grown in serum (light SILAC) and 2i plus LIF (2iL) medium (heavy SILAC). In ChIP-SICAP against Nanog, we detected 666 proteins, of which 296 were significantly different between the 2iL and serum conditions (t test adjusted p value $\leq$ 0.1) (Figure 3A; Tables S2A–S2C). β-catenin was detected among the most enriched proteins in 2iL condition (>20-fold increase), which is expected because of the inhibition of Gsk3β by CHIR99021 resulting in activation of Wnt signaling and translocation of β-catenin to the nucleus. Other stem cell maintenance factors that preferentially associate with Nanog in 2iL-medium included Esrrb, Klf4, Prdm14, Rex1 (Zfp42), Sall4, Tcf3 (Tcf7l1), Tbx3, Stat3, Smarca4 (Brg1), Tfap2c, and Tfcp2l (Figure 3B). Interestingly, all core-nucleosomal histones interacted less with Nanog in 2iL condition (Figure 3A), suggesting that DNA is more accessible for Nanog in the ground state and suggesting that ChIP-SICAP may also inform on global chromatin structure. This is in line with a recent study (Novo et al., 2016) showing that Nanog can remodel heterochromatin to an open architecture in a manner that is decoupled from its role in regulating the pluripotent state.

Finally, Nanog-bound loci are co-occupied with proteins maintaining DNA methylation (Dnmt3a, Dnmt3l, and Uhrf1) preferentially under serum conditions (Figure 3B), fitting with the model of higher CpG methylation rate in this cellular state.

Performing ChIP-SICAP for Oct4 and Sox2 produced results similar to that of Nanog, but with subtle yet important differences (Figures 3A and 3B; Tables S2A–S2C). In each experiment, all three master TFs—Oct4, Sox2, and Nanog—were identified, thus confirming their tight interconnection. Additionally, many stem cell maintenance factors such as β-catenin, Esrrb, Klf5, Mybl2, Prdm14, Rex1, Sall4, Tcf3, Tbx3, Stat3, and Smarca4 (Brg1), were similarly enriched in 2iL conditions in all three ChIP-SICAP assays, or in serum condition such as Uhrf1 and Dnmt3a (Figures 3A and 3B). In contrast to Nanog ChIP-SICAP, most of the nucleosome components did not show significant changes in Oct4 and Sox2 ChIP-SICAP, with the exception of macroH2A1 and macroH2A2, which preferentially associate with Oct4 (Figures 3A and 3B). The different pattern for these transcriptionally suppressive H2A variants (Buschbeck et al., 2009; Gamble et al., 2010) suggests that in 2iL condition some of the Oct4 targets may be transcriptionally repressed by recruiting macroH2A.

ChIP-SICAP Reveals Bait-Specific Interactions

We identified 407 proteins in the overlap among the three OSN ChIP-SICAP experiments (Figure 3C), 365 of which (90%) are known to have a chromatin-related function (Figure 3D), indicating that indeed we retrieved the desired class of proteins. To assess the specificity of ChIP-SICAP, and to rule out that the observed proteins were enriched irrespective of the used antibody, we used E-cadherin (Cdh1) as an unrelated bait protein to perform ChIP-SICAP. Although Cdh1 is classically known as plasma membrane protein, its cleavage by α-secretase, γ-secretase, or caspase-3 releases specific C-terminal fragments (CTFs) that translocate to the nucleus and bind to chromatin (Ferber et al., 2008). Following expectations, histones and Cdh1 were the most prominent proteins identified in Cdh1 ChIP-SICAP (Figure S3). In addition, and according to expectation, Cdh1 was identified exclusively by peptides originating from the most C-terminal CTF, along with known nuclear Cdh1 interaction partners β-catenin and δ-catenin (p120) (Ferber et al., 2008). In contrast, the stem cell maintenance factors found in OSN ChIP-SICAP were not identified (Table S2E). Collectively, this demonstrates that ChIP-SICAP reveals target-specific protein-DNA interactions.

ChIP-SICAP Reveals Changes in Chromatin Proteins and PTMs

To investigate whether changes observed in chromatin interactions around OSN were dependent on global protein expression level, we performed a total proteome comparison of ESCs grown in 2iL and serum conditions. Interestingly, protein ratios did not always correlate between in ChIP-SICAP and total proteome. For instance, β-catenin preferentially binds to OSN sites in 2iL versus serum (32-fold higher based on Oct4 and Nanog, and ~3.3-fold based on Sox2), without a change in overall expression (Tables S2A–S2D; Figure S3C). We observed a similar trend for Esrrb, Kdm3a, Mybl2, Tcf7l1 (Tcf3), Tle3, Sall4, Scml2, Smarcd2, Smarce1, Stat3, Trim24, and Zfp42 (Tables S2A–S2D; Figure S3C). This suggests that alternative mechanisms are in place to induce interaction with chromatin in general, and with the OSN network in particular. Intrigued by the differential chromatin-binding proteins, we analyzed the OSN ChIP-SICAP data for the presence of proteins modified by phosphorylation, acetylation, methylation, and ubiquitination. Indeed, we identified 95 ChIP-SICAP proteins carrying one or more of these modifications (Table S2E). Phosphorylation was the most frequent modification, observed on 84 sites (Figure S3B;

Figure 2. Comparing Performance of ChIP-SICAP to ChIP-MS and RIME Using Nanog as the Bait

(A) Enriched proteins ranked by abundance.

(B) Relative abundance of various protein classes. Stacked bars show the abundance of Nanog interactors (light green), other chromatin/DNA binding proteins (dark green), ribosomal proteins (amber), ribonucleoproteins (orange), and other cytoplasmic proteins (burnt orange) relative to the total protein abundance within each method. PTP, potential true positive. PFP, potential false positive.

(C) Total MS intensity (top panel) and number of peptides (bottom panel) produced from antibody and protein A contamination.

(D) Cumulative distribution of abundance-ranked proteins within the four datasets for various protein classes. Asterisk indicates cytoplasmic proteins that are neither Nanog interactor nor chromatin/DNA binder nor ribosomal nor ribonucleoprotein. See also Figure S2 and Table S1.

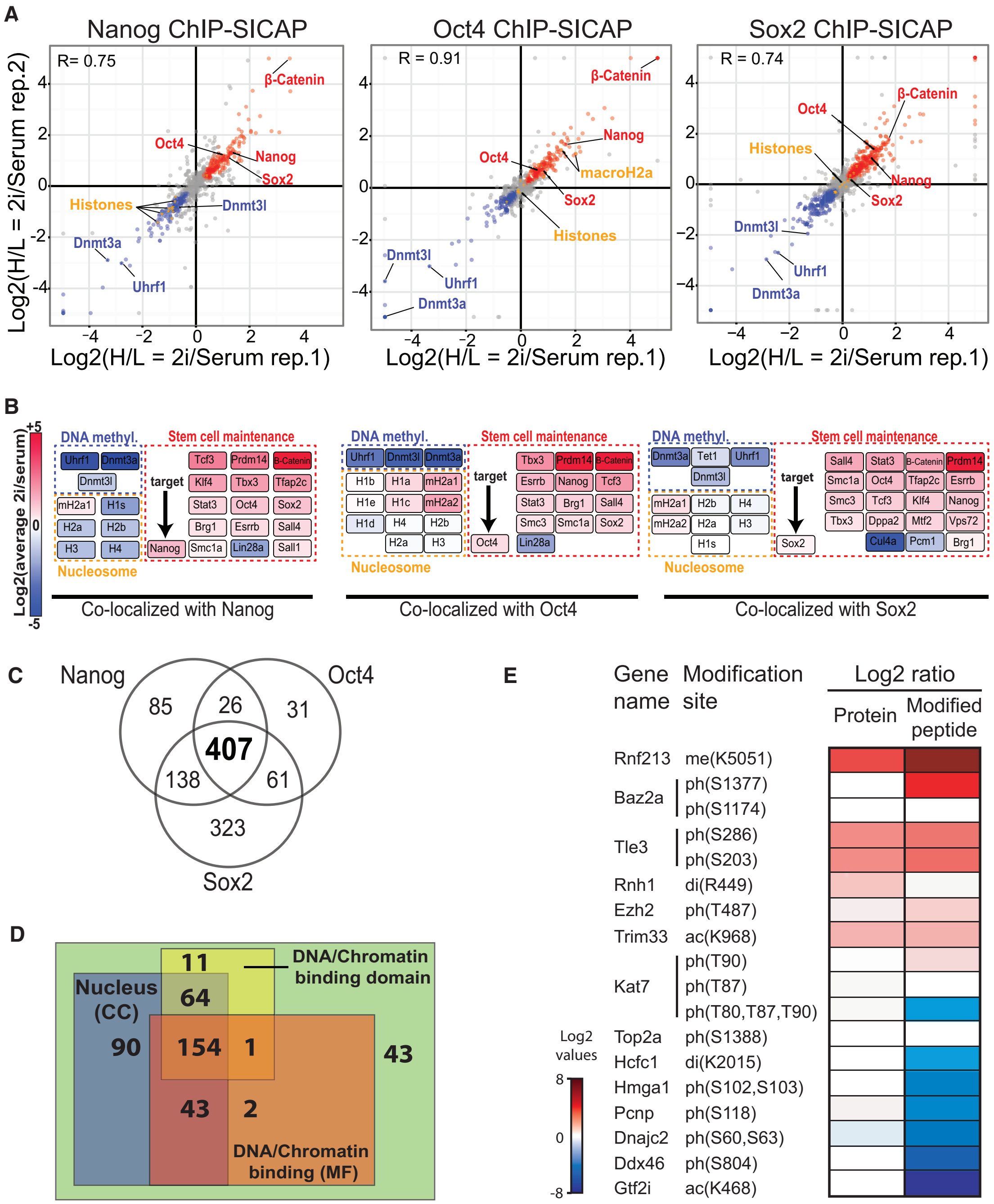

Figure 3. Comparative ChIP-SICAP between 2iL and Serum Conditions

(A) Scatterplots indicating the distribution and reproducibility of protein ratios in the three ChIP-SICAP experiments using Nanog, Oct4, and Sox2 as bait proteins. Proteins identified by the no-antibody control are not shown.

(B) Differential chromatin interaction of proteins involved in stem cell maintenance, nucleosomes, and de novo DNA methylation among the three ChIP-SICAP experiments.

(C) Overlap among proteins identified to colocalize with the three pluripotency master regulators. Proteins identified with no antibody control were subtracted.

(D) GO annotation of the proteins identified by ChIP-SICAP with Oct4, Sox2, and Nanog.

(E) Fold change of proteins and their modifications, comparing ChIP-SICAP data between 2iL and serum growth conditions. See also Figure S3 and Table S2.

Table S2E). Several PTMs differ in abundance between 2iL/serum, mostly following the trend of their cognate protein, with distinct exceptions (Figure 3E) suggesting a change in the stoichiometry of the modification in proteins associating with OSN in 2iL versus serum conditions. Although additional experiments will be required to confirm if these modifications are causally

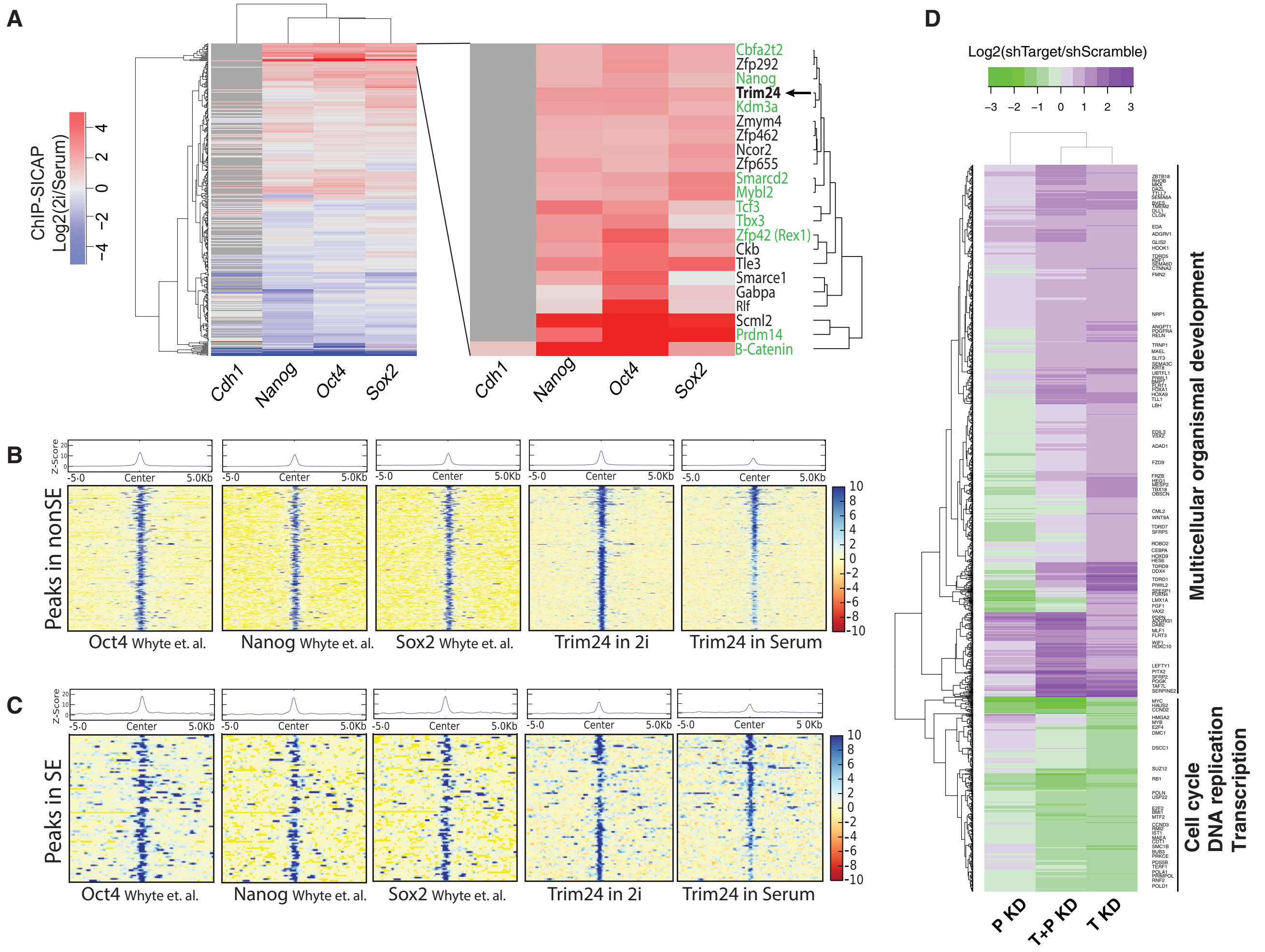

Figure 4. Integration of OSN ChIP-SICAP and Association of TRIM24 to the Pluripotency Network

(A) Hierarchical clustering of proteins identified by ChIP-SICAP using Oct4, Sox2, Nanog, and Cdh1 as bait proteins. Coloring was done according to ChIP-SICAP 2iL/serum protein ratio (log2). Zoom-in of the top cluster shows the enrichment of known (green) and so-far-unknown OSN-associated proteins (black).

(B) Trim24 ChIP-seq signal in 2iL and serum conditions compared to OSN signal in non-superenhancers (nonSE).

(C) Same as in (B), but for superenhancers (SE), as defined in (Whyte et al., 2013).

(D) Heatmap showing differentially expressed genes after Trim24 knockdown (fold change > 1.5, FDR < 0.01). T KD, Trim24 knockdown; P KD, p53 knock down; T+P KD, double knockdown of Trim24 and p53. See also Figure S4, Table S3, and Table S4.

involved in modulating protein interactions in chromatin, ChIP-SICAP may provide a starting point to investigate how PTMs shape chromatin-bound protein networks.

Trim24 Participates in the Pluripotency Network

The 407 proteins that were consistently enriched with OSN (Figure 3C; Tables S2A–S2D) were subjected to hierarchical clustering based on their ChIP-SICAP protein ratios between 2iL and serum conditions, showing high similarity between Oct4, Sox2, and Nanog experiments while Cdh1 remained as a separate group (Figure 4A). Interestingly, many established stem cell regulators were enriched in 2iL conditions by each of the three TFs (Figure 4A), indicating strong association with the OSN network in the naive pluripotent state. These include Nanog, β-catenin, Prdm14, Zfp42 (Rex1), Tcf7l1(Tcf3), Tbx3, and Kdm3a (Jmjd1a). Interestingly, Cbfa2t2, a transcriptional corepressor not previously known to interact with OSN, was identified very recently as a protein that regulates pluripotency and germline specification in mice by providing a scaffold to stabilize PRDM14 and OCT4 on chromatin (Tu et al., 2016). This is not only fully consistent with our observation of Cbfa2t2 in the OSN network but also provides an independent functional validation of our data.

Another candidate that we identified is Trim24, an E3-ubiquitin ligase that binds to combinatorially modified histones (Tsai et al., 2010). We performed ChIP-seq for Trim24 to identify its genome-wide occupancy in ESCs grown both in 2iL and serum media and compared this to genome occupancy of OSN (Table S3). Overall, Trim24 colocalized with OSN in 813 enhancers (Figures 4B and 4C; Tables S3B and S3C), including 88 of the 142 (62%)

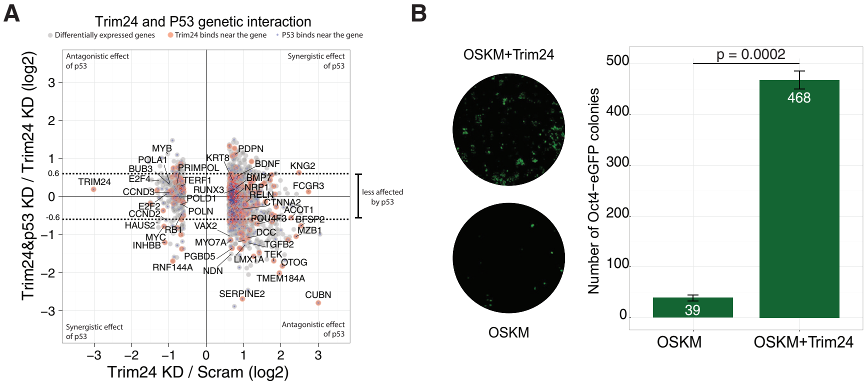

Figure 5. Mechanism and Function of Trim24

(A) Genetic interaction between Trim24 and p53. The scatterplot shows the genes differentially expressed after Trim24 knockdown. The effect of p53 knockdown on Trim24-target genes (y axis) was calculated by dividing expression change after p53-Trim24 double knockdown by expression change after Trim24 knockdown. Red dots indicate genes with a Trim24-binding site (<10 kb from the gene). Blue dots indicate genes with a p53-binding site (<10 kb from the gene).

(B) Trim24 increases the efficiency of reprogramming. (Left) iPS colonies were generated with Oct4, Sox2, Klf4, and c-Myc (OSKM) (bottom) or OSKM plus Trim24 (top) to compare the efficiency of reprogramming based on the number of Oct4-EGFP-positive colonies. (Right) Bar chart indicates the mean of three wells transduced by the vectors separately (error bars indicate SD; p value, Student's t test). See also Figure S5, Table S4, and Table S5.

previously reported superenhancers (Whyte et al., 2013). Additionally, Trim24 preferentially binds to 237 enhancers in 2iL-condition compared to only 27 in serum condition (FDR <0.05 and fold change >1.5; Tables S3D and S3E), which is in line with the high ChIP-SICAP ratio of Trim24 in 2iL/serum (Figure 4A). Interestingly, some of these enhancers are in close proximity to genes involved in either negative regulation of cell differentiation or positive regulation of cell proliferation (Table S3F), thus suggesting a regulatory role for Trim24 in processes that are fundamental to pluripotency.

Trim24 Regulates Cell Proliferation and Differentiation Gene Expression in Mouse ESCs

To better understand how Trim24 functions mechanistically in mouse ESCs, we performed knockdown of Trim24 using short hairpin RNA (shRNA) for 24 hr, followed by mRNA sequencing. We observed dysregulation of 1,562 genes (adjusted p value < 0.01 and fold change >1.5) (Figure 4D; Table S4A). Interestingly, developmental genes were upregulated, including genes involved in neural differentiation (e.g., Bdnf, Nrcam, Tgfb2, and Reln), immune system (Fcgr3 and Cd34), muscle differentiation (Myh6 and Myh7), and spermatogenesis (Dazl, Tdrd1, and Piwil2). On the other hand, numerous genes with central roles in cell cycle and proliferation were downregulated, (e.g., Myc, Myb, RB1, CyclinD2, and CyclinD3) (Figure S4F; Table S4B). Remarkably Bmi1, Rnf2, Suz12, and Mtf2 were downregulated, which are well-known members of the PRC1 and PRC2 complexes (Table S4C). Altogether, this result indicates that Trim24 is required to suppress developmental gene, and to maintain expression of genes involved in proliferation, cell cycle, and DNA replication.

Previously, Allton et al. have shown that Trim24 knockdown in mouse ESCs leads to p53-mediated apoptosis (Allton et al., 2009). To test coregulation of genes by Trim24 and p53, we carried out p53 knockdown as well as double knockdown of Trim24 and p53. As a result of p53 knockdown, 1,801 genes were deregulated, of which 353 genes were overlapping with Trim24 knockdown (Figure S4D; Table S4A). We compared these data to a Trim24-p53 double knockdown to distinguish synergistic and antagonistic effects (Figure 5A), revealing that 73.4% of the Trim24 target genes are regulated independent of p53. However, the effect of p53 on 18.1% and 8.4% of the Trim24 targets is antagonistic and synergistic, respectively. For instance, p53 has an antagonistic effect on Myb expression, rescuing Trim24 knockdown-mediated downregulation of Myb (Figure 5A; Table S4A). Conversely, p53 and Trim24 have synergistic positive effects on Myc expression.

Among the 1,562 genes that are differentially expressed after Trim24 knockdown, 198 genes (11%) are located near (<10 kb) the Trim24 binding sites on the genome (Figure 5A; Table S4D). Moreover, 68 ESC enhancers with Trim24 occupancies are located near the differentially expressed genes (Figure S4E). The comparison of the genome-wide occupancy of p53 in mouse ESCs (Li et al., 2012b) with our Trim24 ChIP-seq data revealed that 17 ES superenhancers are cobound by p53 and Trim24 (Figure S4B). Remarkably, this includes the superenhancers of pluripotency genes such as Nanog, Prdm14, Sox2, and Tbx3. Although Trim24 binds preferentially to these loci in 2iL media (Figure S4C), knockdown of Trim24 had no significant effect on the expression of these genes, at least under the used conditions (knockdown for 24 hr).

Altogether, these data indicate that Trim24 functions to activate expression of cell cycle, DNA replication, and polycomb components and to suppress expression of developmental genes largely independently of p53.

Trim24 Significantly Improves the Efficiency of Somatic Cell Reprogramming

Since our observations position Trim24 in the OSN network, regulating the expression of cell cycle and developmental genes, we tested if Trim24 can promote the generation of iPS cells. We coexpressed Trim24 with OSKM in a doxycycline (Dox)-inducible reprogramming system (Stadtfeld et al., 2010) to induce formation of iPS cells from secondary MEFs. As a result, we observed that expression of Trim24 together with OSKM increased the number of Oct4-EGFP-positive colonies from 39 to 468 per plate compared to OSKM alone, i.e., an increase of 12-fold (Figures 5B and S5). This suggests that Trim24 stabilizes the transcriptional program imposed by OSKM to more efficiently establish and maintain pluripotency.

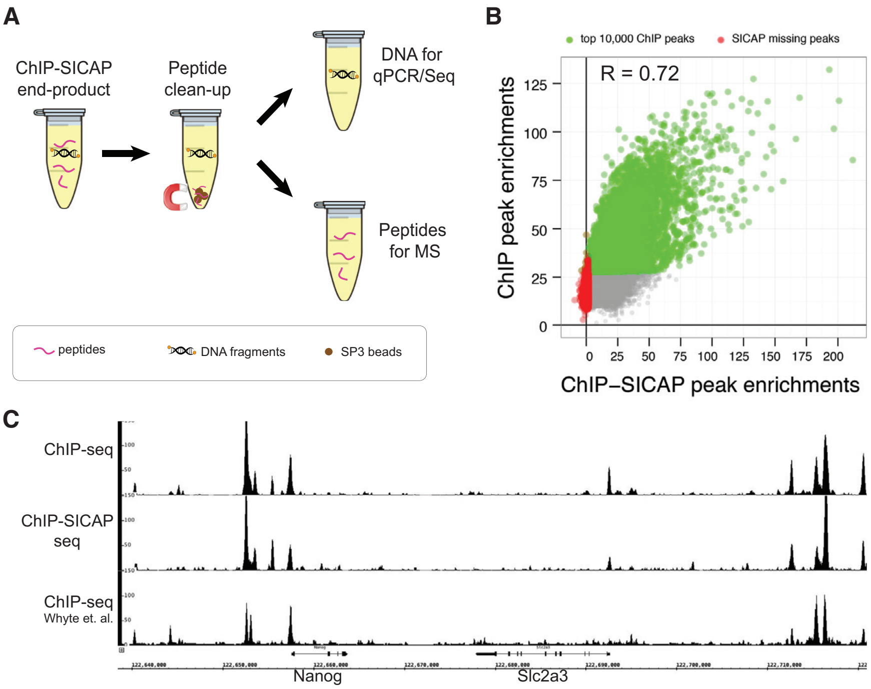

Figure 6. Retrieving DNA and Peptides from the Same ChIP-SICAP Assay

(A) After tryptic digestion, peptides are cleaned up by SP3 protocol (Hughes et al., 2014) using magnetic beads. DNA remains in solution while peptides are trapped on the beads and can be retrieved separately for sequencing and mass spectrometry.

(B) Enrichment of peaks called in a normal Nanog ChIP-seq in comparison to their enrichment after isolation of DNA via ChIP-SICAP. Green dots indicate top 10,000 enriched peaks. Red dots indicate peaks <2-fold enriched by ChIP-SICAP.

(C) Aligned ChIP-seq profiles for Nanog near the Nanog locus. Traces indicate profiles after DNA retrieval via classical ChIP-seq (top) and ChIP-SICAP (middle) compared to ChIP-seq data from Whyte et al. (2013) (bottom). See also Figure S6 and Table S7.

Recovery of DNA after ChIP-SICAP Permits ChIP-Seq from the Same Sample

We next investigated the feasibility of retrieving both proteins and DNA after ChIP-SICAP, aiming to identify the proteins that colocalize with the bait (by MS) as well as its genomic binding site (by NGS) from the same sample. We therefore verified the presence of DNA in the supernatant of samples treated with SP3 (Hughes et al., 2014), the last step in the ChIP-SICAP procedure used for peptide cleanup and removal of detergents (Figure 6A). Indeed, qPCR on DNA purified after Nanog ChIP-SICAP recovered the Nanog promoter, but not flanking regions (Figure S6), consistent with the notion that Nanog binds to its own promoter. Next, although the recovered DNA was end-biotinylated, we successfully prepared the library for NGS without any change in Illumina sample prep protocol. Strikingly, when comparing the result of regular ChIP-seq and ChIP-SICAP-seq using the same Nanog antibody, we identified a very similar number of peaks with very large overlap (94%, Figure S6B and Table S6) and similar enrichment (Figure S6C). Among the top 10,000 enriched ChIP-seq peaks, only 33 peaks were not enriched by ChIP-SICAP, indicating that recovery of DNA by biotin labeling and streptavidin purification is very efficient in SICAP. Moreover, the recovery of the major ChIP-seq peaks without the introduction of artifactual peaks suggests that TdT biotinylates chromatin fragments in an unbiased manner. As a result, ChIP-SICAP can be used for the simultaneous analysis of proteins and DNA in an integrative workflow, to obtain highly complementary information on the identity of colocalized proteins as well as genomic binding sites of the bait protein.

DISCUSSION

We have designed ChIP-SICAP to characterize the proteins that converge on chromatin with a protein of interest in its DNA-bound state, aimed to gain insight in the composition and function of the protein network around transcription factors and transcriptional regulators. We applied ChIP-SICAP to Oct4, Sox2, and Nanog in mouse ESCs to better characterize the protein network operating in the core of pluripotency in a quantitative and context-dependent manner and demonstrated the power of this approach by identifying and validating Trim24 as a protein that physically colocalizes and functionally interacts with core pluripotency factors.

Compared to other methods, ChIP-SICAP benefits from the sequential enrichment of the bait protein and the DNA it is cross-linked to. In particular, TdT-mediated biotinylation of DNA and subsequent capture by streptavidin critically contribute to the specificity of the approach by allowing stringent washing to efficiently remove common contaminants, including the IP antibody (Figure S1), while providing evidence that the bait and colocalizing proteins bind to chromatin. A distinct advantage of ChIP-SICAP over conventional coIP is its ability to identify proteins that colocalize within a short distance on DNA, revealing functional connections between proteins that are not necessarily mediated by direct physical interactions. This is highly relevant in the light of recent data showing that interactions between many cooperative TFs are mediated by DNA (Jolma et al., 2015) rather than direct protein-protein interactions.

Abundance ranking of proteins identified by ChIP-SICAP provides a characteristic signature (Figures 2A and S3A) allowing for quality control of the obtained results. Following histones as the most abundant proteins, the bait protein itself typically ranks among the top candidates, thereby validating the specificity of the antibody and thus satisfying the recommendations that were recently proposed for the quality control of antibodies in affinity-purification strategies (Marcon et al., 2015). This is followed by dozens to hundreds of proteins with lower abundance, which we interpret as proteins that colocalize with the bait at decreasing frequency along the genome. This overall pattern, in combination with the identification of bait-specific protein

profiles (Figure 4A) and the underrepresentation of common contaminants (Figure 2B), argues against the possibility of systematic calling of false interactions due to overcrosslinking. Yet we cannot exclude the possibility that some of the interactions reported here may be indirect.

We combined ChIP-SICAP with SILAC labeling, demonstrating both tight interconnectivity between 400 proteins that colocalize around the core pluripotency factors Oct4, Sox2, and Nanog and that the composition of this network depends on the pluripotent state (Figure 3A).

We focused our attention to Trim24 as a protein not known to partake in the pluripotency network but that tightly clustered with well-established pluripotency factors, especially in 2iL conditions (Figure 4A). Trim24, also known as transcriptional intermediary factor 1a (Tif1a), has been identified as a E3-ubiquitin ligase but also as a reader of histone modifications (Tsai et al., 2010). Functionally, Trim24 has been shown to modulate transcription in mouse zygotes, by moving from the cytoplasm to the nucleus and to activate transcription of the embryonic genome (Torres-Padilla and Zernicka-Goetz, 2006). Although Trim24 has never been directly linked to pluripotency, large-scale studies suggest that its expression closely follows the trend of bona fide pluripotency factors showing increased expression during reprogramming both at the transcript (Polo et al., 2012) and the protein level (Benevento et al., 2014; Hansson et al., 2012). Our data demonstrate not only that Trim24 colocalizes to many OSN binding sites in the genome (Figures 4B and 4C) but also that it activates transcription of cell cycle and DNA replication genes while suppressing differentiation genes. These characteristics likely contribute to its role in promoting OSKM-mediated generation of iPS cells (Figure 5B).

Intriguingly, recent studies have correlated elevated expression of Trim24 with poor patient prognosis in various tumor entities (Cui et al., 2013; Li et al., 2012a; Liu et al., 2014; Zhang et al., 2015). Furthermore, ectopic expression of Trim24 induced malignant transformation in epithelial cells (Pathiraja et al., 2015), while its knockdown in colon cancer cells induced apoptosis (Wang et al., 2014). Collectively, this suggests that the main function of Trim24 resides in enhancing cell proliferation, thereby contributing to critical hallmarks both of pluripotency and cancer.

Altogether, we have demonstrated that ChIP-SICAP is a powerful tool to gain a better understanding of transcriptional networks in general, and in pluripotency in particular. Considering that this method can be generically applied to any other cell type or chromatin protein, ChIP-SICAP should prove a useful and versatile tool to identify proteins that associate with a variety of TFs, transcriptional regulators, and posttranslationally modified histones. We anticipate that future use of ChIP-SICAP will extend to the analysis of protein translocation to chromatin as a mechanism to determine cell fate, to investigate the correlation between chromatin-association of TFs and their local histone-PTM landscape, and to examine the role of PTMs in protein association to chromatin. Its utility is further enhanced by the ability to simultaneously obtain DNA for high-quality ChIP-seq, to obtain highly complementary data types (protein colocalization and genome occupancy) in an integrated workflow.

Limitations

One of the limitations of ChIP-SICAP is the need for a ChIP-grade antibody. Thereby it suffers from the same restriction as ChIP-seq, but with the distinction that the verification of the antibody specificity is an inherent part of ChIP-SICAP data analysis. Therefore, even antibodies against nonclassical chromatin proteins may be tested and validated by ChIP-SICAP. The need for protein-specific antibodies may be bypassed by employing CRISPR/Cas9 technologies to insert an affinity tag (e.g., HA or FLAG) in the coding sequence of the gene of interest. As yet another approach, computational methods such as DeepBind (Alipanahi et al., 2015) may predict the score of binding (here colocalization) for a given protein on the binding sites of the bait, although this is limited to proteins for which a motif is known.

The sensitivity of ChIP-SICAP may be limited by the low efficiency of IP (usually ~1%) and by limitations in mass spectrometry to detect very low-abundance peptides. Consequently, proteins that colocalize with the bait protein at many genomic locations will be preferentially identified. The power of ChIP-SICAP resides in its unbiased protein identification to thereby suggest novel chromatin factors; however, their frequency and the exact sites of colocalization need to be validated by ChIP-qPCR for individual sites, or by ChIP-seq for global profiling across the genome (as performed in this study for Trim24).

EXPERIMENTAL PROCEDURES

Cell Culture and Cell Fixation

Mouse ESCs (46c) were grown feeder free on 0.2% gelatinized cell culture plates in either traditional ES media with serum or 2iL-media (2i+LIF). Chromatin was crosslinked by suspending cells in 1.5% formaldehyde (Pierce) for 15 min, quenched in 125 mM Glycine (Merck), and stored at –80°C until use.

ChIP-SICAP and Mass Spectrometry

Chromatin from 24 million fixed ESCs sheared by sonication, followed by immunoprecipitation with a suitable antibody. After capture on protein A beads, DNA was biotinylated by TdT in the presence of biotin-11-ddUTP and eluted, and protein-DNA complexes were bound to streptavidin beads. Proteins were digested with trypsin, and resulting peptides were fractionated by high pH reverse-phase chromatography and analyzed using LC-MS on a Orbitrap Velos Pro or Q-Exactive mass spectrometer (Thermo Fisher Scientific). A detailed protocol and details for data analysis can be found in the Supplemental Information.

ChIP-Seq and Data Analysis

After ChIP on crosslinked and sheared chromatin, protein was digested with Proteinase K, and DNA was purified using phenol/chloroform isoamyl alcohol and then precipitated. The libraries were prepared for Illumina sequencing, and sequencing was carried out by Illumina HiSeq 2000 according to the manufacturer's protocols.

Trim24 and P53 Knockdown and RNA-Seq Analysis

Knockdown (KD) of Trim24 and p53 was carried out by the lentiviral vectors shTrim24 (TRCN0000088518) and shTrp53 (TRCN0000310844), respectively (Sigma), in three independent transductions. Forty-eight hours after infection, ESCs were lyzed and RNA was extracted for mRNA-seq (following the standard Illumina TruSeq protocol for library generation) and RT-qPCR.

ACCESSION NUMBERS

Mass spectrometry data are available via ProteomeXchange with identifier PXD003798. ChIP-seq and RNA-seq data are available via ArrayExpress with identifiers E-MTAB-3802 and E-MTAB-4893, respectively.

SUPPLEMENTAL INFORMATION

Supplemental Information includes six figures, six tables, and Supplemental Experimental Procedures and can be found with this article at http://dx.doi.org/10.1016/j.molcel.2016.09.019.

AUTHOR CONTRIBUTIONS

M.-R.R. and J.K. designed the studies and analyzed the data. M.-R.R. performed all experiments. G.S. analyzed mass spectrometry data. C.G. analyzed sequencing data. M.-R.R. and J.K. wrote the manuscript with input from all authors.

ACKNOWLEDGMENTS

M.-R.R. is supported by a fellowship from the Darwin Trust of Edinburgh. We gratefully acknowledge the Genomics Core Facility, the Proteomics Core Facility, and the Advanced Light Micrososcopy Facility at EMBL for expert technical support.

Received: March 30, 2016
Revised: July 11, 2016
Accepted: September 14, 2016
Published: October 20, 2016

REFERENCES

Alipanahi, B., Delong, A., Weirauch, M.T., and Frey, B.J. (2015). Predicting the sequence specificities of DNA- and RNA-binding proteins by deep learning. Nat. Biotechnol. *33*, 831–838.

Allton, K., Jain, A.K., Herz, H.M., Tsai, W.W., Jung, S.Y., Qin, J., Bergmann, A., Johnson, R.L., and Barton, M.C. (2009). Trim24 targets endogenous p53 for degradation. Proc. Natl. Acad. Sci. USA *106*, 11612–11616.

Benevento, M., Tonge, P.D., Puri, M.C., Hussein, S.M., Cloonan, N., Wood, D.L., Grimmond, S.M., Nagy, A., Munoz, J., and Heck, A.J. (2014). Proteome adaptation in cell reprogramming proceeds via distinct transcriptional networks. Nat. Commun. *5*, 5613.

Boyer, L.A., Lee, T.I., Cole, M.F., Johnstone, S.E., Levine, S.S., Zucker, J.P., Guenther, M.G., Kumar, R.M., Murray, H.L., Jenner, R.G., et al. (2005). Core transcriptional regulatory circuitry in human embryonic stem cells. Cell *122*, 947–956.

Boyer, L.A., Plath, K., Zeitlinger, J., Brambrink, T., Medeiros, L.A., Lee, T.I., Levine, S.S., Wernig, M., Tajonar, A., Ray, M.K., et al. (2006). Polycomb complexes repress developmental regulators in murine embryonic stem cells. Nature *441*, 349–353.

Buschbeck, M., Uribesalgo, I., Wibowo, I., Rué, P., Martin, D., Gutierrez, A., Morey, L., Guigó, R., López-Schier, H., and Di Croce, L. (2009). The histone variant macroH2A is an epigenetic regulator of key developmental genes. Nat. Struct. Mol. Biol. *16*, 1074–1079.

Cui, Z., Cao, W., Li, J., Song, X., Mao, L., and Chen, W. (2013). TRIM24 overexpression is common in locally advanced head and neck squamous cell carcinoma and correlates with aggressive malignant phenotypes. PLoS ONE *8*, e63887.

Dunham, W.H., Mullin, M., and Gingras, A.C. (2012). Affinity-purification coupled to mass spectrometry: basic principles and strategies. Proteomics *12*, 1576–1590.

Engelen, E., Brandsma, J.H., Moen, M.J., Signorile, L., Dekkers, D.H., Demmers, J., Kockx, C.E., Ozgür, Z., van IJcken, W.F., van den Berg, D.L., and Poot, R.A. (2015). Proteins that bind regulatory regions identified by histone modification chromatin immunoprecipitations and mass spectrometry. Nat. Commun. *6*, 7155.

Ferber, E.C., Kajita, M., Wadlow, A., Tobiansky, L., Niessen, C., Ariga, H., Daniel, J., and Fujita, Y. (2008). A role for the cleaved cytoplasmic domain of E-cadherin in the nucleus. J. Biol. Chem. *283*, 12691–12700.

Gagliardi, A., Mullin, N.P., Ying Tan, Z., Colby, D., Kousa, A.I., Halbritter, F., Weiss, J.T., Felker, A., Bezstarosti, K., Favaro, R., et al. (2013). A direct physical interaction between Nanog and Sox2 regulates embryonic stem cell self-renewal. EMBO J. *32*, 2231–2247.

Gamble, M.J., Frizzell, K.M., Yang, C., Krishnakumar, R., and Kraus, W.L. (2010). The histone variant macroH2A1 marks repressed autosomal chromatin, but protects a subset of its target genes from silencing. Genes Dev. *24*, 21–32.

Hansson, J., Rafiee, M.R., Reiland, S., Polo, J.M., Gehring, J., Okawa, S., Huber, W., Hochedlinger, K., and Krijgsveld, J. (2012). Highly coordinated proteome dynamics during reprogramming of somatic cells to pluripotency. Cell Rep. *2*, 1579–1592.

Huang, X., and Wang, J. (2014). The extended pluripotency protein interactome and its links to reprogramming. Curr. Opin. Genet. Dev. *28*, 16–24.

Hughes, C.S., Foehr, S., Garfield, D.A., Furlong, E.E., Steinmetz, L.M., and Krijgsveld, J. (2014). Ultrasensitive proteome analysis using paramagnetic bead technology. Mol. Syst. Biol. *10*, 757.

Ji, X., Dadon, D.B., Abraham, B.J., Lee, T.I., Jaenisch, R., Bradner, J.E., and Young, R.A. (2015). Chromatin proteomic profiling reveals novel proteins associated with histone-marked genomic regions. Proc. Natl. Acad. Sci. USA *112*, 3841–3846.

Jolma, A., Yin, Y., Nitta, K.R., Dave, K., Popov, A., Taipale, M., Enge, M., Kivioja, T., Morgunova, E., and Taipale, J. (2015). DNA-dependent formation of transcription factor pairs alters their binding specificity. Nature *527*, 384–388.

Jones, N.A., and Dive, C. (1999). Cell sensitivity assays : detection of apoptotic cells in vitro using the TUNEL assay. Methods Mol. Med. *28*, 31–38.

Lai, Y.S., Chang, C.W., Pawlik, K.M., Zhou, D., Renfrow, M.B., and Townes, T.M. (2012). SRY (sex determining region Y)-box2 (Sox2)/poly ADP-ribose polymerase 1 (Parp1) complexes regulate pluripotency. Proc. Natl. Acad. Sci. USA *109*, 3772–3777.

Lambert, J.P., Mitchell, L., Rudner, A., Baetz, K., and Figeys, D. (2009). A novel proteomics approach for the discovery of chromatin-associated protein networks. Mol. Cell. Proteomics *8*, 870–882.

Laugesen, A., and Helin, K. (2014). Chromatin repressive complexes in stem cells, development, and cancer. Cell Stem Cell *14*, 735–751.

Lee, T.I., Jenner, R.G., Boyer, L.A., Guenther, M.G., Levine, S.S., Kumar, R.M., Chevalier, B., Johnstone, S.E., Cole, M.F., Isono, K., et al. (2006). Control of developmental regulators by Polycomb in human embryonic stem cells. Cell *125*, 301–313.

Li, H., Sun, L., Tang, Z., Fu, L., Xu, Y., Li, Z., Luo, W., Qiu, X., and Wang, E. (2012a). Overexpression of TRIM24 correlates with tumor progression in non-small cell lung cancer. PLoS ONE *7*, e37657.

Li, M., He, Y., Dubois, W., Wu, X., Shi, J., and Huang, J. (2012b). Distinct regulatory mechanisms and functions for p53-activated and p53-repressed DNA damage response genes in embryonic stem cells. Mol. Cell *46*, 30–42.

Li, X., Wang, W., Wang, J., Malovannaya, A., Xi, Y., Li, W., Guerra, R., Hawke, D.H., Qin, J., and Chen, J. (2015). Proteomic analyses reveal distinct chromatin-associated and soluble transcription factor complexes. Mol. Syst. Biol. *11*, 775.

Liu, X., Huang, Y., Yang, D., Li, X., Liang, J., Lin, L., Zhang, M., Zhong, K., Liang, B., and Li, J. (2014). Overexpression of TRIM24 is associated with the onset and progress of human hepatocellular carcinoma. PLoS ONE *9*, e85462.

Loh, Y.H., Wu, Q., Chew, J.L., Vega, V.B., Zhang, W., Chen, X., Bourque, G., George, J., Leong, B., Liu, J., et al. (2006). The Oct4 and Nanog transcription network regulates pluripotency in mouse embryonic stem cells. Nat. Genet. *38*, 431–440.

Mallanna, S.K., Ormsbee, B.D., Iacovino, M., Gilmore, J.M., Cox, J.L., Kyba, M., Washburn, M.P., and Rizzino, A. (2010). Proteomic analysis of Sox2-associated proteins during early stages of mouse embryonic stem cell differentiation identifies Sox21 as a novel regulator of stem cell fate. Stem Cells *28*, 1715–1727.

Marcon, E., Jain, H., Bhattacharya, A., Guo, H., Phanse, S., Pu, S., Byram, G., Collins, B.C., Dowdell, E., Fenner, M., et al. (2015). Assessment of a method to characterize antibody selectivity and specificity for use in immunoprecipitation. Nat. Methods *12*, 725–731.

Marks, H., Kalkan, T., Menafra, R., Denissov, S., Jones, K., Hofemeister, H., Nichols, J., Kranz, A., Stewart, A.F., Smith, A., and Stunnenberg, H.G. (2012). The transcriptional and epigenomic foundations of ground state pluripotency. Cell *149*, 590–604.

Mellacheruvu, D., Wright, Z., Couzens, A.L., Lambert, J.P., St-Denis, N.A., Li, T., Miteva, Y.V., Hauri, S., Sardiu, M.E., Low, T.Y., et al. (2013). The CRAPome: a contaminant repository for affinity purification-mass spectrometry data. Nat. Methods *10*, 730–736.

Mohammed, H., Taylor, C., Brown, G.D., Papachristou, E.K., Carroll, J.S., and D'Santos, C.S. (2016). Rapid immunoprecipitation mass spectrometry of endogenous proteins (RIME) for analysis of chromatin complexes. Nat. Protoc. *11*, 316–326.

Nelson, J.D., Denisenko, O., and Bomsztyk, K. (2006). Protocol for the fast chromatin immunoprecipitation (ChIP) method. Nat. Protoc. *1*, 179–185.

Nichols, J., and Smith, A. (2009). Naive and primed pluripotent states. Cell Stem Cell *4*, 487–492.

Novo, C.L., Tang, C., Ahmed, K., Djuric, U., Fussner, E., Mullin, N.P., Morgan, N.P., Hayre, J., Sienerth, A.R., Elderkin, S., et al. (2016). The pluripotency factor Nanog regulates pericentromeric heterochromatin organization in mouse embryonic stem cells. Genes Dev. *30*, 1101–1115.

Ohta, S., Bukowski-Wills, J.C., Sanchez-Pulido, L., Alves, Fde.L., Wood, L., Chen, Z.A., Platani, M., Fischer, L., Hudson, D.F., Ponting, C.P., et al. (2010). The protein composition of mitotic chromosomes determined using multiclassifier combinatorial proteomics. Cell *142*, 810–821.

Pardo, M., Lang, B., Yu, L., Prosser, H., Bradley, A., Babu, M.M., and Choudhary, J. (2010). An expanded Oct4 interaction network: implications for stem cell biology, development, and disease. Cell Stem Cell *6*, 382–395.

Pathiraja, T.N., Thakkar, K.N., Jiang, S., Stratton, S., Liu, Z., Gagea, M., Shi, X., Shah, P.K., Phan, L., Lee, M.H., et al. (2015). TRIM24 links glucose metabolism with transformation of human mammary epithelial cells. Oncogene *34*, 2836–2845.

Polo, J.M., Anderssen, E., Walsh, R.M., Schwarz, B.A., Nefzger, C.M., Lim, S.M., Borkent, M., Apostolou, E., Alaei, S., Cloutier, J., et al. (2012). A molecular roadmap of reprogramming somatic cells into iPS cells. Cell *151*, 1617–1632.

Stadtfeld, M., Maherali, N., Borkent, M., and Hochedlinger, K. (2010). A reprogrammable mouse strain from gene-targeted embryonic stem cells. Nat. Methods *7*, 53–55.

Torres-Padilla, M.E., and Zernicka-Goetz, M. (2006). Role of TIF1alpha as a modulator of embryonic transcription in the mouse zygote. J. Cell Biol. *174*, 329–338.

Tsai, W.W., Wang, Z., Yiu, T.T., Akdemir, K.C., Xia, W., Winter, S., Tsai, C.Y., Shi, X., Schwarzer, D., Plunkett, W., et al. (2010). TRIM24 links a non-canonical histone signature to breast cancer. Nature *468*, 927–932.

Tu, S., Narendra, V., Yamaji, M., Vidal, S.E., Rojas, L.A., Wang, X., Kim, S.Y., Garcia, B.A., Tuschl, T., Stadtfeld, M., and Reinberg, D. (2016). Co-repressor CBFA2T2 regulates pluripotency and germline development. Nature *534*, 387–390.

van den Berg, D.L., Snoek, T., Mullin, N.P., Yates, A., Bezstarosti, K., Demmers, J., Chambers, I., and Poot, R.A. (2010). An Oct4-centered protein interaction network in embryonic stem cells. Cell Stem Cell *6*, 369–381.

Wang, C.I., Alekseyenko, A.A., LeRoy, G., Elia, A.E., Gorchakov, A.A., Britton, L.M., Elledge, S.J., Kharchenko, P.V., Garcia, B.A., and Kuroda, M.I. (2013). Chromatin proteins captured by ChIP-mass spectrometry are linked to dosage compensation in Drosophila. Nat. Struct. Mol. Biol. *20*, 202–209.

Wang, J., Zhu, J., Dong, M., Yu, H., Dai, X., and Li, K. (2014). Knockdown of tripartite motif containing 24 by lentivirus suppresses cell growth and induces apoptosis in human colorectal cancer cells. Oncol. Res. *22*, 39–45.

Whyte, W.A., Orlando, D.A., Hnisz, D., Abraham, B.J., Lin, C.Y., Kagey, M.H., Rahl, P.B., Lee, T.I., and Young, R.A. (2013). Master transcription factors and mediator establish super-enhancers at key cell identity genes. Cell *153*, 307–319.

Ying, Q.L., Wray, J., Nichols, J., Batlle-Morera, L., Doble, B., Woodgett, J., Cohen, P., and Smith, A. (2008). The ground state of embryonic stem cell self-renewal. Nature *453*, 519–523.

Zee, B.M., Alekseyenko, A.A., McElroy, K.A., and Kuroda, M.I. (2016). Streamlined discovery of cross-linked chromatin complexes and associated histone modifications by mass spectrometry. Proc. Natl. Acad. Sci. USA *113*, 1784–1789.

Zhang, L.H., Yin, A.A., Cheng, J.X., Huang, H.Y., Li, X.M., Zhang, Y.Q., Han, N., and Zhang, X. (2015). TRIM24 promotes glioma progression and enhances chemoresistance through activation of the PI3K/Akt signaling pathway. Oncogene *34*, 600–610.

Molecular Cell, Volume *64*

Supplemental Information

Expanding the Circuitry of Pluripotency by Selective Isolation of Chromatin-Associated Proteins

Mahmoud-Reza Rafiee, Charles Girardot, Gianluca Sigismondo, and Jeroen Krijgsveld

Figure S1

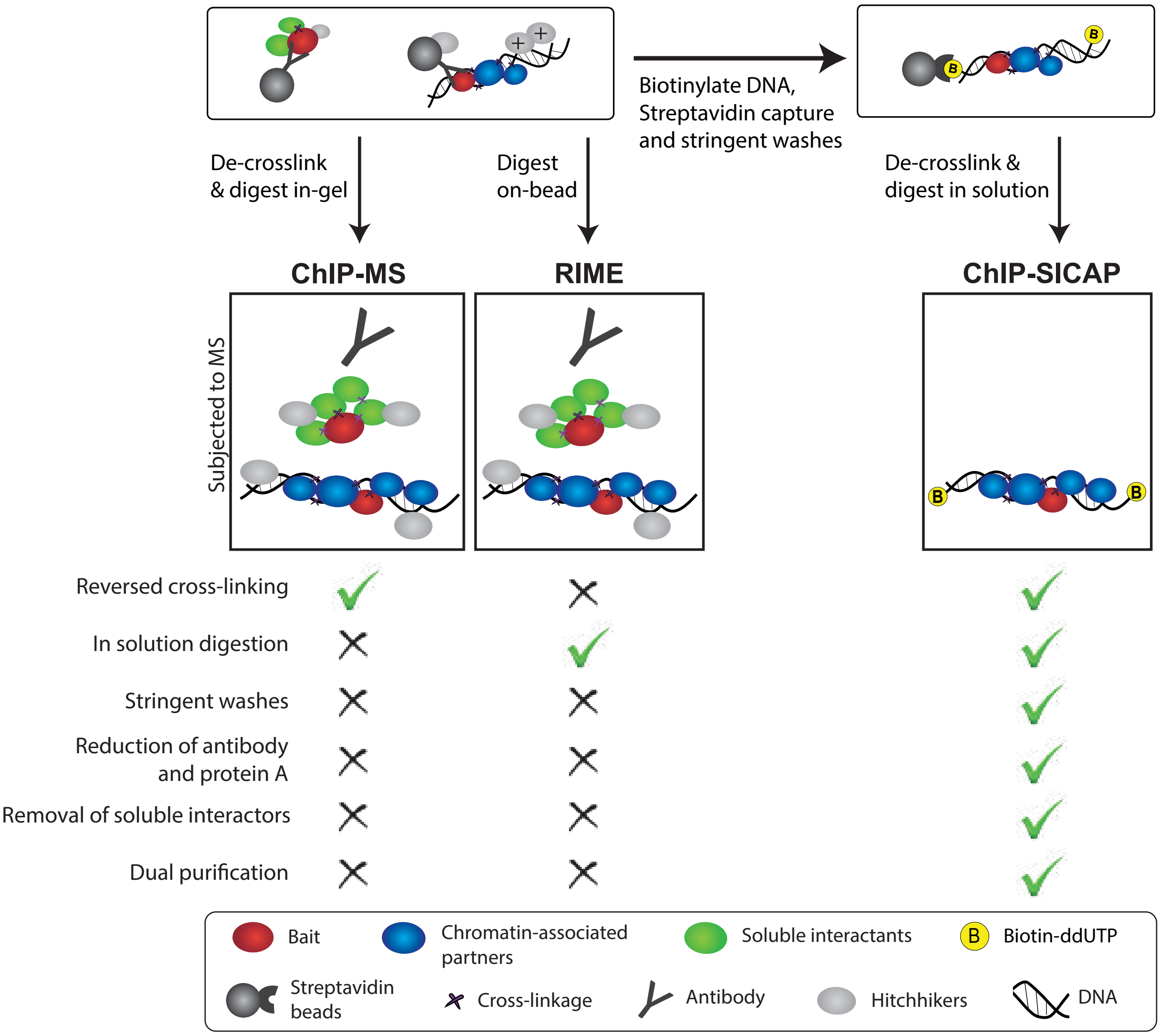

Biotinylate DNA, Streptavidin capture and stringent washes
De-crosslink & digest in-gel
Digest on-bead
De-crosslink & digest in solution
ChIP-MS
RIME
ChIP-SICAP
Subjected to MS
B
Reversed cross-linking
In solution digestion
Stringent washes
Reduction of antibody and protein A
Removal of soluble interactors
Dual purification
Bait
Chromatin-associated partners
Soluble interactants
Biotin-ddUTP
Streptavidin beads
Cross-linkage
Antibody
Hitchhikers
DNA

Figure S2

A

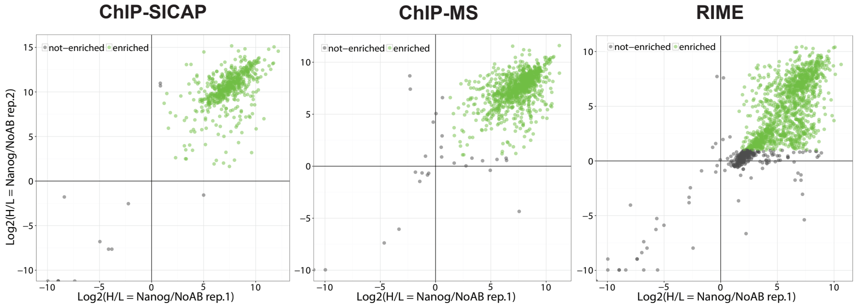

B

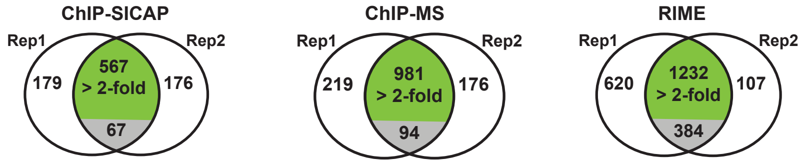

C

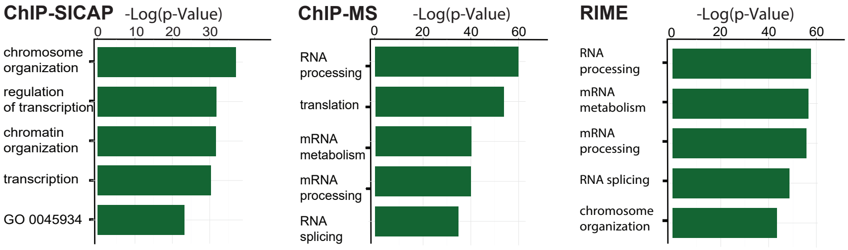

D

Sequence of the IgG peptides	RIME		ChIP-MS		ChIP-SICAP	
	R1	R2	R1	R2	R1	R2
ALPAPIEK	+	+	+	+	+	+
ALPAPIEKTISK	-	-	+	+	-	-
APSVFPLAPCCGDTPSSTVTLGCLVK	-	-	+	+	-	-
DTLMISR	+	+	+	+	+	+
EQQFNSTIR	+	+	+	+	-	-
GDVFTCSVMHEALHNHYTQK	-	-	+	+	-	-
GYLPEPVTVTWNSGTLTNGVR	-	+	-	-	-	-
LSVPTSEWQR	+	+	+	+	-	-
QSSGLYSLSSVVSVTSSSQPVTCNVAHPATNTK	-	-	+	+	-	-
TARPPLREQQFNSTIR	-	-	+	+	-	-
TTPAVLDSDGSYFLYNK	-	-	+	+	-	-
VHNKALPAPIEK	-	-	+	+	-	-
VYTMGPPR	+	+	+	+	-	-
VYTMGPPREELSSR	+	+	+	+	-	-

E

Sequence of the Protein A peptides	RIME		ChIP-MS		ChIP-SICAP	
	R1	R2	R1	R2	R1	R2
ADNNFNK	+	-	-	-	-	-
ADNNFNKEQQNAFYEILNMPNLNEEQR	+	+	-	-	-	-
DDPSQSANLLAEAK	+	+	+	+	+	+
DDPSQSANLLAEAKK	+	+	-	-	-	-
DDPSQSANLLAEAKKLNDAQAPK	+	+	-	-	-	-
DDPSQSANLLSEAK	+	+	-	-	-	-
DDPSQSANVLGEAQK	+	+	+	+	+	+
DDPSQSANVLGEAQKLNDSQAPK	+	+	-	-	-	-
DDPSQSTNVLGEAK	+	+	+	+	+	+
DDPSQSTNVLGEAKK	+	+	-	-	-	-
DDPSQSTNVLGEAKKLNESQAPK	+	+	-	-	-	-
DDPSVSKEILAEAK	+	+	+	+	-	-
DDPSVSKEILAEAKK	+	+	-	-	-	-
DQQSAFYEILNMPNLNEAQR	+	+	-	-	-	-
EILAEAK	+	+	+	+	-	-
EILAEAKKLNDAQAPK	+	+	-	-	-	-
EQQNAFYEILHLPNLNEEQR	+	+	+	+	-	-
EQQNAFYEILHLPNLTEEQR	+	+	-	-	-	-
EQQNAFYEILNMPNLNEEQR	+	+	+	+	-	-
FNKEQQNAFYEILHLPNLNEEQR	+	+	-	-	-	-
KLNESQAPK	+	+	-	-	-	-
KLNESQAPKADNKFNK	+	+	-	+	-	-
KLNESQAPKADNNFNK	+	+	-	+	-	-
LNDAQAPKADNKFNK	+	+	-	-	-	-
LNDAQAPKEEDNNKPGK	+	+	-	-	-	-
LNDSQAPK	+	+	-	-	-	-
LNESQAPKADNKFNK	+	+	-	-	-	-
LNESQAPKADNNFNK	+	+	+	+	-	-
NGFIQSLK	+	+	-	+	-	-
NGFIQSLKDDPSQSANLLAEAK	+	+	+	+	-	-
NGFIQSLKDDPSQSANLLAEAKK	+	+	+	+	-	-
NGFIQSLKDDPSQSANLLAEAKKLNDAQAPK	+	+	-	-	-	-
NGFIQSLKDDPSQSANLLSEAK	+	+	-	-	-	-
NGFIQSLKDDPSQSANVLGEAQK	+	+	+	+	-	-
NGFIQSLKDDPSQSANVLGEAQKLNDSQAPK	+	+	-	-	-	-
NGFIQSLKDDPSQSTNVLGEAK	+	+	+	+	-	-
NGFIQSLKDDPSQSTNVLGEAKK	+	+	-	-	-	-
NGFIQSLKDDPSQSTNVLGEAKKLNESQAPK	+	+	-	-	-	-
NGFIQSLKDDPSVSK	+	+	+	+	-	-
NGFIQSLKDDPSVSKEILAEAK	+	+	-	-	-	-
NGFIQSLKDDPSVSKEILAEAKK	+	+	-	-	-	-
KLNDAQAPK	-	+	-	-	-	-
KLNDAQAPKADNKFNK	-	-	-	+	-	-
NGFIQSLKDDPSQSANLLSEAKK	-	+	-	-	-	-

F

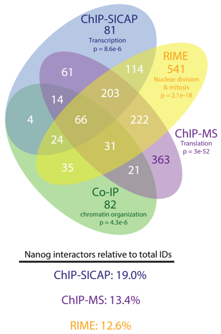

Figure S3

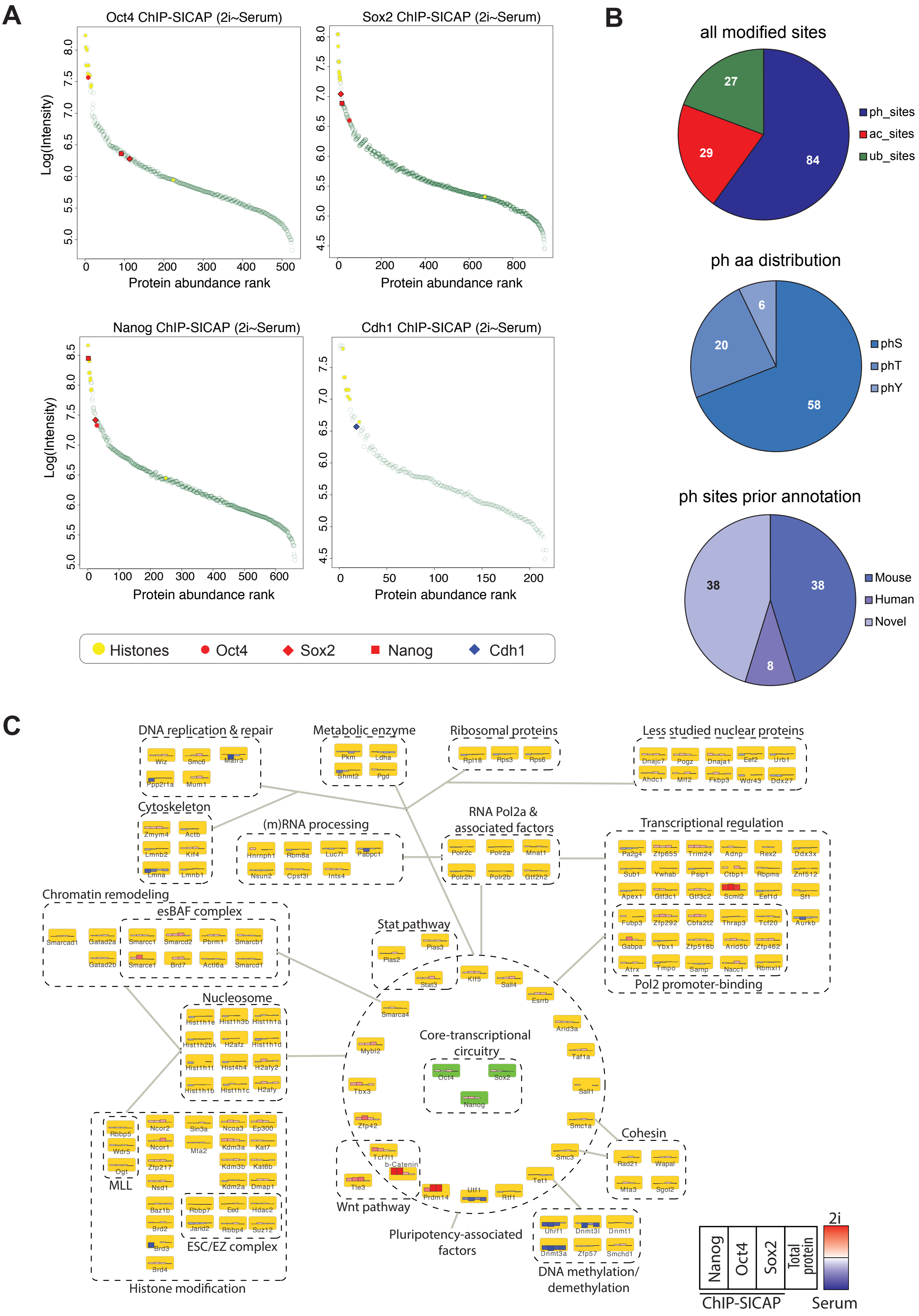

A
Oct4 ChIP-SICAP (2i~Serum)
Sox2 ChIP-SICAP (2i~Serum)
Nanog ChIP-SICAP (2i~Serum)
Cdh1 ChIP-SICAP (2i~Serum)
Log(Intensity)
Protein abundance rank
Histones
Oct4
Sox2
Nanog
Cdh1
B
all modified sites
27
29
84
ph_sites
ac_sites
ub_sites
ph aa distribution
6
20
58
phS
phT
phY
ph sites prior annotation
38
38
8
Mouse
Human
Novel
C
DNA replication & repair
Metabolic enzyme
Ribosomal proteins
Less studied nuclear proteins
Cytoskeleton
(m)RNA processing
RNA Pol2a & associated factors
Transcriptional regulation
Chromatin remodeling
esBAF complex
Stat pathway
Pol2 promoter-binding
Nucleosome
Core-transcriptional circuitry
Cohesin
MLL
Wnt pathway
Pluripotency-associated factors
ESC/EZ complex
Histone modification
DNA methylation/ demethylation
Nanog
Oct4
Sox2
Total protein
ChIP-SICAP
2i
Serum

Figure S4

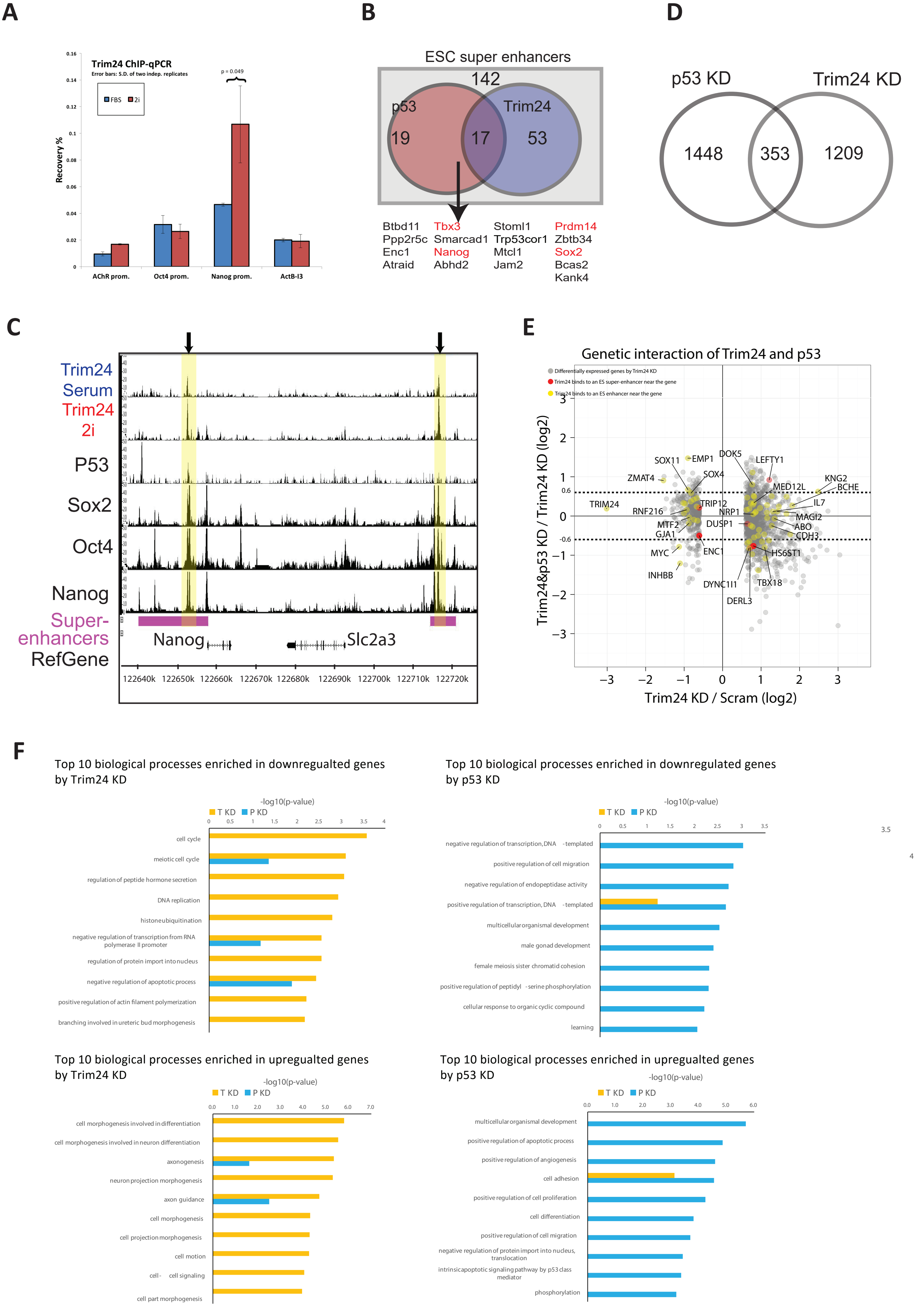

A
B
D
Trim24 ChIP-qPCR
Error bars: S.D. of two indep. replicates
FBS
2i
p = 0.049
Recovery %
AChR prom.
Oct4 prom.
Nanog prom.
ActB-I3
ESC super enhancers
142
p53
Trim24
19
17
53
Btbd11
Ppp2r5c
Enc1
Atraid
Tbx3
Smarcad1
Nanog
Abhd2
Stoml1
Trp53cor1
Mtcl1
Jam2
Prdm14
Zbtb34
Sox2
Bcas2
Kank4
p53 KD
Trim24 KD
1448
353
1209
C
E
Trim24
Serum
Trim24
2i
P53
Sox2
Oct4
Nanog
Super-
enhancers
RefGene
Nanog
Slc2a3
122640k
122650k
122660k
122670k
122680k
122690k
122700k
122710k
122720k
Genetic interaction of Trim24 and p53
Differentially expressed genes by Trim24 KD
Trim24 binds to an ES super-enhancer near the gene
Trim24 binds to an ES enhancer near the gene
Trim24&p53 KD / Trim24 KD (log2)
Trim24 KD / Scram (log2)
SOX11
EMP1
DOK5
LEFTY1
ZMAT4
SOX4
KNG2
MED12L
BCHE
TRIM24
TRIP12
IL7
RNF216
NRP1
MAGI2
MTF2
DUSP1
ABO
GJA1
CDH3
MYC
ENC1
HS6ST1
INHBB
DYNC1I1
TBX18
DERL3
F
Top 10 biological processes enriched in downregualted genes by Trim24 KD
-log10(p-value)
T KD
P KD
cell cycle
meiotic cell cycle
regulation of peptide hormone secretion
DNA replication
histone ubiquitination
negative regulation of transcription from RNA polymerase II promoter
regulation of protein import into nucleus
negative regulation of apoptotic process
positive regulation of actin filament polymerization
branching involved in ureteric bud morphogenesis
Top 10 biological processes enriched in downregulated genes by p53 KD
negative regulation of transcription, DNA -templated
positive regulation of cell migration
negative regulation of endopeptidase activity
positive regulation of transcription, DNA -templated
multicellular organismal development
male gonad development
female meiosis sister chromatid cohesion
positive regulation of peptidyl -serine phosphorylation
cellular response to organic cyclic compound
learning
Top 10 biological processes enriched in upregualted genes by Trim24 KD
cell morphogenesis involved in differentiation
cell morphogenesis involved in neuron differentiation
axonogenesis
neuron projection morphogenesis
axon guidance
cell morphogenesis
cell projection morphogenesis
cell motion
cell- cell signaling
cell part morphogenesis
Top 10 biological processes enriched in upregualted genes by p53 KD
multicellular organismal development
positive regulation of apoptotic process
positive regulation of angiogenesis
cell adhesion
positive regulation of cell proliferation
cell differentiation
positive regulation of cell migration
negative regulation of protein import into nucleus, translocation
intrinsic apoptotic signaling pathway by p53 class mediator
phosphorylation

Figure S5

Image of the plate by regular scanner

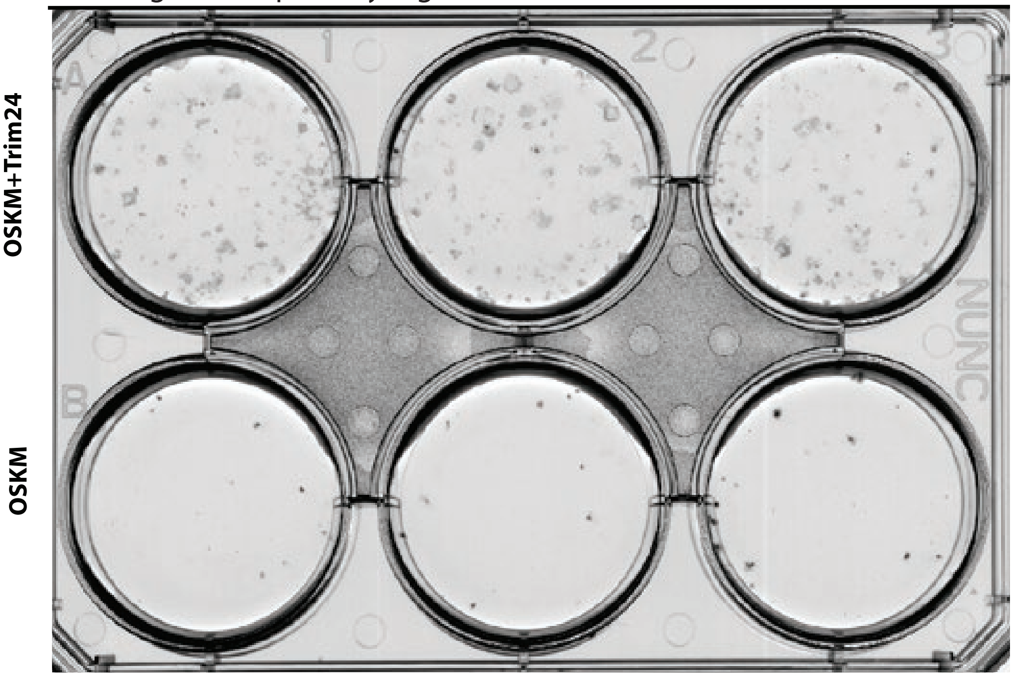

Stitched images in EGFP channel

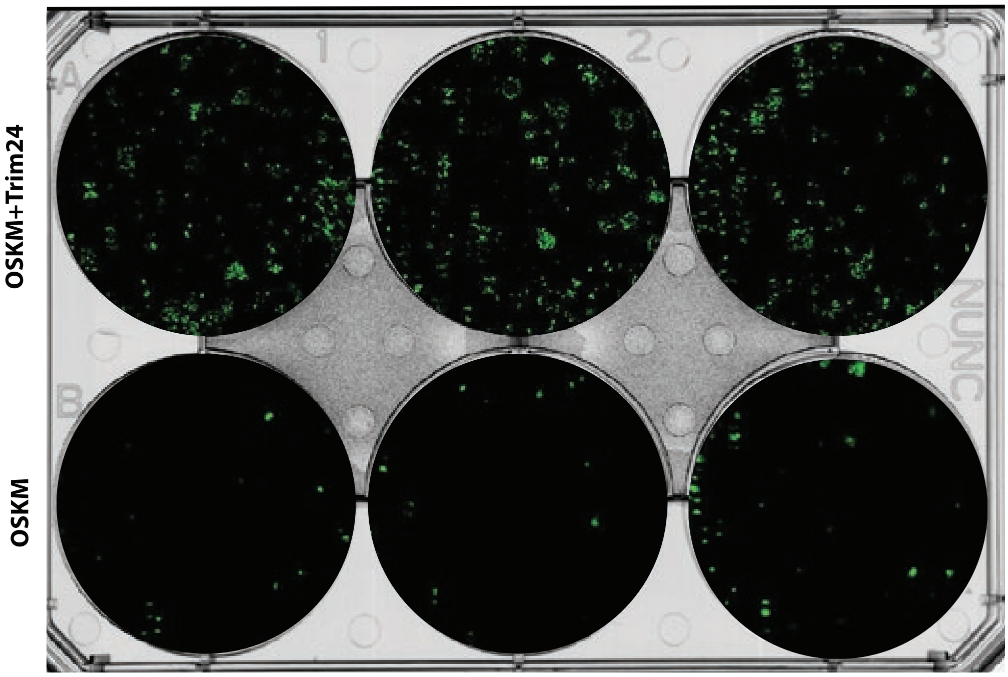

Figure S6

A

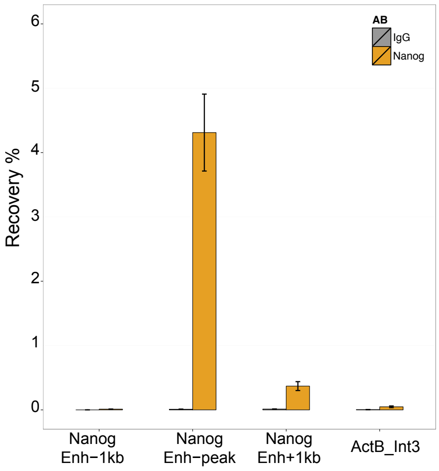

B

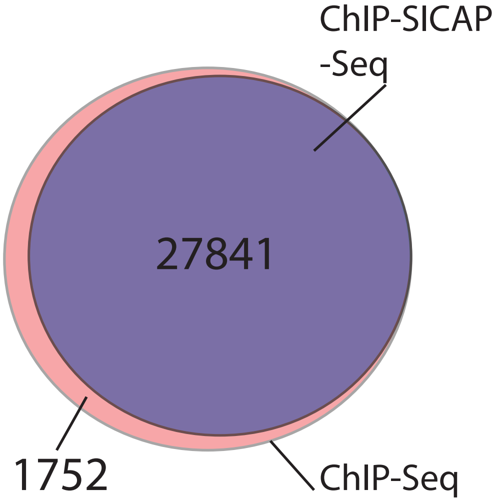

C

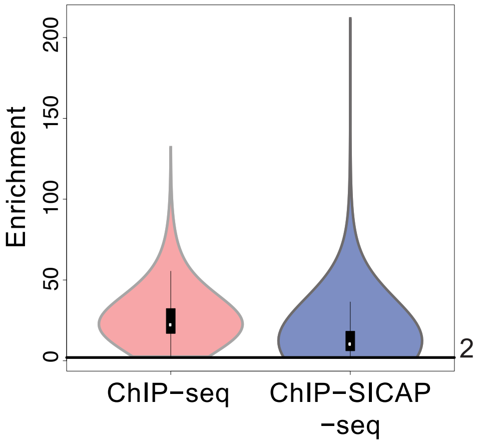

Supplemental Figure Legends

Figure S1. Experimental characteristics of ChIP-SICAP, ChIP-MS and RIME

Related to Figure 1

Highlighting the advantage of ChIP-SICAP to enrich for proteins co-localizing with a bait-protein on DNA while depleting for contaminating proteins.

Figure S2. Applying ChIP-SICAP, ChIP-MS and RIME using Nanog and no-antibody control in mouse ES cells

Related to Figure 2

(A) The scatterplots show the fold-enrichment of proteins using a Nanog antibody over a no-antibody control. (B) Venn diagrams showing the overlap between the replicates (numbers in green and grey zones), and the number of the enriched proteins using a 2-fold cut-off (numbers in green colored zone) using ChIP-SICAP, ChIP-MS and RIME. (C) Top-5 enriched GO biological processes for each method. (D) Identified peptides originating from antibody contaminating used for immuno-precipitation. + and – indicate whether or not the peptide was detected in the respective methods. (E) As in D, for peptides originating from proteinA contamination. (F) Venn diagram showing the absolute number of Nanog-interactors (as previously determined by CoIP) identified by each method. The number of Nanog interactors relative to the total number of identified proteins is shown below the Venn diagram. The top-enriched GO biological processes for the non-overlapping proteins are shown in the Venn diagram.

Figure S3. Comparative ChIP-SICAP and full proteome between 2iL and serum

Related to Figure 3.

(A) Abundance-ranked proteins identified by ChIP-SICAP using Oct4 (top-left), Sox2 (top-right), Nanog (bottom-left) and Cdh1 (bottom-right) as bait proteins. (B) Distribution of PTMs identified (ph, ac and ub correspond to phosphorylation, acetylation and ubiquitination, respectively) (top panel), distribution of phosphorylated sites at serine, threonine and tyrosine (middle panel); and grouping of the identified phosphorylated sites indicating whether they had been previously described in mouse, human proteins (bottom panel). (C) Network visualization of the proteins identified by OSN SICAP with significant changes (adj. p-value <0.1 in at least one of the OSN ChIP-SICAP assays). For each protein, the first three bars indicate the protein ratio in 2iL/serum obtained by SICAP using antibodies for Oct4, Sox2 and Nanog, respectively. The fourth bar shows the protein ratio in the total proteome between 2iL and serum conditions.

Figure S4. Validation of Trim24 by ChIP-seq and the effect of Trim24 knock down

Related to Figure 4.

(A) The bar chart indicates the binding of Trim24 to Nanog promoter preferentially in 2iL media. Prom.: promoter <1kb from the TSS. I3: Intorn 3. Error bars indicate standard deviations of two independent replicates. (B) The Venn diagram shows the super-enhancers encompassing Trim24 (this study) and p53-binding sites (Li et al., 2012). The genes in the vicinity of the overlapping super-enhancers are shown, including the most critical pluripotency genes shown in red. (C) The traces show the ChIP-seq profiles of Trim24 (in 2iL and serum), p53, Oct4, Sox2 and Nanog near the Nanog locus. Aforementioned proteins co-localize on the super-enhancer upstream of the

Nanog locus (yellow highlights). (D) Overlap of differentially expressed gene sets found after Trim24 knockdown and p53 knockdown. (E) Scatterplot showing the genes differentially expressed after Trim24 knockdown, and their co-regulation by p53 (as in Fig 5a). Red and yellow dots indicate situations where Trim24 binds to a super-enhancer or to a non-super-enhancer near the gene, respectively. (F) Top-10 enriched GO terms of differentially expressed genes after Trim24 and p53 knockdown (left and right column, respectively) among the down-regulated (top row) and the up-regulated (bottom row) genes.

Figure S5. The effect of Trim24 on the efficiency of reprogramming

Related to Figure 5

Top, a surface scan of the plate was taken. Bottom, the stitched images in EGFP channel are shown for each well. OSKM: induction of Oct4, Sox2, Klf4 and c-Myc. To see the details of the colonies please refer to Table S6.

Figure S6. **Recovering DNA after ChIP-SICAP for Nanog**

Related to Figure 6

(A) qPCR after ChIP-SICAP, showing the enrichment of Nanog at the Nanog enhancer compared to regions ± 1 kb away. ActB_Int3: Intron 3 of ActB. Error bars indicate standard deviations of 2 independent replicates. (B) Venn diagram comparing the overlap in peaks detected by ChIP-seq after recovery of DNA via regular ChIP-seq (red) and ChIP-SICAP-seq procedures (blue). (C)

Violinplot indicating the density of the enriched peaks after recovery of DNA via regular ChIP-seq (red) and ChIP-SICAP-seq procedures (blue).

Supplemental Tables

Table S1_Comparing SICAP, ChIP-MS, RIME and full proteome mass spec, related to Figure 2 and Figure S2

Table S2_Comparative ChIP-SICAP between 2i and serum, related to Figures 3 and 4A

Table S3_Trim24 ChIP-seq, related to Figure 4B and 4C

Table S4_gene expression after Trim24 knockdown, combined with ChIP-seq peaks, related to Figures 4D and 5A

Table S5_Trim24 and iPS generation-colony counting, related to figure 5B

Table S6. Comparing Nanog ChIP-seq and ChIP-SICAP-Seq, related to Figure 6

Supplemental Experimental Procedures

Detailed ChIP-SICAP protocol

Required materials

- Formaldehyde (Methanol-free, Pierce 28906, or 28908)
- IP buffer: Triton 1%, NP40 0.5%, Tris.Cl pH= 7.5-8 50mM, EDTA 5mM, NaCl 150mM
- BW2x buffer : Tris.Cl pH = 8.0, 10mM, EDTA = 1mM., 0.1% TritoneX100, NaCl 2000mM
- Elution buffer: SDS 7.5% + DTT 200mM in H2O
- SDS wash buffer: Tris.Cl 10mM, EDTA 1mM, SDS 1%, NaCl 200mM
- 2-propanol wash buffer: 2-propanol 20% in water
- Acetonitril wash buffer: Acetonitril 40% in water
- Complete protease inhibitor (PI) 50x (Roche app.): dissolve 1 tablet in 1ml ddH2O
- TE buffer: Tris-Cl(10mM, pH~ 7.5), EDTA (1mM)
- Tris buffer: Tris-Cl(10mM, pH~ 7.5)
- TdT (Fermentas, Thermo Scientific, EP0162)
- Biotin-ddUTP (Jenabioscience, NU-1619-BIOX-S)
- Streptavidin Magnetic Beads (NEB, S1420S)
- StemPro Accutase (A1110501, Thermofisher, Life tech)
- Dynabead protein-A or protein-G (Thermofisher, Life tech)
- Sera-Mag magnetic beads (65152105050250 and 45152105050250), GE Healthcare
- Agencourt AmpureXP beads (Beckmancoulter)
- Digestion buffer: Ammonium bicarbonate 50mM, SDS 0.1%

Experimental procedure:

1- Detach the cells by Accutase or any other method for splitting the cells. Then count the cells.

2- Spin the cells 1000g, 5min. Then discard the media.

3- Cross-link DNA-protein complexes by resuspending the cells in PBS + formaldehyde (1.5% v/v final conc.). For every 10 million cells, add 10ml of the formaldehyde solution.
   - The cells should be completely resuspended.
4- Invert the tubes several times within 14min.
5- Add Glycine (fincal conc. 120mM) to stop the reaction. Again Invert the tubes several times within 5min.
6- Spin the cells at 2000g, 2min. Then discard the liquid.
7- Resuspend the cells with PBS in max. vol. of the tube
8- Spin the cells at 2000g, 2min. Then discard the liquid.
9- Resuspend the cells with PBS, and count the cells again.
10- Pour 24 million cells in a 15-ml tube, and spin the cells at 2000g, 2 min
11- For each replicate you may need 24 million cells (or at least 8 million).
   - The negative control for this assay is a No-antibody or normal IgG. The best negative control is knockout control, which is not always available. No-biotin control is not needed, as it is always super clean.
   - After cell pellets could be frozen at stored -80 for months.

12- Mix TE buffer (10mM, pH~ 7.5) + Complete protease inhib. (final conc. is 2x).
13- Resuspend the cells in 1ml of TE vortexing, and transfer them into a 2-ml micro-tube
   - If you are going to compare 2 cellular states that are labeled by SILAC (e.g. 2iL and serum state of mouse ES cells) you may mix the cells from the beginning.
   - If you have a target of interest, and you wish to compare it with the negative control, you may mix the samples after the end-labeling of DNA, as it is mentioned subsequently.
14- Spin 1000g, 2min
15- Discard the supernatant.
16- Resuspend again by vortexing in 0.9ml of TE buffer
17- Put the micro-tubes for 10 min on ice
18- Add 0.1ml TritoneX100 10% (the final conc. is 1%), vortex, and keep 5min on ice
19- Spin 1000g, 2min
20- Discard supernatant

21- Resuspend again by vortexing in 1ml TE+ Complete PI (2x),
22- Spin 1000g, 2min
23- Discard supernatant
24- Resuspend again by vortexing in 1ml TE+ Complete PI (2x),
25- Spin 500g, 2min
26- Resuspend each 24million cells in ~700ul TE+ Complete PI (2x)
    - the final vol. should be ~780ul, so then each 24million cells is divided into 6x130ul to be sonicated.
27- Pour 130ul of the cell suspension in a Covaris micro-TUBE
28- Sonication for mouse ES cells using Covaris S220 was carried out as follows:
    - Time: 430s, Duty cycle: 10%, Intensity: 5, Cycle/Burst: 200
    - Depending on the cells, the sonication should be optimized to achieve the sheared chromatin fragments with the desired distribution (usually 200-500bp).
29- After the sonication, collect the liquid, and pour it into a 2-ml microtube
30- Repeat the sonication for the next 130ul.
    - Don't use a Covaris microTUBE more than 2 times.
    - Don't forget to resuspend the cells for the next run
    - Don't generate too much bubble in the sonication tube, specially in the middle of the solution (having a bubble on top is okay)
31- Pool 6x130ul in one 2-ml microtube. Now you have sheared chromatin from the original 24million cells.
32- After the sonication spin the tubes 10min at 12000g
33- Collect the supernatant, (it should be ~600-700ul)
34- You may keep 2% as the input for ChIP-Seq, also 20ul for checking the distribution of the sheared chromatin on gel.
35- Optional: you may add 10ul of RNase A to be sure the proteins are not cross-linked via RNA to DNA.
36- Add the following reagents to the sheared chromatin in each microtube:
    - Add 24 ul NaCl 5M (final conc. 150mM)
    - Add 80 ul Tritone X100 10% (final conc. 1%)
    - Add 40 ul NP40 10% (final conc. 0.5%)
    - Vortex gently to homogenize the reagents completely

- Add a proper amount of the antibody. (Normally between 1-5 ug depending on the antibody)
    i. You may include a no-antibody control, or normal IgG control
- Fill it with TE up to ~800ul
- Vortex slightly, and gently

37- Shake overnight in a Thermomixer (Eppendorf) at 4°C with 800 RPM agitation.
38- The day after that, spin the tubes at 12000g, 10min
39- Collect the supernatant (~790ul), pour it in a 2-ml microtube
40- Add 700ul IP buffer into each of the microtubes
41- Wash the magnetic Dynabeads (protein G or protein A depending on the antibody) with IP buffer
42- Add 30ul magnetic Dynabeads
43- Rotate the samples 2-4 hours at 4°C head to tail, at 20 RPM,
44- Put the tubes on the magnet, after 2min remove the liquid, and take the tubes off the magnet
45- Wash the beads by 1000ul Tris-HCl 100mM pH= 7.5 <u>(No EDTA)</u>
    - Each washing consists of resuspending the beads in the washing solution by inverting/rotating the tubes (<u>Don't vortex</u>), briefly spinning, and putting on the magnet for 2 min to remove the solution
46- Resuspend the beads in 100ul TdT buffer 1x, and keep 5min at RT. <u>Don't pipet the</u> beads, just gently swirl the tubes.
47- Put the tubes on the magnet, after 1min remove the liquid, and take the tubes off the magnet
    - be careful not to dry the beads
48- Resuspend the beads in 93ul TdT buffer 1x
    - +5ul ddUTP-Biotin (1mM stock)
    - +2ul TdT
49- Incubate 30min in a thermomixer at 37°C, with 500 RPM agitation
    - The no-antibody (or IgG control) should also be treated with TdT and biotinylated nucleotide to estimate background chromatin contamination.
50- Wash the beads 6 times with 1ml ice-cold IP buffer at room temp.
51- Resuspend the beads in 100ul of the elution buffer by vortexing

52- Incubate 15min at 37, with 750 RPM agitation
53- Collect the supernatant, and discard the beads
54- If you are comparing one target with the negative control, and you have SILAC labeled proteins, then mix the content of the two tubes (2x100ul). Therefore, at this step you have 1 tube per replicate
55- Add 1300ul IP buffer
56- Add 50ul Streptavidin magnetic beads to each tube
57- Rotate 1 hour at RT
58- Put the tubes on the magnet after 2 min discard the solution.
59- Wash the beads 3 times by SDS wash buffer
    - Each washing consists of resuspending the beads in the washing solution by vortexing, briefly spinning, and putting on the magnet for 2 min to remove the solution
60- Wash once with BW2x buffer
61- Wash the beads with Iso-propanol wash buffer, 2 times
62- Wash the beads with acetonitrile wash buffer, 4 times
63- Resuspend the beads in 80ul acetonitrile wash buffer, and transfer it into a 0.2-ml PCR tubes.
64- To be sure that all the beads have been transferred, pour 80ul acetonitrile wash buffer into the 2-ml tube and transfer it to the PCR tube.
65- Put the tubes on the magnet, and remove the wash buffer.
66- Resuspend in 14ul digestion buffer
67- Add 1ul DTT 100mM,
68- Incubate 95 °C, 20min
69- Once the tube is cool enough, add 1ul IAA 200mM, 60min at RT in a dark place
70- Add 1ul DTT 100mM,
71- Remove the beads, and transfer the liquid into a new tube.
72- Add 200ng Trypsin and 50ng LysC
73- Incubate at 37°C for 14 hours

Clean up the peptides by SP3 protocol (Hughes et al., 2014)This protocol removes SDS and other detergents:

1- Prepare the SP3 beads by mixing 2x20ul of Sera-mag beads (65152105050250 and 45152105050250) in a 0.2-ml PCR tube

2- Add 160ul of deionized H2O, vortex and put the tube on the magnet
3- Discard the liquid, and resuspend the beads in 200ul of dH2O
4- vortex and put the tube on the magnet
5- repeat step3 and 4 two times more.
6- Resuspend the beads in 100ul of dH2O. The beads could stored 2 weeks in a fridge.
7- Add 2ul of the SP3 beads to the peptides to be cleaned up, and vortex
8- Add 200ul ACN 100% and vortex
9- Spin briefly
10- Add 20ul more ACN 100% to each PCR tube, and close the caps.
11- Wait 10min
12- Put the tubes on the magnet for 1-2min
13- Take the liquid but don't discard it! It contains DNA, which can be used for qPCR or sequencing after DNA-purification.
14- Pour it into a 1.5ml microtube.
15- Without vortexing and disturbing the SP3 beads, pour 200ul of ACN 100% and wait a few seconds
16- Discard the liquid.
17- Take the tubes off, and spin briefly
18- Discard the residues of the ACN
19- Add 10ul of DMSO 2% on wall of the tube, don't pipette
20- Vortex the tube vigorously, and spin it for a few seconds
21- Sonicate the tubes in water bath for 5min
22- Put the tubes on the magnet
23- In new PCR tubes, add 1ul of formic acid 1%
24- Take the liquid and pour it into the new PCR tubes
25- Take 10ul and pour it into the glass insert or another PCR tube to be injected to the mass spec.

Recovering DNA after digesting the proteins:

1- Dry out the ACN fraction, obtained in the step 13, using a speedvac
2- Reconstitute the DNA in 50ul of TE buffer.
3- Add 100ul of Ampure XP beads, vortex, spin, and wait 10min
4- Put the samples on a magnet, and wait a few min
5- Discard the liquid, and add 200ul of freshly prepared 70% Ethanol

6- Repeat the last step
7- Spin briefly
8- Put the samples on a magnet, and discard the residues of Ethanol
9- Resuspend the beads in 20ul of Tris-Cl 10mM without EDTA.
10- The DNA could be directly used for library preparation, or qPCR.
11- The input control could be prepared with the same protocol, however, cross-linking should be reversed and proteins should be digested by Trypsin or proteinase K, ideally in parallel to the other samples.

ChIP-MS and RIME

ChIP-MS procedure was as same as SICAP, however omitting TdT-mediated end-labeling of DNA with biotin. Therefore, following 6 washing steps by IP buffer, the proteins were reverse cross-linked by SDS-PAGE loading buffer. After running the SDS-PAGE for 2-3cm, each lane was divided into 3 pieces and the proteins were digested in-gel by trypsin (Shevchenko et al., 2006).

RIME was essentially carried out as described in the original paper (Mohammed et al., 2013), and is similar to ChIP-MS (above) with the difference that proteins are digested on-bead. The amount of the antibody and the number of cells were the same as ChIP-SICAP. After digestion and clean-up RIME samples were subjected to High pH peptide fractionation.

High pH fractionation and mass spectrometry

Following digestion of the proteins and acidification to remove RapiGest, the peptides were cleaned using stage-tipping procedure (Rappsilber et al., 2007). Then the samples were subjected to fractionation using high pH reverse-phase chromatography. Peptides were fractionated on an Agilent 1200 Infinity HPLC system with a Gemini C18 column (3 μm, 110

Å, 100 × 1.0 mm, Phenomenex) using a linear 60 min gradient from 0% to 35% (v/v) acetonitrile in 20 mM ammonium formate (pH 10) at a flow rate of 0.1 ml/min. Elution of peptides was detected with a variable wavelength UV detector set to 254 nm. Thirty-two 1-min fractions were collected that were subsequently pooled into ten fractions. Each fraction was then analyzed using LC-MS on a Orbitrap Velos Pro mass spectrometer (Thermo Fisher Scientific) connected to a nanoAcquity UPLC (Waters) via a nanoelectrospray ion source (Thermo). Peptides were separated with a BEH300C18 (75 μmx 250 mm, 1.7 μm) UPLC column (Waters) using a stepwise 60-min, from 3% to 85% (v/v) acetonitrile in 0.1% (v/v) formic acid at a flow rate of 300 nl/min. The LTQ-Orbitrap Velos Pro mass spectrometer was operated in data-dependent mode, acquiring one survey MS scan in the orbitrap followed by up to 15 fragmentation scans (TOP15) of the most abundant ions analyzed in the LTQ by CID fragmentation. Only charge states of two and higher were allowed for fragmentation. Essential MS settings were: full MS: AGC = 10E6, maximum ion time = 500 ms, m/z range = 375–1600, resolution = 30 000 FWHM; MS2: AGC = 30 000, maximum ion time = 50 ms, minimum signal threshold = 1500, dynamic exclusion time = 30 s, isolation width = 2 Da, normalized collision energy = 40, activation Q = 0.25.

Processing mass spectrometry data

The MS spectra were analyzed using Proteome Discoverer 1.4 (Thermo Fisher Scientific), and the proteins were identified using MASCOT search engine (Matrix Science) against the Mus musculus proteome of the Uniprot database. Searches were carried out based on tryptic specificity. The precursor and ms/ms tolerance were set on 20 ppm and 0.5 Da, respectively. Carbamidomethylation of Cytosine was selected as a fixed modification, and as dynamic modifications, oxidation of methionine and N-terminal acetylation were selected in addition

to the SILAC labels (Arg 10, and Lys 8). The Perculator algorithm (Kall et al., 2007) was used to limit FDR rates to a q-value<0.01. Proteins were identified and quantified by at least 1 unique peptide. For subsequent analysis, protein grouping was enabled to consider only the master proteins.

In comparison between Nanog and No-antibody control, the option for "replace the missing values with minimum quantity" in proteome discoverer was used. Because most of the proteins were over-represented in the Nanog samples. No normalization was applied in this analysis. Proteins identified with both replicates, and with Nanog over no-antibody ratios > 2-fold were considered as enriched proteins for subsequent analyses.

In comparison between 2iL and serum, the distributions of the ratios were normalized by the median. Then, the analysis was carried out without "replace the missing values with minimum quantity". For a few protein groups no ratio was reported due to the signals only from one of the channels. In these cases, the analysis was repeated using "replace the missing values with minimum quantity". Then the maximum ratio was limited to 32-fold, which was reported for these protein groups. To identify differentially expressed/bound proteins, we applied t-test using Limma package (Ritchie et al., 2015), then the p-values were adjusted using Benjamini-Hochberg method (adj. p-value). We considered adj. p-values <0.10 as significant changes.

For the analysis of post-translational modification, the acquired Raw data were analyzed by the integrated MaxQuant software v.1.5.2.8, using the Andromeda search engine (Cox, J., and Mann, M. 2008, Cox, J., et al 2009). The MOUSE 1401 database (51195 entries) was used for peptide identification. Enzyme specificity was set to trypsin, estimated peptide false discovery rate (FDR) identification to 1%, a maximum of 3 missed cleavages were permitted and the minimum peptide length was fixed to 5 amino acids.

Different MaxQuant jobs were carried out in order to include as variable modifications: mono- (+14.016 Da) and di-methylation (+28.031 Da) of lysine and arginine, acetylation of lysine (+42.010 Da), ubiquitination of lysine (+114.043 Da), phosphorylation of serine, threonine and tyrosine (+79.966 Da), and oxidation of methionine (+15.995 Da). Only peptides with evidence score higher than 60 (Cox, J., et al 2011) and localization probability score greater than 0.75 (Olsen, J. V., et al 2006, Monetti, M., et al 2011, Pines, A., et al 2011) were accepted. For each experiment, evidence SILAC ratios were normalized on the corresponding protein SILAC ratio, to define peptide trends.

ChIP-seq

Shearing chromatin and IP were performed as described above for ChIP-SICAP, however omitting the DNA-labeling. In addition, an aliquot was taken from the clear sheared chromatin to be used as an input control. After the IP and six rounds of washing steps with the ice-cold IP buffer, the beads were resuspended in TE buffer plus 1% of SDS. Then the samples heated at 95°C for 20min, and 40μg proteinase K was added for protein digestion at 55°C for 30min. Next DNA was purified using phenol/chloroform isoamyl alcohol and precipitated using glycogen and ethanol. Finally, DNA was resuspended in 30μl of Tris-HCl 10mM.

To prepare the library for Illumina sequencing, purified ChIP DNA was end-repaired by Klenow, T4 DNA polymerase and T4 polynucleotide kinase. Then DNA fragments were subjected to A-tailing, and NEBNext adapter ligation (NEB Index Primers Set 1, E7335S). Following PCR for 12 cycles, the amplicons were size-selected by mixing 50μl PCR products with 30μl of Ampure XP beads. The supernatant was collected, and again 45 μl of AmpureXP beads was added. After 2 rounds of washing with 70% ethanol, the DNA was

eluted in 50µl of Tris-HCl 10mM. Once again the eluted DNA was mixed with 48µl of AmpureXP beads, and after the washing, they were eluted by 15 µl of Tris-HCl 10mM. Sequencing was carried out by Illumina HiSeq 2000 according to the manufacturer's protocols.

ChIP-seq data analysis

Unless stated otherwise, analysis was performed in a local installation of Galaxy (Blankenberg et al., 2010; Giardine et al., 2005; Goecks et al., 2010) maintained by the EMBL Genome Biology Computational Support. The 50-bp single-end reads were aligned to build version NCBI37/MM9 of the mouse genome using Bowtie version 2(Langmead et al., 2009) using standard options (Galaxy Tool version 0.2, sensitive preset). Reads failing to be mapped or mapping at several locations (as identified by the XS tag set by bowtie2) were removed using the "Filter SAM" tool and the "Select" tool, respectively. Read duplicates where identified and removed using Picard's Mark Duplicates (http://broadinstitute.github.io/picard). Sequencing data quality was assessed using FastQC and the Deeptools package (Ramirez et al., 2014). ChIP quality was estimated by cross-correlation using the "SPP" tool as suggested by ENCODE ChIP-seq guidelines (Landt et al., 2012). Finally, reproducibility of ChIP replicates and final peak selection was achieved using the IDR pipeline depicted in Fig 7D of Landt et. al. (Landt et al., 2012), and implemented following instructions from https://sites.google.com/site/anshulkundaje/projects/idr. We used MACS version 2 (Zhang et al, 2008) as the underlying peak caller with recommended options (i.e. setting p-value cutoff to 1e-3). The final list of peaks used in the study corresponds to the "optimal" list (i.e. using peak called on merged replicates) at a 2% IDR cutoff. Heatmaps were produced using the Deeptools "compute-Matrix" and "heatmapper" tools on input-

subtracted coverage files (see below). Coverage files (bigwig format) were generated using the Deeptools "bam-Coverage" tool using the "Normalize coverage to 1x" option. Input subtraction (from ChIP signal) was performed using the Deeptools "bigwig-Compare" tool. An average fragment size of 200 bp and bin sizes of 50 bp was systematically used.

RNA-seq data analysis

The 84-bp single-end reads were aligned to build version NCBI37/MM9 of the mouse genome using STAR (Galaxy Tool RNA STAR Version 2.4.0d-2) and standard options. Count tables were generated at the gene level using htseq-count (Galaxy Tool Version 0.6.1galaxy1, with --mode union --minaqual 10 --stranded reverse and other defaults) using ENSEMBL genome annotations version 37.67 (Mus_musculus.NCBIM37.67.gtf). Finally, differentially expressed genes for the different contrasts were called with DESeq2 (Galaxy Tool DESeq2 Version 2.1.8.3).

List of the antibodies used for the IP

Nanog (D2A3) XP Rabbit mAb (Cell Signaling Tech, 8822 S) was applied 1:100 (v/v) to the sheared chromatin obtained from 24 million cells for the ChIP-SICAP assay.

Oct-4A (C30A3C1) Rabbit mAb (Cell Signaling Tech, 5677 S) was applied 1:50 (v/v) to the sheared chromatin obtained from 24 million cells for the ChIP-SICAP assay.

Human SOX2 Affinity Purified Polyclonal Ab (R&D Systems, AF2018) 25µg was applied to the sheared chromatin obtained from 24 million cells the ChIP-SICAP assay.

E-Cadherin (24E10) Rabbit mAb (Cell Signaling Tech, 3195s) was applied 1:50 (v/v) to the sheared chromatin obtained from 24 million cells for the ChIP-SICAP assay.

Trim24/TIF1a antibody (Bethyl lab, A300-815A) 2.5 μg was applied to the sheared chromatin obtained from 24 million cells for the ChIP-Seq assay.

GO analysis and Cytoscape plugins

Annotations of the genes were determined either using Perseus software (Cox and Mann, 2008), or were downloaded directly from Uniprot database. GO biological processes and their enrichments were determined using DAVID bioinformatics resources (Huang da et al., 2009). Cytoscape (Shannon et al., 2003) version 3.2.1 was used to apply enhancedGraphics (Morris et al., 2014).

Transduction of MEF cells with Trim24, iPS generation and microscopy

Trim24 cDNA was amplified by PCR using the Forward primer: 5’-AATGGAGGTGGCTGTGGAGAA-3’ and the reverse primer: 5’-tttggatCCAGTGCGGCGTTACTTAA-3’. The reverse primer contained the BamHI site. The PCR was carried out by Phusion polymerase to obtain blunt ends. Then the products were digested by BamHI, and the 5’-ends were phosphorylated by polynucleotide kinase (PNK). FU-tetO-hcmyc (Addgene plasmid # 19775) was digested by XbaI, and fill in by Klenow to make one end blunt. Then the vector was digested by BamHI, followed by dephosphorylation of the 5’-end by CIP and ligation to the vector. The correct coding-sequencing was confirmed by sanger sequencing. Subsequently HEK293T cells were transfected by pFU-tetO-Trim24, psPAX2, and VSV-G as described previously (Hansson et al., 2012). Briefly 8 million cells

were seeded in a T75 flask, after an overnight incubation the cells were transfected using FuGENE HD. After 8 hours the media of the cells was changed with fresh media. About 2 days after the transfection, the virus-containing media was collected, filtered, and concentrated using Amicon100kD ultrafiltration tubes. The concentrated media was used to infect 3 wells in a 6-well plate containing ~30000 reprogrammable MEFs (Stadtfeld et al., 2010) in each well. One day after the infection, the viral media was discarded and fresh media containing ~100000 feeder MEF cells were added to the wells. One day later, the cells were fed with ES media plus doxycycline (1 µg/ml) for the next two weeks. Then the cells were fixed by formaldehyde 4%, and ~1300 images were automatically taken from each well in EGFP channel to cover almost all surface of the wells (Figure S5). The images were stitched by grid collection stitching package (Preibisch et al., 2009) incorporated in Fiji. Then the number of Oct4-EGFP positive colonies were counted by “Analyze particle” in Fiji with minimum area of the colonies was set to be 250 (Table S6).

Transduction of the ES cells with shTrim24 and shP53

The knock down (KD) was carried out by validated Sigma-Alderich lentiviral vectors: non-targeting shRNA, shTrim24 (TRCN0000088518), shTrp53 (TRCN0000310844), and the mixture of the last two shRNAs. The lentiviruses were produced and concentrated as mentioned above. For each knockdown, 3 independent transductions were performed. The ES cells adapted in 2iL medium were infected with the viruses, and after 24 hours the viral media was replaced with the regular 2iL medium. Then 24 hours later, the cells were lysed, and RNA was extracted to be analyzed by qPCR and mRNA-seq. The RNA extraction and library preparation from poly(A)-RNA were carried out according to the standard Illumina TruSeq protocol.

Cell culture

46c mouse ES cells were grown feeder-free on 0.2% gelatinized cell culture plates in either traditional ES media with serum or 2iL-media. The serum media contained DMEM high glucose (Life technologies, 11965-092) supplemented with 15% fetal bovine serum (Life technologies, 10270-106), 100µM MEM non-essential aminoacids (Life technologies, 11140-050), 1x Glutamax (Life technologies, 35050-061), 1x penicillin and streptomycin (Life technologies, 15140-122), 100µM of 2-mercaptoethanol (Sigma, M7522), and 200ng/ml of LIF (EMBL, protein expression core facility). The 2i+LIF (2iL)-media contained DMEM/F12 media for SILAC (Pierce, 88215), 100µM MEM non-essential aminoacids (Life technologies, 11140-050), 1x Glutamax (Life technologies, 35050-061), 1x penicillin and streptomycin (Life technologies, 15140-122), 100µM of 2-mercaptoethanol (Sigma, M7522), 0.5mg/ml of BSA (Sigma, A3059), 200ng/ml of LIF (EMBL, protein expression core facility), 1µM of PD0325901 (Reagents Direct, 39-C68), 3µM of CHIR99021 (Reagents Direct, 27-H76). In addition, for light SILAC, 100 mg/ml of Lysine (L8662), 100 mg/ml of Arginine (Sigma, A6969) and 100 mg/ml of Proline (Sigma, P5607) were added to the 2i-media. For Heavy SILAC, 100 mg/ml of $^{13}C_6$,$^{15}N_2$-L-Lysine HCl (Silantes, 211604102), 100 mg/ml of $^{13}C_6$,$^{15}N_4$-L-Arginine HCl (Silantes, 201604102) and 100 mg/ml of Proline (Sigma, P5607) were added to the 2iL-media.

References

Blankenberg, D., Von Kuster, G., Coraor, N., Ananda, G., Lazarus, R., Mangan, M., Nekrutenko, A., and Taylor, J. (2010). Galaxy: a web-based genome analysis tool for experimentalists. Current protocols in molecular biology / edited by Frederick M Ausubel [et al] *Chapter 19*, Unit 19 10 11-21.

Cox, J., and Mann, M. (2008). MaxQuant enables high peptide identification rates, individualized p.p.b.-range mass accuracies and proteome-wide protein quantification. Nature biotechnology *26*, 1367-1372.

Giardine, B., Riemer, C., Hardison, R.C., Burhans, R., Elnitski, L., Shah, P., Zhang, Y., Blankenberg, D., Albert, I., Taylor, J*., et al.* (2005). Galaxy: a platform for interactive large-scale genome analysis. Genome research *15*, 1451-1455.

Goecks, J., Nekrutenko, A., Taylor, J., and Galaxy, T. (2010). Galaxy: a comprehensive approach for supporting accessible, reproducible, and transparent computational research in the life sciences. Genome biology *11*, R86.

Hansson, J., Rafiee, M.R., Reiland, S., Polo, J.M., Gehring, J., Okawa, S., Huber, W., Hochedlinger, K., and Krijgsveld, J. (2012). Highly coordinated proteome dynamics during reprogramming of somatic cells to pluripotency. Cell Rep *2*, 1579-1592.

Huang da, W., Sherman, B.T., and Lempicki, R.A. (2009). Systematic and integrative analysis of large gene lists using DAVID bioinformatics resources. Nature protocols *4*, 44-57.

Hughes, C.S., Foehr, S., Garfield, D.A., Furlong, E.E., Steinmetz, L.M., and Krijgsveld, J. (2014). Ultrasensitive proteome analysis using paramagnetic bead technology. Mol Syst Biol *10*, 757.

Kall, L., Canterbury, J.D., Weston, J., Noble, W.S., and MacCoss, M.J. (2007). Semi-supervised learning for peptide identification from shotgun proteomics datasets. Nature methods *4*, 923-925.

Landt, S.G., Marinov, G.K., Kundaje, A., Kheradpour, P., Pauli, F., Batzoglou, S., Bernstein, B.E., Bickel, P., Brown, J.B., Cayting, P*., et al.* (2012). ChIP-seq guidelines and practices of the ENCODE and modENCODE consortia. Genome research *22*, 1813-1831.

Langmead, B., Trapnell, C., Pop, M., and Salzberg, S.L. (2009). Ultrafast and memory-efficient alignment of short DNA sequences to the human genome. Genome biology *10*, R25.

Li, M., He, Y., Dubois, W., Wu, X., Shi, J., and Huang, J. (2012). Distinct regulatory mechanisms and functions for p53-activated and p53-repressed DNA damage response genes in embryonic stem cells. Mol Cell *46*, 30-42.

Mohammed, H., D'Santos, C., Serandour, A.A., Ali, H.R., Brown, G.D., Atkins, A., Rueda, O.M., Holmes, K.A., Theodorou, V., Robinson, J.L*., et al.* (2013). Endogenous purification reveals GREB1 as a key estrogen receptor regulatory factor. Cell reports *3*, 342-349. Montojo, J., Zuberi, K., Rodriguez, H., Kazi, F., Wright, G., Donaldson, S.L., Morris, Q., and Bader, G.D. (2010). GeneMANIA Cytoscape plugin: fast gene function predictions on the desktop. Bioinformatics *26*, 2927-2928.

Morris, J.H., Kuchinsky, A., Ferrin, T.E., and Pico, A.R. (2014). enhancedGraphics: a Cytoscape app for enhanced node graphics. F1000Res *3*, 147.

Preibisch, S., Saalfeld, S., and Tomancak, P. (2009). Globally optimal stitching of tiled 3D microscopic image acquisitions. Bioinformatics *25*, 1463-1465.

Ramirez, F., Dundar, F., Diehl, S., Gruning, B.A., and Manke, T. (2014). deepTools: a flexible platform for exploring deep-sequencing data. Nucleic acids research *42*, W187-191.

Rappsilber, J., Mann, M., and Ishihama, Y. (2007). Protocol for micro-purification, enrichment, pre-fractionation and storage of peptides for proteomics using StageTips. Nature protocols *2*, 1896-1906.

Ritchie, M.E., Phipson, B., Wu, D., Hu, Y., Law, C.W., Shi, W., and Smyth, G.K. (2015). limma powers differential expression analyses for RNA-sequencing and microarray studies. Nucleic Acids Res *43*, e47.
Shannon, P., Markiel, A., Ozier, O., Baliga, N.S., Wang, J.T., Ramage, D., Amin, N., Schwikowski, B., and Ideker, T. (2003). Cytoscape: a software environment for integrated models of biomolecular interaction networks. Genome research *13*, 2498-2504.
Shevchenko, A., Tomas, H., Havlis, J., Olsen, J.V., and Mann, M. (2006). In-gel digestion for mass spectrometric characterization of proteins and proteomes. Nature protocols *1*, 2856-2860.
Stadtfeld, M., Maherali, N., Borkent, M., and Hochedlinger, K. (2010). A reprogrammable mouse strain from gene-targeted embryonic stem cells. Nature methods *7*, 53-55.
